# Bioorthogonal release of anticancer drugs *via* gold-triggered 2-alkynylbenzamide cyclization†

**DOI:** 10.1039/d0sc04329j

**Published:** 2020-09-02

**Authors:** Kenward Vong, Tomoya Yamamoto, Tsung-che Chang, Katsunori Tanaka

**Affiliations:** Biofunctional Synthetic Chemistry Laboratory, RIKEN Cluster for Pioneering Research 2-1 Hirosawa Wako-shi Saitama 351-0198 Japan kotzenori@riken.jp kenward.vong@riken.jp; GlycoTargeting Research Laboratory, RIKEN Baton Zone Program 2-1 Hirosawa Wako-shi Saitama 351-0198 Japan; Biofunctional Chemistry Laboratory, A. Butlerov Institute of Chemistry, Kazan Federal University 18 Kremlyovskaya Street Kazan 420008 Russia; Department of Chemical Science and Engineering, School of Materials and Chemical Technology, Tokyo Institute of Technology 2-12-1 O-okayama Meguro-ku Tokyo 152-8552 Japan

## Abstract

Metal-based uncaging of biomolecules has become an emerging approach for *in vivo* applications, which is largely due to the advantageous bioorthogonality of abiotic transition metals. Adding to the library of metal-cleavable protecting groups, this work introduces the 2-alkynylbenzamide (Ayba) moiety for the gold-triggered release of secondary amines under mild and physiological conditions. Studies were further performed to highlight some intrinsic benefits of the Ayba protecting group, which are (1) its amenable nature to derivatization for manipulating prodrug properties, and (2) its orthogonality with other commonly used transition metals like palladium and ruthenium. With a focus on highlighting its application for anticancer drug therapies, this study successfully shows that gold-triggered conversion of Ayba-protected prodrugs into bioactive anticancer drugs (*i.e.* doxorubicin, endoxifen) can proceed effectively in cell-based assays.

## Abstract 2

With the 2-alkynylbenzamide (Ayba) group, this study shows that secondary amines can be released under mild and physiological conditions. Its amenable nature and orthogonality with other metals also allows greater control in prodrug design.

## Graphical abstract

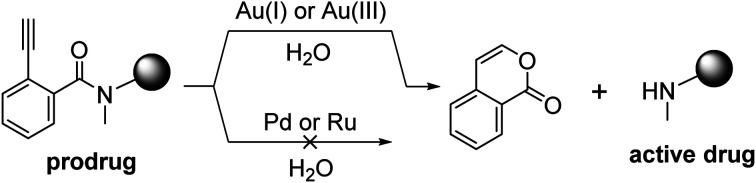

## Introduction

**Fig. 1:**
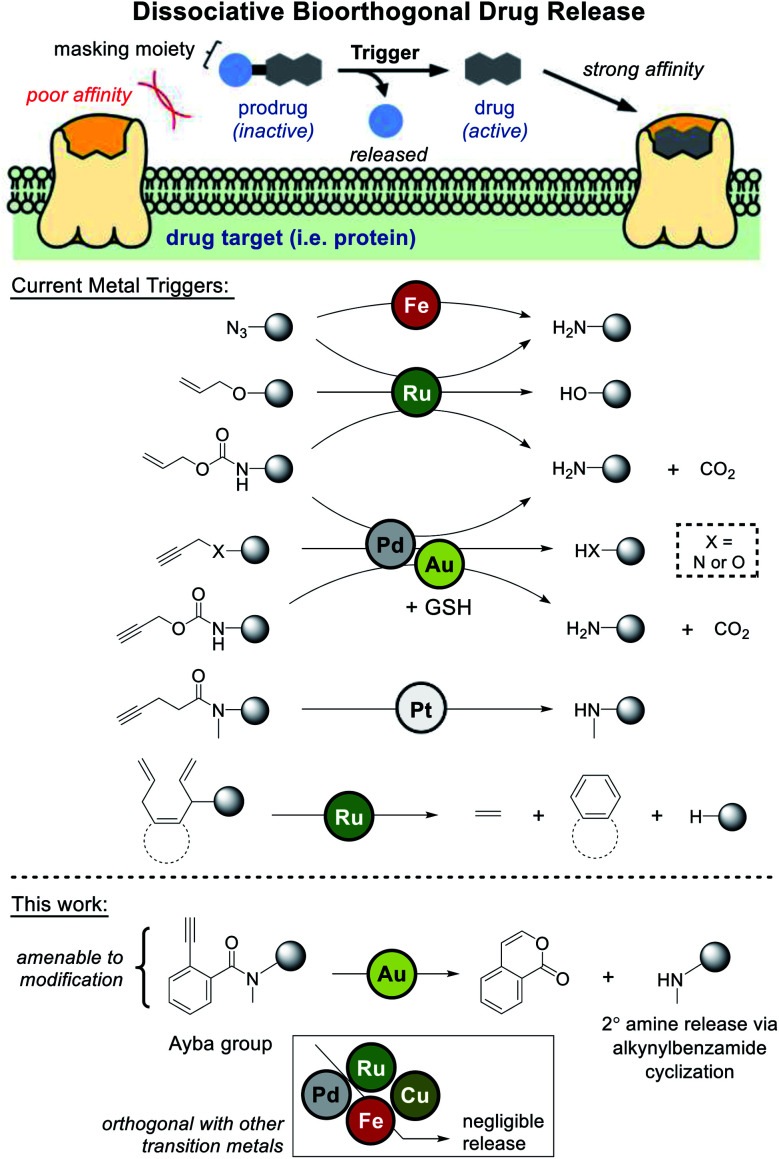
Metal-based triggers for dissociative bioorthogonal drug release have largely employed Fe-, Ru-, and Pd-based reactions. This work presents the release of secondary amine containing drugs *via* gold-triggered 2-alkynylbenzamide cyclization.

Dissociative bioorthogonal reactions are defined as reactions that can activate a substrate (prodrug) *in vivo* to release a bioactive payload (drug) in the presence of an abiotic trigger (Fig. 1).^1,2^ Methodologies can become further viable if the cleavable (masking) moiety renders the prodrug significantly inactive compared to the bioactive agent.

In an emerging field of study, researchers have extensively investigated metal-based uncaging of drugs/fluorophores applicable in cell- or animal-based models.^3^ Mainly employing metals like palladium and ruthenium, a number of examples have leaned heavily on some iteration of amine/alcohol release through masking groups that include azide,^4–7^ allene,^8^ allyl,^9,10^ propargyl,^11–22^ propargyloxycarbonyl (Proc),^19–29^ and allyloxycarbonyl (Alloc).^10–12,28–40^

Looking at current literature, a few areas of need can be clearly identified. First, given the overreliance of palladium- and ruthenium-based uncaging, it would be beneficial to develop drug release reactions based on other transition metals. One relevant example that addresses this issue is the work done by Unciti-Broceta and coworkers, whom discovered that gold catalysts could also be used to facilitate depropargylation *via* glutathione assistance.^41^ However it should be noted that since palladium also facilitates uncaging, these metals cannot be orthogonally used in tandem. Recently, Bernardes and coworkers have also uncovered a method of pentynoyl tertiary amide/*N*-propargyl decaging *via* platinum complexes. Using an elegant combination therapy based on cisplatin, prodrug activation was shown to proceed in zebrafish models.^42^

A further area of need is for masking groups to be amenable to derivatization. For drugs where the protected amine/alcohol is critical for target receptor binding, most of current strategies would work well. However, for drugs where key binding interactions are distributed throughout the molecule, masking groups need to have a greater influence over binding. In one recent study that can likely address this issue, Ward and coworkers developed a “close-to-release” reaction that functions *via* alcohol uncaging triggered by ring-closing metathesis.^43^ In this case, the naphthalene precursor masking groups should be robustly amenable to derivatization.

Considering these points, the primary aim of this study was to develop an amine-releasing reaction that could meet all the following criteria: (1) be compatible under aqueous/physiological conditions, (2) be orthogonally catalyzed by gold without interference from other transition metals (*i.e.* palladium and ruthenium), and (3) possess a masking group that is amenable to derivatization. To do this, 2-alkynylbenzamides (Ayba) protecting groups were thus developed.

## Results

Looking at the literature, 2-alkynylbenzoates first presented themselves as potential gold-activatable masking groups. Originally conceived as a means for glycosylation,^44–46^ later adaptations developed Au(i)/Au(iii) sensing probes that functioned by releasing alcohol-containing fluorophores.^47,48^ Unfortunately, one major shortcoming of this reaction is the susceptibility of esters to hydrolysis.^49^ A search of available literature revealed no successful attempts had been made to replace the ester with an amide, likely due to its added chemical stability. This was confirmed in our own hands as the release of Ayba-protected primary amines was not found to proceed. However, we were encouraged to find that the Au-triggered release of Ayba-protected secondary amines could proceed under mild and aqueous conditions.

As a model reaction, Ayba-deprotection of **1a** to the *N*-methyl amine product **2a** was first monitored under various conditions (Table 1). As expected, increasing levels of **Au-1** under aqueous conditions led to the release of **2a** (7 to 72% yield, entries 2–5). In addition, reactivity was shown to be time and temperature dependent (Table S2 in the ESI†). Further investigations were then done to explore changes to solvent, where reactions carried out in water-miscible solvents generally gave excellent yields (*i.e.* 75–99% for THF, dioxane, methanol), while water-immiscible solvents were poor (2–8% for hexanes, chloroform). These observations can be attributed to the importance of hydrolysis as the last step for amine release. Additionally, tests were done in solutions containing rat serum (15%, entry 16) and DMEM culture media (6%, entry 17) to mimic biological settings. In these cases, the lowered observed yields are likely due to metal quenching from complex biological mixtures.

**Table 1:** Model reaction investigation

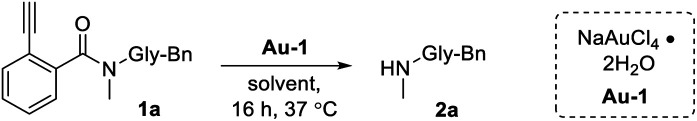			
Entry	Mol%	Solvent	Yield of **2a**^a^ (%)
1	—	10% DMF in PBS buffer	0
2	10	10% DMF in PBS buffer	7
3	25	10% DMF in PBS buffer	18
4	50	10% DMF in PBS buffer	52
5	75	10% DMF in PBS buffer	72
6	50	50% DMF in PBS buffer	7
7	50	50% THF in PBS buffer	17
8	50	50% THF in H_2_O	24
9	50	THF	99
10	50	Dioxane	91 (85)^b^
11	50	Methanol	75
12	50	CHCl_3_	8
13	50	Hexanes	2
14	50	10% DMSO in PBS buffer	57
15	50	10% DMF in MES buffer	21
16	50	1 : 1 : 8 rat serum/DMF/PBS	15
17	50	10% DMF in DMEM media	6

a Yields determined by HPLC (peak retention times compared to product standards, followed by MS analysis for confirmation, and then calculation of yields based on product standard curves).

b Isolated yields obtained by column chromatography purification. All reactions were standardized to 10 μmol of **1a** in 1 ml of solvent (10 mM).

Summarized in Table S3 in the ESI,† a number of Ayba group derivatives were next prepared to explore the factors that contribute to amine release. Important observations of note include the fact that *N*-substitution is critical for facilitating amine release. This is most apparent with the Ayba-protection of primary amines, which showed no detectable levels of Au-triggered release. Another significant observation was that despite numerous derivatizations made to the Ayba moiety, only a few instances saw decreased yields of release. As such, the amenable nature of the Ayba-masking group may potentially be advantageous for designing better non-toxic prodrugs.

In the next step of this study, the effects of different gold metal complexes were investigated. In Table 2, prodrugs **3a**,**m** were tested for the release of the secondary-amine containing drug known as endoxifen. First, the reactivity of Au(iii) complexes were investigated using **Au-2** (prepared as described^50,51^) and **Au-3**. Although good yields were obtained using pure water (Table S4 in the ESI†), observed reactivities experienced a noticeable drop when switched to buffer (*i.e.* PBS buffer pH 7.4). Likely, this effect is brought about by the instability of Au(iii) complexes. As a result, the more stable Au(i) complex **Au-4** was next tested, giving release yields of roughly 60% in PBS buffer for prodrugs **3a**,**m**. Additionally, since amine release is dependent on hydrolysis, it was theorized that increased reactivity is likely correlated with basicity. As a verification, **3a** was also tested under similar conditions with **Au-4** in phosphate buffer pH 8 (77%) and BBS buffer pH 8.5 (80%), both of which gave higher yields.

**Table 2:** Catalyst screening study

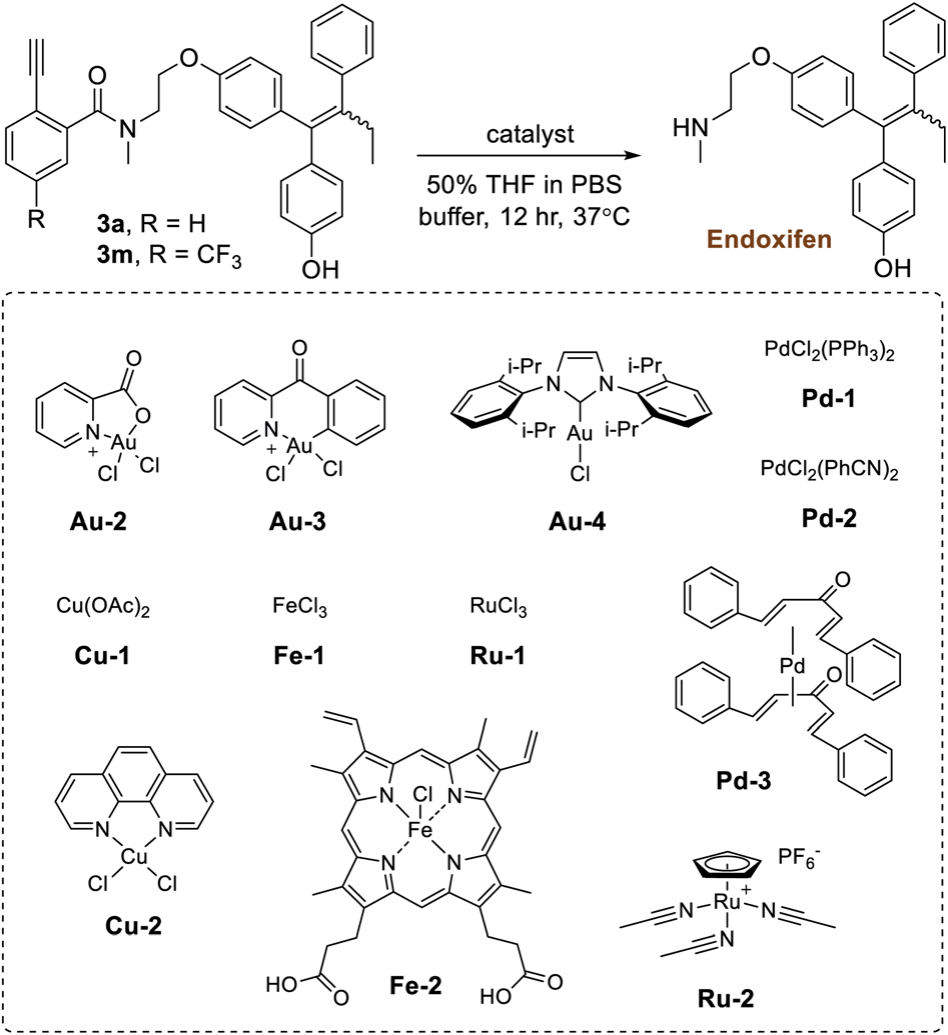			
Entry	Catalyst	Yield of endoxifen release^a^ (%)	
		Substrate **3a**	Substrate **3m**
1	—	n.d.	n.d.
2	**Au-2**	6	3
3	**Au-3**	19	22
4	**Au-4**	60	60
5	**Pd-1**	10	7
6	**Pd-2**	9	5
7	**Pd-3**	5	6
8	**Ru-1**	n.d.	n.d.
9	**Ru-2**	1	1
10	**Cu-1**	n.d.	n.d.
11	**Cu-2**	n.d.	n.d.
12	**Fe-1**	n.d.	n.d.
13	**Fe-2**	n.d.	n.d.

a Yields determined by HPLC (peak retention times compared to product standards, followed by MS analysis for confirmation, and then calculation of yields based on product standard curves). All reactions were standardized to 30 nmol of **3a**,**m** and 30 nmol of catalyst in 50 μl of solvent (600 μM). n.d. = not detectable.

Given the potential benefits of mutually-orthogonal, tandem metal applications,^9^ a further test was conducted to monitor gold-based Ayba-decaging in the presence of other commonly used abiotic transition metals (*i.e.* palladium and ruthenium). In addition, biological metals such as copper and iron were also tested. Shown in Table 2, drug release from prodrugs **3a**,**m** was generally found to be negligible in the given cases (entries 5–13).

**Fig. 2:**
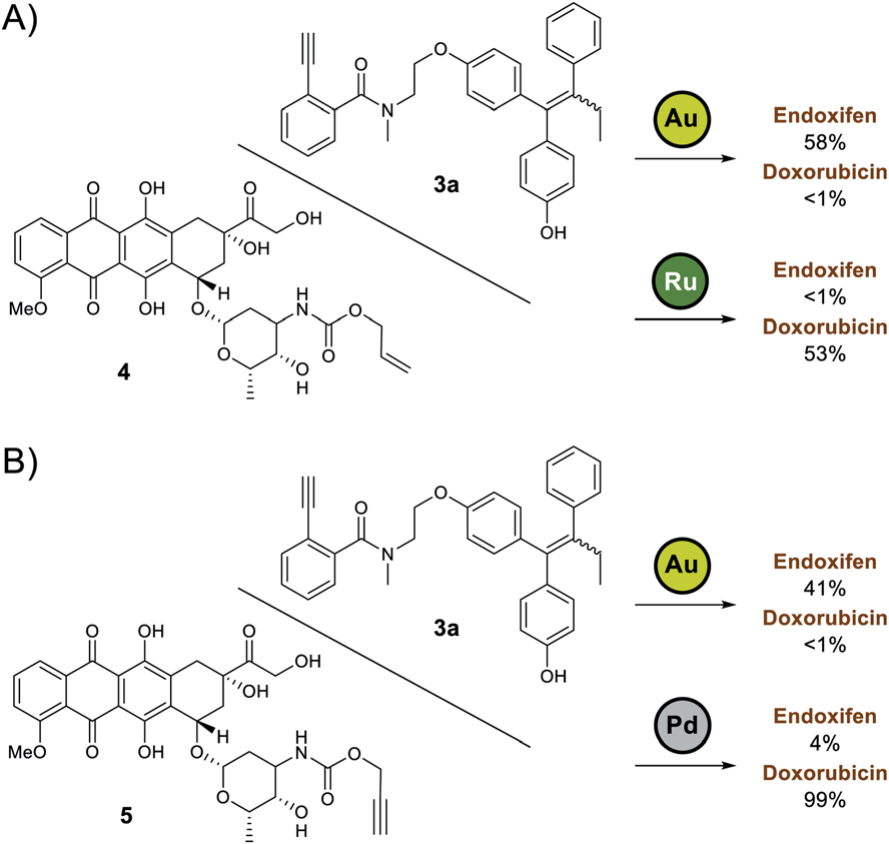
Orthogonal drug release study. (A) To a mixture of Ayba-endoxifen **3a** and Alloc-doxorubicin **4**, addition of **Au-4** led to preferential release of endoxifen while **Ru-2** led to the release of doxorubicin. (B) To a mixture of Ayba-endoxifen **3a** and Proc-doxorubicin **5**, addition of **Au-4** led to preferential release of endoxifen while **Pd-3** led to the release of doxorubicin.

Based on the preliminary data, the capacity of Ayba-protected prodrugs to be used in parallel with other metal-triggered drug release systems was next explored. Alloc-doxorubicin **4** (ruthenium decaged) and Proc-doxorubicin **5** (palladium decaged) were chosen as model alternative prodrugs. For the mixtures of **3a**/**4** (Fig. 2A), observations show that doxorubicin release largely occurs only in the presence of **Ru-2**, while endoxifen release takes place with **Au-4** addition. A similar trend in reactivity was also observed for the mixtures of **3a**/**5** (Fig. 2B).

**Fig. 3:**
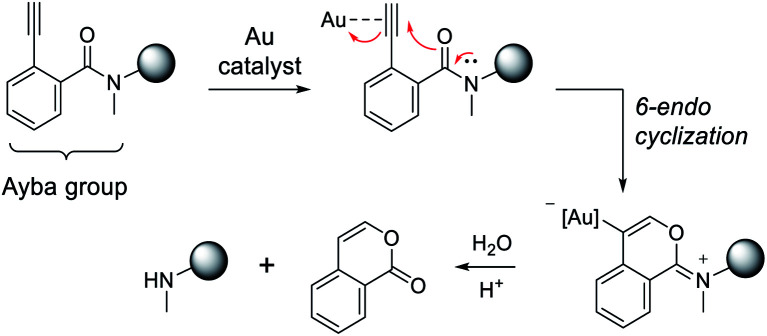
Proposed mechanism for Ayba deprotection *via* Au-catalysed cyclization. Subsequent hydrolysis then leads to the release of a secondary amine.

Overall, the proposed mechanism for gold-triggered alkynylbenzamide cyclization/amine release is depicted in Fig. 3. In this process, gold-activation of the alkynyl group elicits nucleophilic attack from the proximal carbonyl oxygen. Based on the type of ligated Au complex,^52^ 6-endocyclization is favored to generate the oxonium intermediate that is susceptible to base-dependent hydrolysis. As a result, the secondary amine-containing molecule can then be released in conjunction with a corresponding isocoumarin derivative. To reason why gold-triggered release is favored for secondary amines over primary amines, it can be theorized that the alpha effects of the *N*-substituted methyl group enhances the nucleophilicity of the carbonyl oxygen. This rationale would be consistent with a similar report that showed the presence of a Weinreb amide can increase rates of *N*-alkoxy-2-alkynylbenzamide cyclization *via* copper catalysis.^53^ To address the poor catalytic activity, it may be hypothesized that hydrolysis is a limiting factor under physiological conditions. Also, Au(i) disproportionation could play a factor in the decomposition of the gold complexes.^54^

In the next part of this study, the focus shifted to investigating the viability of Ayba-based prodrugs as anticancer therapeutics. Designed to directly release endoxifen, the cytotoxic profiles of prodrugs **3a**,**m** were first tested against ER-positive MCF7 breast cancer cells (Table S5 and Fig. S16 in the ESI†). However, only a low difference in EC_50_ values between prodrugs **3a**,**m** (55–57 μM) and endoxifen (25 μM) was observed. Given the moderate cytotoxicity of the parent drug endoxifen in these trials, the desire thus shifted to search for an alternative and more robust anticancer drug.

**Fig. 4:**
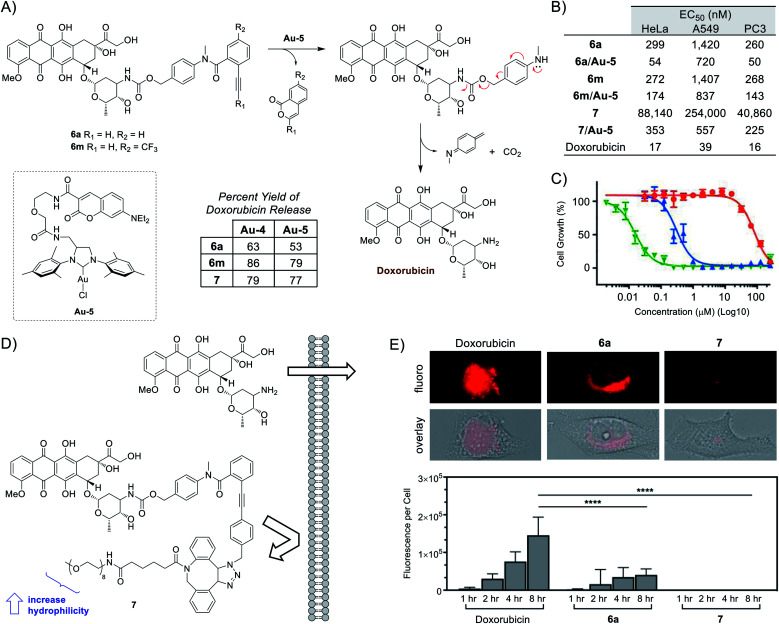
Ayba-based doxorubicin prodrugs. (A) Mechanism of indirect release (*via* PMBC spacer) of primary amine-containing doxorubicin from **6a**,**m**. Reactions were standardized to 30 nmol of prodrug and 30 nmol of catalyst in 50 μl of THF/PBS buffer pH 7.4 (600 μM). (B) Summary of cytotoxic activity (EC_50_ values) against various cancer cell lines. (C) Example growth curves for HeLa cancer cells incubated with either prodrug **7** (red), mixture **7**/**Au-5** (blue), or doxorubicin (green). (D) Depiction of the strategy to prevent passive diffusion of prodrug **7** by increasing bulk and hydrophilicity. (E) Imaging studies to investigate the cellular penetration of doxorubicin, **6a**, and **7** (10 μM) into HeLa cancer cells over time.

To bypass the constraints of secondary-amine containing drug usage, the final portion of this study focused on adapting the use of *p*-methylamino-benzyloxycarbonyl (PMBC) spacers, which are also capable of undergoing spontaneous 1,6-elimination to release an attached amine.^55^ As depicted in Fig. 4A, compounds **6a**,**m** were synthesized and tested for the release of the anticancer agent doxorubicin. Under aqueous *in vitro* conditions, doxorubicin release was found to range from 63–86% for **Au-4** and 53–79% for **Au-5**.

To carry out subsequent cell-based experiments, a preference was made to utilized **Au-5** due to its improved solubility in 1% DMSO compared to **Au-4**. Additionally, control experiments (Fig. S15 in the ESI†) determined that a final concentration of 10 μM **Au-5** would be non-toxic under all cell assay conditions. As shown in Fig. 4B and S17–S19 in the ESI,† addition of **Au-5** to Ayba-protected prodrugs were all found to help increase their cytotoxic activity. For example, gold-activation of prodrug **6a** led to a drop in EC_50_ values for several cancer cells lines; 299 to 52 nM for HeLa, 1420 to 720 nM for A549, and 260 to 50 nM for PC3.

From the collected data, the intrinsic cytotoxicity of prodrugs **6a**,**m** (260–1420 nM) compared to the active drug (16–39 nM) is at most about a 16–36 fold difference. To explain this modest difference, it should be noted that anthracyclines (*i.e.* doxorubicin) are generally known to enter cells through passive diffusion,^56^ followed by nuclear transport through proteasome binding and subsequent DNA intercalation.^57,58^ Given this mechanism, it was theorized that the hydrophobic nature of the unsubstituted Ayba masking group is unlikely to have a substantial effect in preventing cellular uptake of doxorubicin-based prodrugs.

With the opportunity for derivatization, the freedom exists to manipulate the molecular properties of Ayba-protected prodrugs on a situational basis. Therefore, as depicted in Fig. 4D, compound **7** was prepared with the reasoning that cellular uptake could be prevented *via* controlling size (increase bulk) and polarity (increase hydrophilicity), both of which are factors known to govern passive diffusion.

In order to study cellular uptake, imaging studies were performed with HeLa cells incubated with either doxorubicin, **6a**, or **7** (Fig. 4E). As anticipated, the fastest and highest levels of uptake occurred with doxorubicin. In this case, fluorescence is strongly distributed throughout the cytoplasm and nucleus. For the unsubstituted Ayba-prodrug **6a**, fluorescence was instead detectable only within the cytoplasm while being faint/absent in the nucleus. This observation could be interpreted as nuclear transport (*via* proteasome binding) being affected by the presence of the Ayba group. Finally, imaging studies conducted with the PEG-containing prodrug **7** was shown to be poorly uptaken by HeLa cells under all timepoints. Given the significant impairment of cellular uptake, it thus becomes apparent why the EC_50_ values of prodrug **7** (41–254 μM) are so high compared to doxorubicin, which effectively translates to about a 2554–6512 fold difference. Ultimately, this translated to more effective biological effects of the **7**/**Au-5** mixture, where gold-activation of prodrug **7** led to significant drops in EC_50_ values for the tested cancer cells lines; 88 to 0.4 μM for HeLa, 254 to 0.6 μM for A549, and 41 to 0.2 μM for PC3.

## Conclusions

In conclusion, this study demonstrates the design and development of the 2-alkynylbenzamide (Ayba) group for gold-dependent release of secondary amines under mild and physiological conditions. Although not shown to proceed catalytically, one of the principal benefits of the Ayba protecting group is that decaging can be triggered *via* gold complexes that proceed orthogonally in the presence of transition metals like palladium and ruthenium. Furthermore, this study showed that the amenable nature of the Ayba group can allow for the manipulation of prodrugs properties, such as size and lipophilicity. Overall, we believe this work will be a useful addition to the growing library of metal-triggered dissociative bioorthogonal reactions, which can serve as a versatile means to uncage primary/secondary amines for biological applications or for developing future drug therapies.

## Conflicts of interest

There are no conflicts to declare.

## Supplementary Material

SC-011-D0SC04329J-s001
