# Supplementary material for: Bioorthogonal release of anticancer drugs via gold-triggered 2-alkynylbenzamide cyclization
Source: Chem Sci. 2020 Sep 2;11(40):10928–33. doi: 10.1039/d0sc04329j (PMC8162444; doi:10.1039/d0sc04329j)
Supplement: SC-011-D0SC04329J-s001 [file SC-011-D0SC04329J-s001.pdf]

Electronic Supplementary Material (ESI) for Chemical Science.
This journal is © The Royal Society of Chemistry 2020

Supplementary Information

Table of Contents

1. Reactivity Studies 2
1.1 General Information 2
1.2 HPLC Methods 2
1.3 HPLC Analysis (Product Standard Curve and Calibration) 5
1.4 Reactivity Study 10
1.4.1 Protocols and Yield Determination 10
1.4.2 Reactivity Data 12
1.4.3 By-product Identification 15
1.5 Synthetic Schemes 16
1.5.1 Reactivity Study Substrates 16
1.5.2 Endoxifen Derivatives 46
1.5.3 Doxorubicin Derivatives 49
1.5.4 Gold Catalyst Complexes 60
1.5.5 Reaction by-products 63
2. Cell-based Assays 64
2.1 General Cell Culture Protocol 64
2.2 Cell Viability Studies 64
2.2.1 MTS Assay Protocol 64
2.2.2 Controls 64
2.2.3 Cell Toxicity Results 66
2.3 Cell Permeability Studies 69
2.3.1 Incubation and Imaging Protocol 69
2.3.2 Cell Imaging Results 69
3. References 76

1. Reactivity Studies

1.1 General Information

All reagents and buffer components were purchased from Sigma-Aldrich, Fisher Scientific, Alfa Aesar, TCI chemicals, or Wako Chemicals without further purification. Notable reagents and their commercial source include Endoxifen (AdooQ BioScience), Doxorubicin Hydrochloride (TCI chemicals), Chloro(1,3-bis(2,6-diisopropylphenyl)imidazol-2-ylidene)gold(I) (TCI chemicals), Dichloro(2-pyridinecarboxylato)gold (Sigma-Aldrich), and Sodium tetrachloroaurate(III) dihydrate (Sigma-Aldrich)

TLC analysis (F-254) were performed with 60 Å silica gel from Merck Millipore. ^{1}H and ^{13}C NMR spectra were measured on either a JEOL AL300 (300 MHz) or AL400 (400 MHz) instrument with the solvent peaks as internal standards. High-resolution mass spectroscopy (HRMS) was carried out on a Bruker MicroTOF-QIII electrospray ionization time-of-flight (ESI-TOF) mass spectrometer.

1.2 HPLC Methods

Reverse-phase HPLC was used to analyze and purify compounds as indicated. The Shimadzu system (Kyoto, Japan) employed two LC-20AP pumps outfitted with a SPD-20AV photodiode array detector, a RF-10AXL fluorescence detector, and a SIL-20A autosampler. The columns used were a semi-preparative 10 × 250 mm Cosmosil $5C_{18}$-Ar-300 and an analytical 4.6 × 250 mm Cosmosil $5C_{18}$-Ar-300 from Nacalai Tesque (Kyoto, Japan). Samples were eluted using a combination of mobile phases A (100% H_2O), B (100% acetonitrile), C (H_2O with 0.1% TFA), and D (acetonitrile with 0.1% TFA). The detector was set to 214 and 254 nm. The different elution methods that were employed in this study are shown in Table S1.

Table S1. Gradient profiles for HPLC studies

	Flow rate (ml/min)	Time (min)	%A (100% H_2O)	%B (100% ACN)	%C (H_2O with 0.1% TFA)	%D (ACN with 0.1% TFA)
Method 1	3.0	0			95	5
Semi-prep		10			95	5
		40			30	70
		43			1	99
		50			1	99
		58			95	5
		60			95	5
Method 2	3.0	0			90	10
Semi-prep		10			90	10
		40			30	70
		43			1	99
		50			1	99
		58			90	10
		60			90	10

Table S1 (continued). Gradient profiles for HPLC studies

	Flow rate (ml/min)	Time (min)	%A (100% H_2O)	%B (100% ACN)	%C (H_2O with 0.1% TFA)	%D (ACN with 0.1% TFA)
Method 3	3.0	0			80	20
Semi-prep		10			80	20
		40			30	70
		43			1	99
		50			1	99
		58			80	20
		60			80	20
Method 4	3.0	0			70	30
Semi-prep		10			70	30
		40			30	70
		43			1	99
		50			1	99
		58			70	30
		60			70	30
Method 5	1.0	0			95	5
Analytical		10			95	5
		35			60	40
		40			5	95
		45			5	95
		50			95	5
		55			95	5
Method 6	3.0	0	60	40		
Semi-prep		5	60	40		
		28	5	95		
		32	5	95		
		33	60	40		
		35	60	40		
Method 7	3.0	0	65	35		
Semi-prep		5	65	35		
		28	5	95		
		32	5	95		
		33	65	35		
		35	65	35		

Table S1 (continued). Gradient profiles for HPLC studies

	Flow rate (ml/min)	Time (min)	%A (100% H_2O)	%B (100% ACN)	%C (H_2O with 0.1% TFA)	%D (ACN with 0.1% TFA)
Method 8	3.0	0	85	15		
Semi-prep		3	85	15		
		5	60	40		
		26	50	50		
		28	25	75		
		32	25	75		
		33	85	15		
		37	85	15		
Method 9	3.0	0	70	30		
Semi-prep		3	70	30		
		18	25	75		
		22	25	75		
		23	70	30		
		27	70	30		
Method 10	3.0	0	90	10		
Semi-prep		3	90	10		
		13	75	25		
		30	10	90		
		35	90	10		
		40	90	10		
Method 11	3.0	0	70	30		
Semi-prep		3	70	30		
		18	10	90		
		22	10	90		
		23	70	30		
		27	70	30		
Method 12	3.0	0	65	35		
Semi-prep		3	65	35		
		18	10	90		
		22	10	90		
		23	65	35		
		27	65	35		

1.3 HPLC Analysis (Product Standard Curve and Calibration)

To determine yields from HPLC analysis, calibration curves were constructed using product standards of known amounts (shown in Figures S1-S10).

Product Structure	HPLC Method (see Table S1)	Calibration Curve

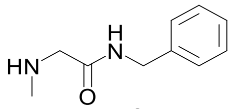

2a

Method 1

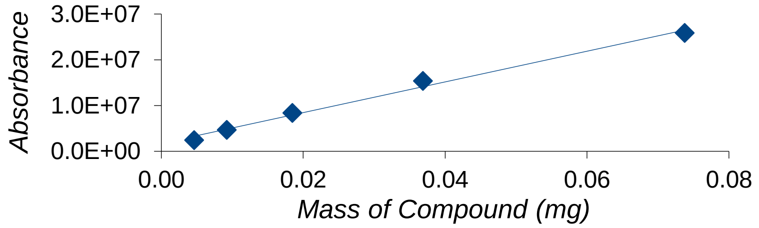

**R^2 = 0.9921**	Value	Error (σ)
Slope	335496372.9723	17336657.9872
Intercept	1740214.3807	659932.6452

HPLC trace:

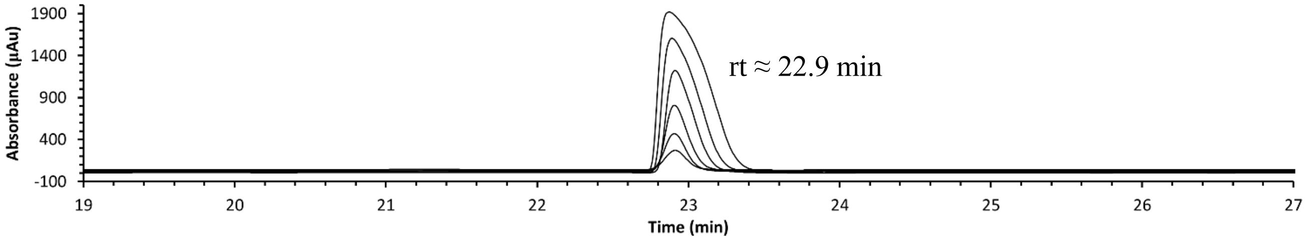

Figure S1. HPLC calibration curve of product **2a**

Product Structure	HPLC Method (see Table S1)	Calibration Curve

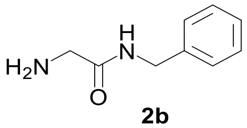

2b

Method 1

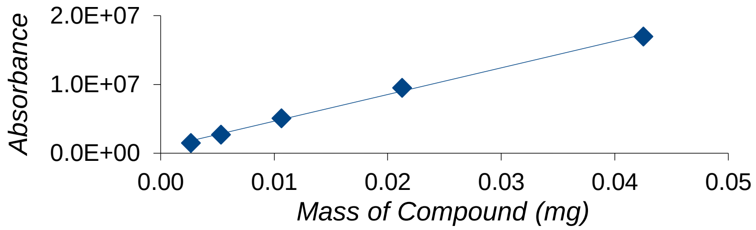

**R^2 = 0.9970**	Value	Error (σ)
Slope	387691152.9412	12200018.6890
Intercept	780172.1250	267621.8883

HPLC trace:

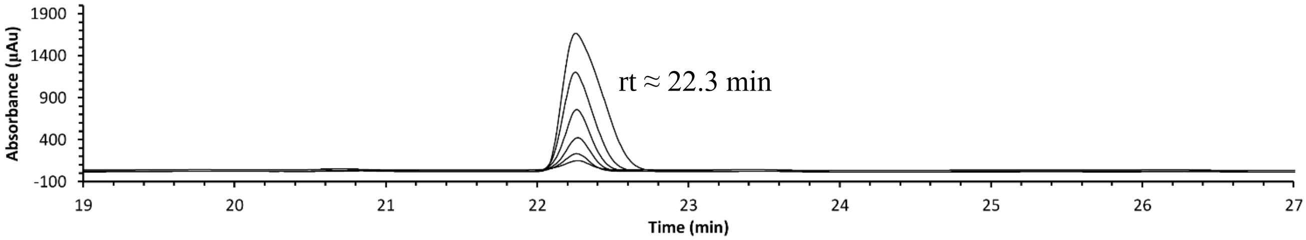

Figure S2. HPLC calibration curve of product **2b**

Product Structure	HPLC Method (see Table S1)	Calibration Curve
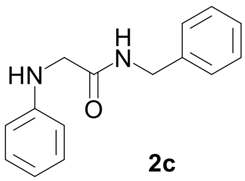 **2c**	Method 4	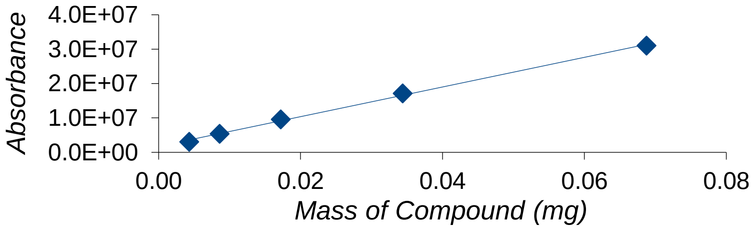

**R^2 = 0.9982**	Value	Error (σ)
Slope	431217757.3455	10529673.9466
Intercept	1754655.0020	373645.5652

HPLC trace:

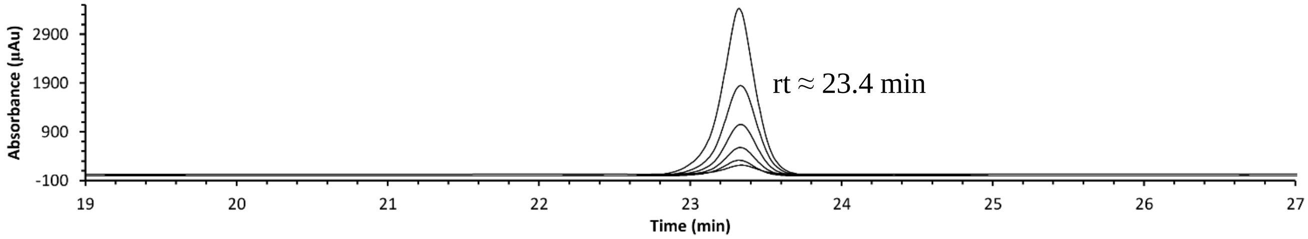

Figure S3. HPLC calibration curve of product **2c**

Product Structure	HPLC Method (see Table S1)	Calibration Curve
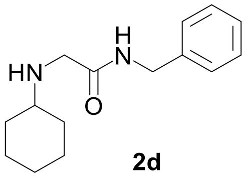 **2d**	Method 3	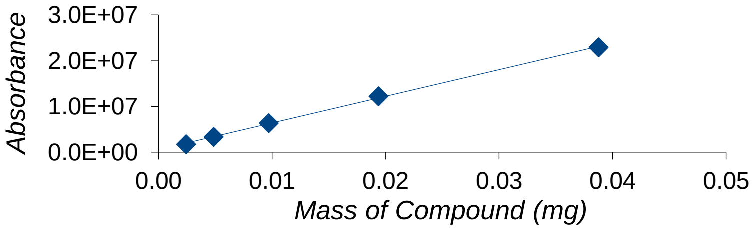

**R^2 = 0.9991**	Value	Error (σ)
Slope	582321369.2265	10132466.0030
Intercept	570267.3079	202655.8148

HPLC trace:

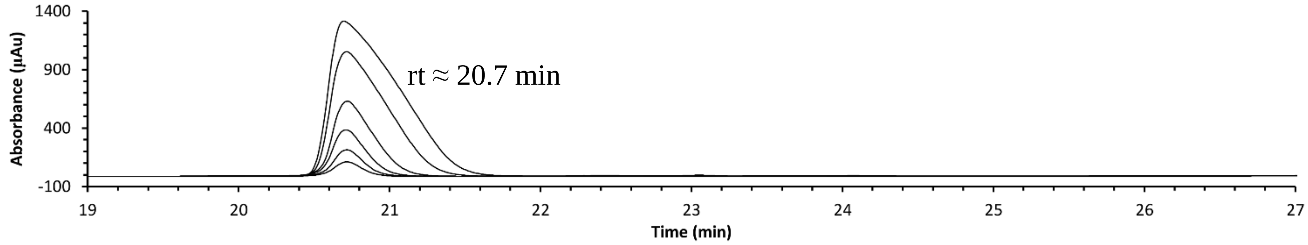

Figure S4. HPLC calibration curve of product **2d**

Product Structure	HPLC Method (see Table S1)	Calibration Curve
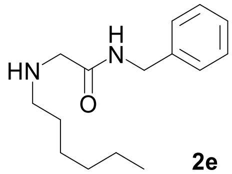 **2e**	Method 3	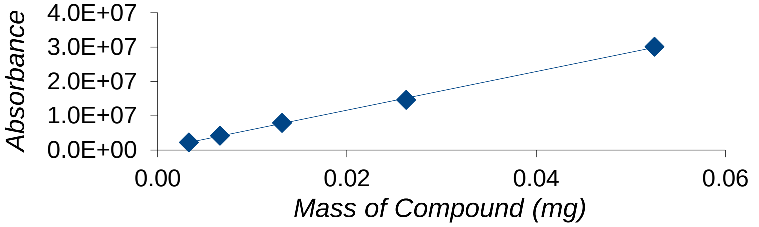

R^2 = 0.9994	Value	Error (σ)
Slope	562496460.8295	8120349.9246
Intercept	369195.6250	220042.3389

HPLC trace:

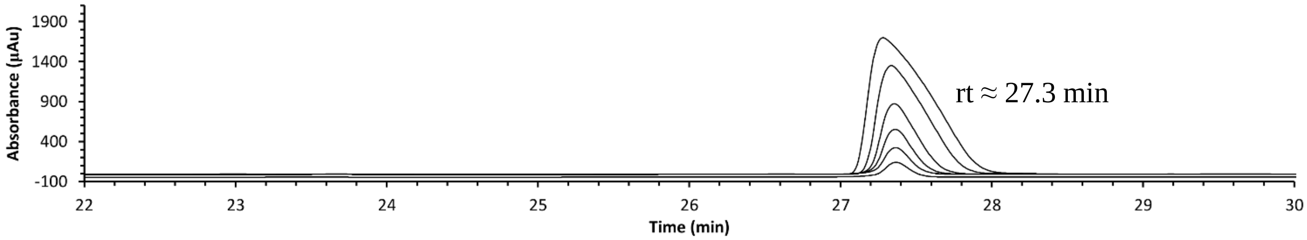

Figure S5. HPLC calibration curve of product **2e**

Product Structure	HPLC Method (see Table S1)	Calibration Curve
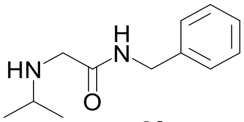 **2f**	Method 2	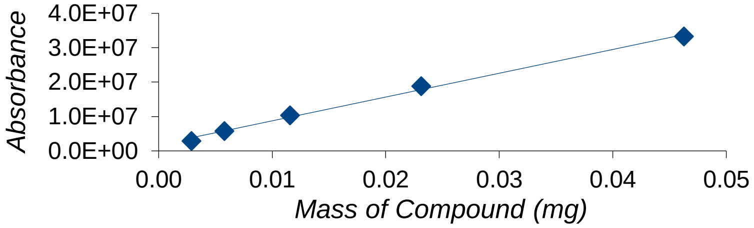

R^2 = 0.9959	Value	Error (σ)
Slope	691725696.7945	25734644.6144
Intercept	1823430.8360	614330.5589

HPLC trace:

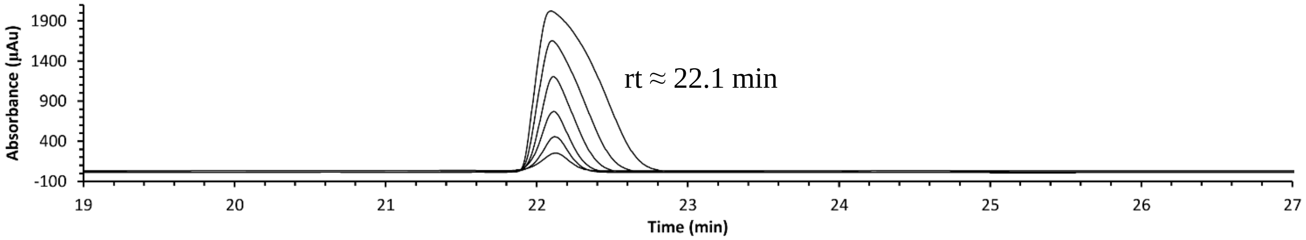

Figure S6. HPLC calibration curve of product **2f**

Product Structure	HPLC Method (see Table S1)	Calibration Curve

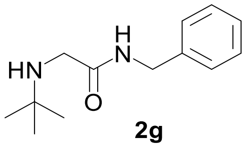

2g

Method 2

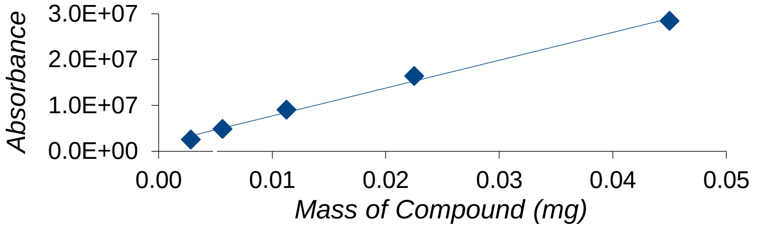

**R^2 = 0.9944**	Value	Error (σ)
Slope	607301239.1876	26218199.3523
Intercept	1646220.0417	608958.3359

HPLC trace:

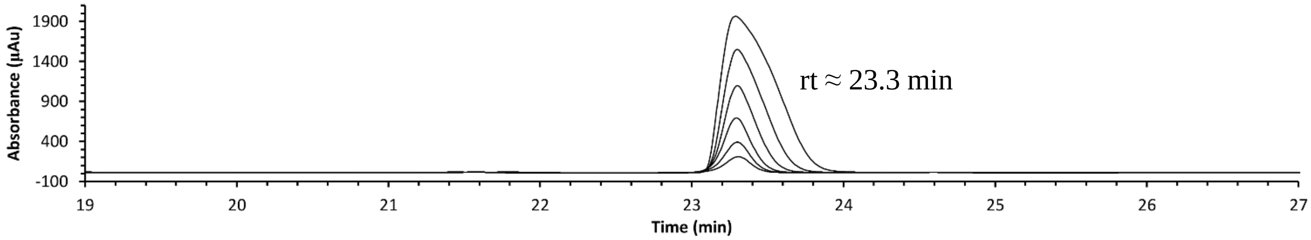

Figure S7. HPLC calibration curve of product **2g**

Product Structure	HPLC Method (see Table S1)	Calibration Curve

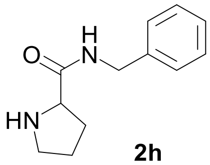

2h

Method 1

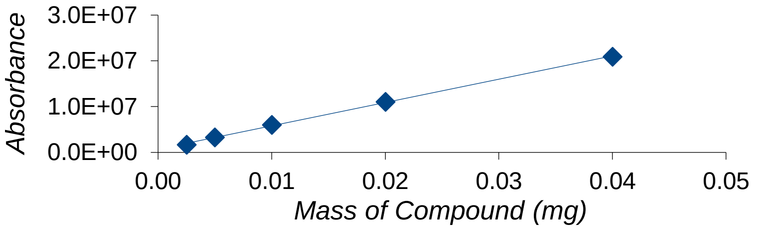

**R^2 = 0.9992**	Value	Error (σ)
Slope	509497867.7419	8156427.6234
Intercept	709388.2500	168396.1596

HPLC trace:

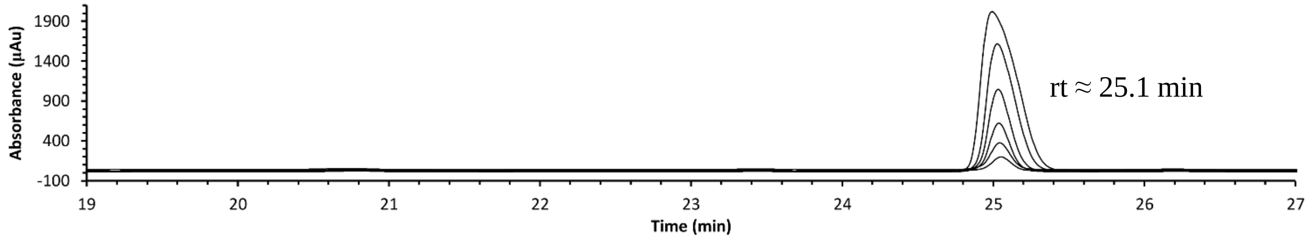

Figure S8. HPLC calibration curve of product **2h**

Product Structure	HPLC Method (see Table S1)	Calibration Curve
Doxorubicin · HCl	Method 5	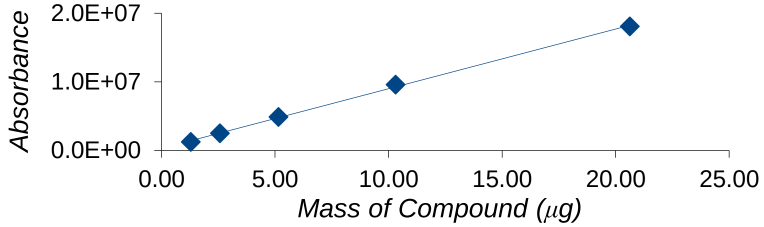

R^2 = 0.9992	Value	Error (σ)
Slope	869618.7256	15506.3324
Intercept	301875.2917	165072.6983

HPLC trace:

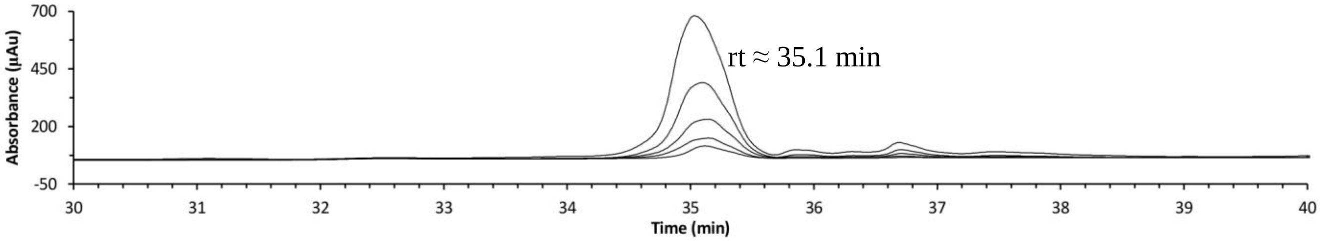

Figure S9. HPLC calibration curve of product Doxorubicin

Product Structure	HPLC Method (see Table S1)	Calibration Curve
Endoxifen (E/Z)	Method 5	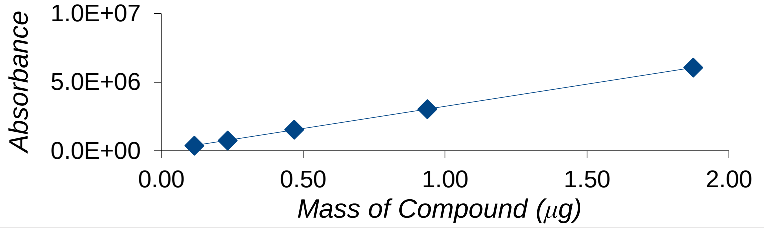

R^2 = 1.0000	Value	Error (σ)
Slope	3229026.0186	5376.9124
Intercept	7719.5833	5203.6303

HPLC trace:

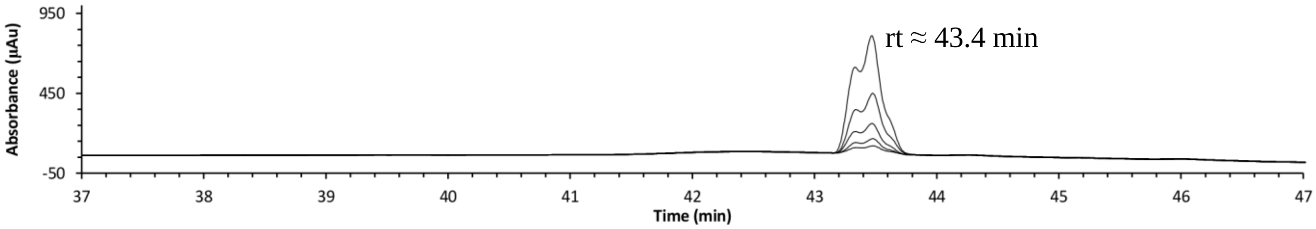

Figure S10. HPLC calibration curve of product Endoxifen

1.4 Reactivity Study

1.4.1 Protocols and Yield Determination

In general, product identification was determined by MS analysis and comparison with retention times of known product standards. Yields were obtained via HPLC analysis (unless otherwise noted). This was carried out via peak integration, followed by calculations based on product calibration curves shown in Supplementary Section 1.3.

For the model reactivity and derivatization studies, compounds **1a-t** (0.01 mmol) and **Au-1** (concentration adjusted as indicated) were dissolved in solvent (1 ml). The mixtures (in glass test tubes) were then placed on a block temperature-controlled hotplate stirrer (Asynt, UK). Reactions were stirred at the indicated times and temperature. To workup, mixtures were diluted with acetonitrile containing 0.1% TFA (1 ml), filtered, and then injected (50 µl) onto a HPLC with an autosampler. Depending on the desired product, HPLC methods 1-4 were used.

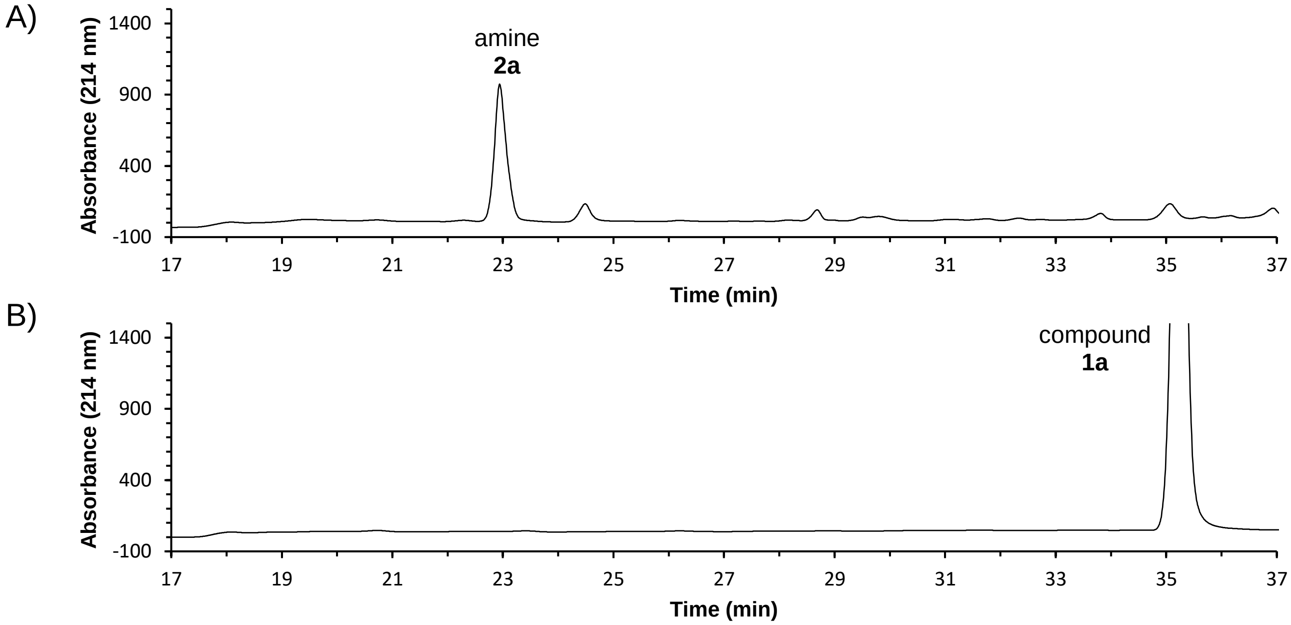

Figure S11. A) An example of HPLC trace taken from the reaction mixture of **1a** incubated with **Au-1**. Identified is the released amine product **2a**. B) HPLC trace of the starting material, compound **1a**.

For the individual reactivity studies, prodrugs **3a**, **3m**, **6a**, **6m**, or **7** (30 nmol) and metal catalyst (30 nmol) were dissolved in 50% THF in aqueous solvent (50 µl).

For the orthogonal metal reactivity studies, a mixture of prodrug **3a** (30 nmol) and either prodrug **4** or **5** (30 nmol) was made in 50% THF in PBS buffer pH 7.4 (50 µl). Depending on the conditions, either **Au-4** (30 nmol), **Ru-2** (30 nmol), or **Pd-3** (30 nmol) was then added.

In general, the mixtures (in eppendorf tubes) were placed in a MD-MINI water bath incubator (Major Science, USA). Without stirring, reactions were left to incubate for 12 hr at 37°C. To workup, mixtures were diluted with 1 mM dodecanethiol in THF (100 µl) to quench the metal catalysts. Since doxorubicin is insoluble unless its primary amine is protonated, 1M HCl (50 µl) was added to ensure the released compound would match the commercial product standards (i.e. doxorubicin

hydrochloride). The solutions were then filtered, and injected (50 µl) onto a HPLC with an autosampler using HPLC method 5.

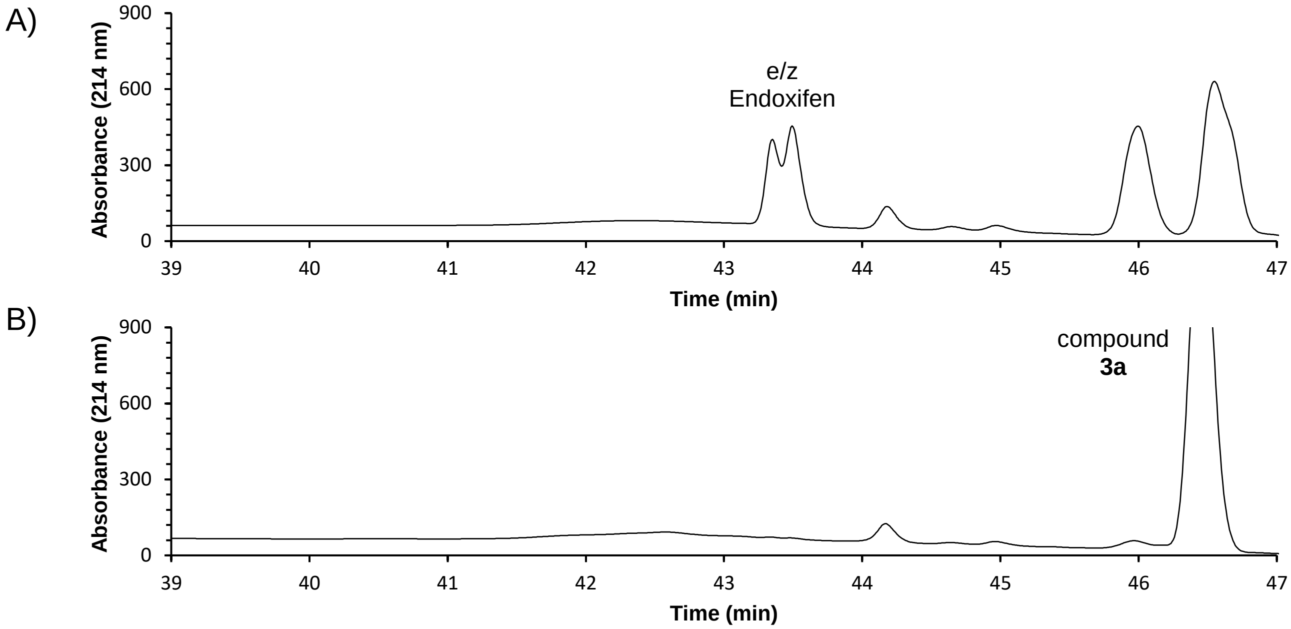

Figure S12. A) An example of HPLC trace taken from the reaction mixture of **3a** incubated with **Au-4**. Identified is the released e/z Endoxifen. B) HPLC trace of prodrug **3a**.

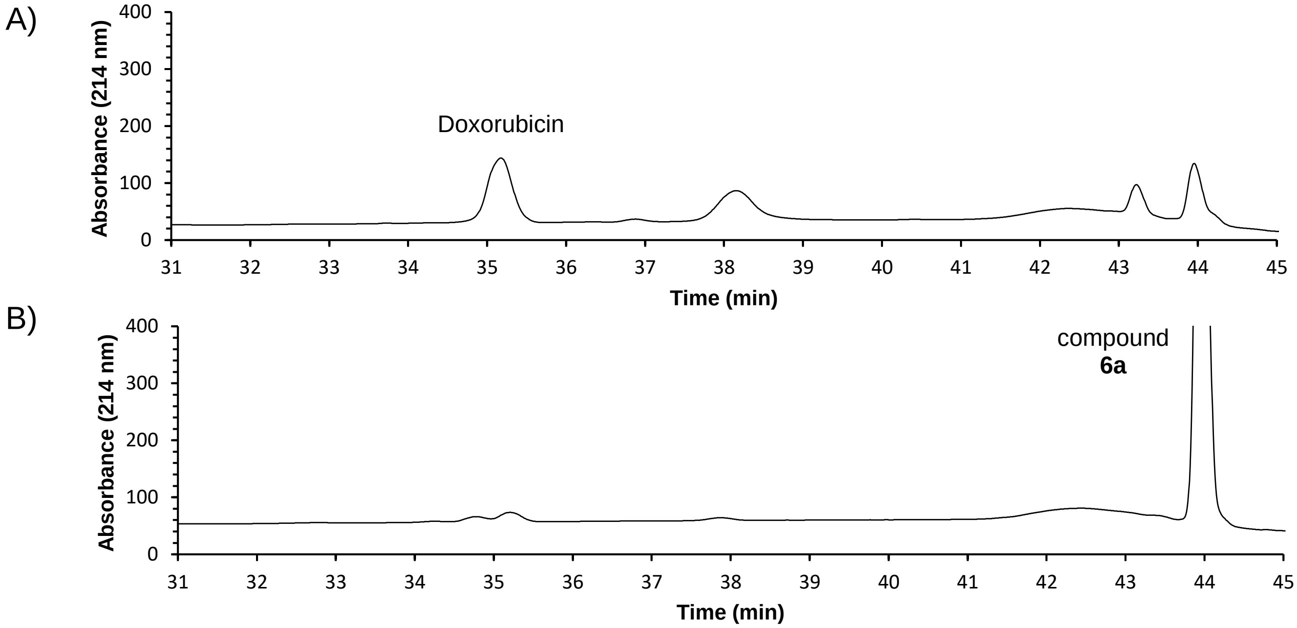

Figure S13. A) An example of HPLC trace taken from the reaction mixture of **6a** incubated with **Au-4**. Identified is the released Doxorubicin. B) HPLC trace of prodrug **6a**.

1.4.2 Reactivity Data

In Table S2, the model reaction from **1a** to **2a** was shown to be time-dependent (entries 1-3) and temperature-dependent (entries 4-5).

Table S2. Investigation into time and temperature dependence

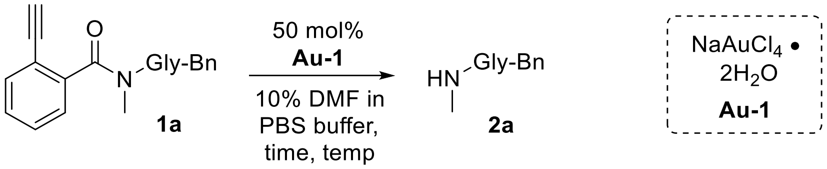

Entry	Time (hr)	Temp (°C)	Yield of **2a** (%)[a]
1	1	37	19
2	3	37	44
3	6	37	54
4	16	rt	46
5	16	70	69

[a]Yields determined by HPLC (peak retention times compared to product standards, followed by MS analysis for confirmation, and then calculation of yields based on product standard curves). [b]Isolated yields obtained by column chromatography purification. All reactions were standardized to 10 µmol of **1a** in 1 ml of solvent (10 mM).

In Table S3, derivatives **1b-t** were prepared and tested to investigate the factors that could possibly influence amine release.

Alkyne substitution (R_1 position): Pentyl, phenyl, and anisole derivatives **1q-s** were tested, which all largely showed an increase in reactivity. The only example that falls from this pattern is **1t** (entry 20), which may possibly be due to the amine substituent perturbing reactivity by interacting with the Au-catalyst.

Benzyl substitution (R_2, R_3, and R_4 positions): Electron-withdrawing group were shown to increase yields of amine release. From comparing simple fluoro substitutions either at the R_2, R_3, and R_4 positions (entries 9-11), it can be observed that the R_3 position (para to the alkyne moiety) gave slightly better yields. Subsequently stronger electron-withdrawing groups at the R_3 position, such as nitro **1l** (56%, entry 12) and trifluoromethyl **1m** (74%, entry 13), gave higher yields of amine release. No significant changes in reactivity were seen with substitutions that were weakly electron-donating (methyl, entry 14) and moderately electron-donating (methoxy, entries 15-16). A possible explanation for these observations is that para-positioned electron withdrawing groups likely has a stronger effect in pulling electron density away from the alkyne, thereby better activating it for Au-catalyzed cyclization.

N-substitution (R_5 position): Unsubstituted amide **1b** showed no detectable levels of Au-triggered amine release (entry 2). Longer and bulkier alkyl groups generally led to lower yields of amine release (c-hexyl, hexyl, isopropyl, and t-butyl, entries 4-7). The same observation was also seen by replacement with an aromatic group (phenyl, entry 3) and an intramolecularly-linked alkane (proline-based amine, entry 8). Likely, the observed reductions in amine release are the outcome of steric bulk outweighing the alpha effects of *N*-substitution.

Table S3. Reactivity of Ayba group derivatizes

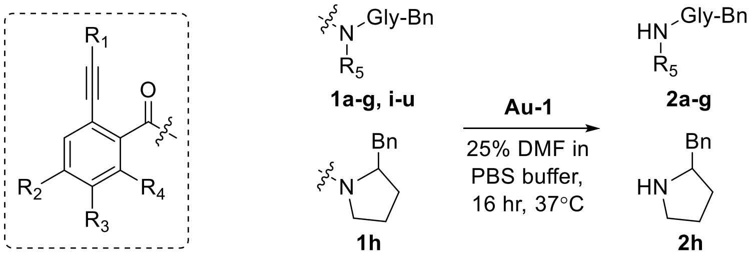

Entry	SM	R_1	R_2	R_3	R_4	R_5	Prod	Yield of **2a-h** (%)[a]
1	**1a**	H	H	H	H	CH_3	**2a**	36
2	**1b**	H	H	H	H	H	**2b**	0
3	**1c**	H	H	H	H	Ph	**2c**	13
4	**1d**	H	H	H	H	c-hexyl	**2d**	15
5	**1e**	H	H	H	H	hexyl	**2e**	20
6	**1f**	H	H	H	H	iPr	**2f**	11
7	**1g**	H	H	H	H	t-butyl	**2g**	5
8	**1h**	H	H	H	H		**2h**	21
9	**1i**	H	F	H	H	CH_3	**2a**	45
10	**1j**	H	H	F	H	CH_3	**2a**	47
11	**1k**	H	H	H	F	CH_3	**2a**	41
12	**1l**	H	H	NO_2	H	CH_3	**2a**	56
13	**1m**	H	H	CF_3	H	CH_3	**2a**	74
14	**1n**	H	CH_3	CH_3	H	CH_3	**2a**	43
15	**1o**	H	OMe	H	H	CH_3	**2a**	56
16	**1p**	H	H	OMe	H	CH_3	**2a**	45
17	**1q**	pentyl	H	H	H	CH_3	**2a**	62
18	**1r**	Ph	H	H	H	CH_3	**2a**	50
19	**1s**	4-OMe-Ph	H	H	H	CH_3	**2a**	48
20	**1t**	4-NH_2- Ph	H	H	H	CH_3	**2a**	30

[a]Yields determined by HPLC (peak retention times compared to product standards, followed by MS analysis for confirmation, and then calculation of yields based on product standard curves). All reactions were standardized to 10 µmol of **1a-t** and 5 µmol of **Au-1** in 1 ml of solvent (10 mM).

In Table S4, the reactivity of the tested metal catalyst complexes (**Au-2**, **Au-3**, **Au-4**) were compared under aqueous conditions containing either pure water or PBS buffer pH 7.4. Due to the instability of **Au-2** and **Au-3** complexes, both **3a** and **3m** experienced a noticeable drop in reactivity when switching to buffered conditions.

Table S4. Investigation into the stability between Au(I) and Au(III) catalysts

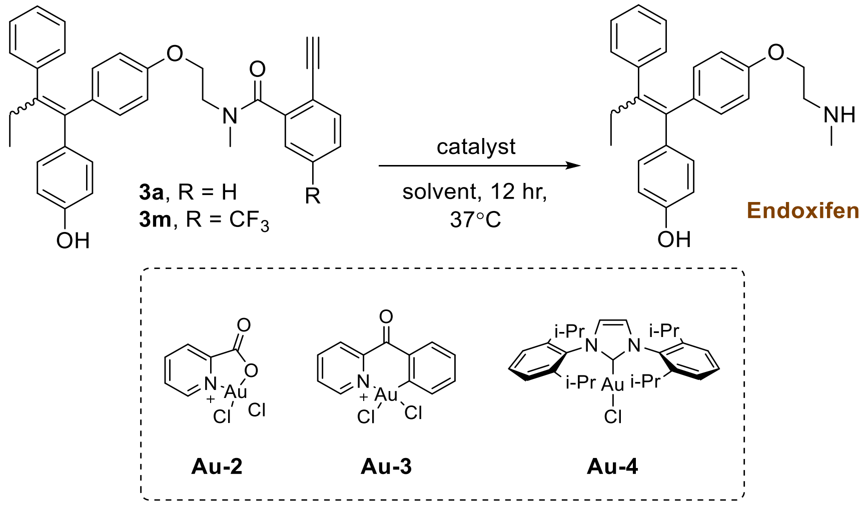

Entry	Substrate	Catalyst	Yield of Release (%)[a]	
			50% THF in H_2O	50% THF in PBS buffer
1	**3a**	-	<1	<1
2	**3a**	**Au-2**	37	6
3	**3a**	**Au-3**	44	19
4	**3a**	**Au-4**	20	60
5	**3m**	-	<1	<1
6	**3m**	**Au-2**	60	3
7	**3m**	**Au-3**	62	22
8	**3m**	**Au-4**	60	60

[a]Yields determined by HPLC (peak retention times compared to product standards, followed by MS analysis for confirmation, and then calculation of yields based on product standard curves). All reactions were standardized to 30 nmol of **3a,m** and 30 nmol of catalyst in 50 µl of solvent (600 µM).

1.4.3 By-product Identification

For Ayba-decaging to occur, the initial transformation involves a nucleophilic cyclization process where a bond-forming interaction occurs between the lone-pair orbital of the carbonyl-O and the in-plane alkyne π* orbital. Depending on the type of catalyst (and its ligand), either a 5-exo-dig or 6-endo-dig cyclization will proceed. As highlighted in Scheme S1, the resultant by-product **46** was purified from a model substrate **1q**. Differentiation between the exo and endo forms can be made by NMR analysis, where protons at the C-1 position of **46** are expected to be down-shifted compared to the substrate.

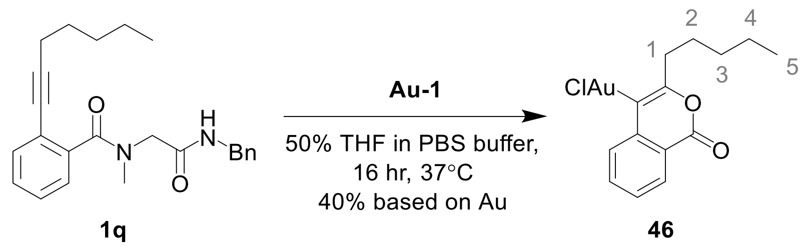

Scheme S1. Identification of the by-product **46**

1.5 Synthetic Schemes

1.5.1 Reactivity Study Substrates

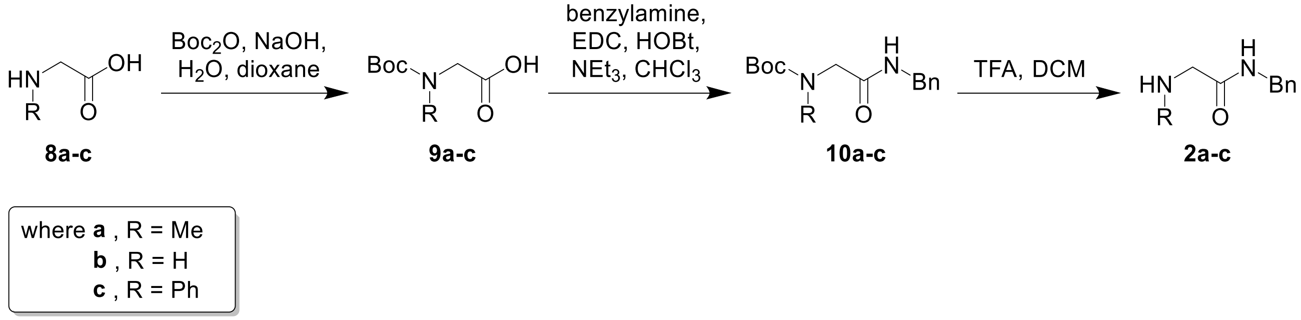

Scheme S2. Synthesis of **2a-c**

Preparation of **9a** :

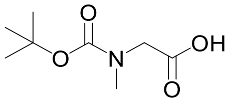

N-methylglycine **8a** (593 mg, 6.66 mmol) was dissolved in dioxane (10 ml) and H_2O (6 ml). NaOH (1.2 g, 30 mmol) dissolved in H_2O (2 ml) was added and the mixture was cooled to 0°C. Boc anhydride (1.73 g, 7.92 mmol) dissolved in dioxane (2 ml) was then added dropwise and the mixture was stirred for 16 hr at room temperature. To workup, dioxane in the mixture was evaporated under vacuum and the residual solution was washed with EtOAc. The aqueous layer was then acidified to pH~3 with 1N HCl, which allowed the product to be extracted with $CHCl_3$.The combined organic layer was washed with H_2O, brine, dried over sodium sulfate, and evaporated under vacuum to give the desired pure compound. Yield: 1250 mg, quan. Characterization matched a previous report of this known compound.[1] **^{1}H NMR** (DMSO-d6, 300 MHz) δ 12.58 (br s, 1H), 3.83 (d, *J* = 5.2 Hz, 2H), 2.80 (d, *J* = 10.8 Hz, 3H), 1.36 (d, *J* = 15.1 Hz, 9H).

Preparation of **9c** :

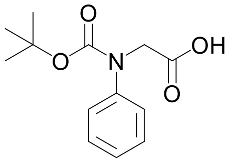

N-Phenylglycine **8c** (500 mg, 3.31 mmol) was dissolved in 1:1 acetone/H_2O (6ml). Boc anhydride (2.16 g, 9.89 mmol) dissolved in acetone (1 ml) was then added dropwise and the mixture was stirred for 16 hr at room temperature. To workup, acetone in the mixture was evaporated under vacuum and the residual solution was washed with ether. The aqueous layer was then acidified to pH~3 with 1N HCl, which allowed the product to be extracted with $CHCl_3$.The combined organic layer was washed with H_2O, brine, dried over sodium sulfate, and evaporated under vacuum. Flash column chromatography using a gradient of 0-10% MeOH in $CHCl_3$ was used to purify the desired compound. Yield: 804 mg, 97%. R_f =0.29 (MeOH/$CHCl_3$, 1:10). Characterization matched a previous report of this known compound.[2] **^{1}H NMR** (DMSO-d6, 400 MHz) δ 12.78 (br s, 1H), 7.34-7.15 (m, 5H), 4.19 (s, 2H), 1.36 (s, 9H).

Preparation of **10a** :

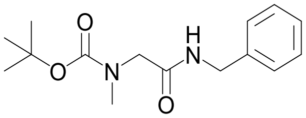

Compound **9a** (951 mg, 5.03 mmol), benzylamine (550 μl, 5.03 mmol), EDC (1558 mg, 10.06 mmol), HOBt (1357 mg, 10.06 mmol) were dissolved in $CHCl_3$ (10 ml). NEt_3 (1.4 ml, 10.06 mmol) was then added and the mixture was stirred for 16 hr at room temperature under N_2. To workup, the solvent was evaporated under vacuum and the mixture redissolved in EtOAc. The organic layer was then washed with H_2O, brine, dried over sodium sulfate, and evaporated under vacuum. Flash column chromatography using a gradient of 10-50% EtOAc in Hex was used to purify the desired compound. Yield: 1293 mg, 92%. R_f =0.26 (EtOAc/Hex, 1:1). **^{1}H NMR** ($CDCl_3$, 300 MHz) δ 7.35-7.23 (m, 5H), 4.46 (d, *J* = 5.9 Hz, 2H), 3.89 (s, 2H), 2.93 (s, 3H), 1.40 (s, 9H); **^{13}C NMR** ($CDCl_3$, 75 MHz): δ 169.6, 155.6, 138.2, 128.9, 127.8, 127.7, 80.9, 53.4, 43.3, 35.9, 28.3. **HRMS** for $C_{15}H_{22}N_2O_3$ $[M+Na]^+$ calcd. 301.1523 found 301.1517.

Preparation of **10b** :

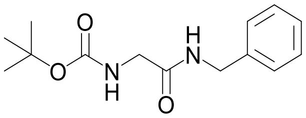

N-Boc-glycine **9b** (100 mg, 0.571 mmol), benzylamine (63 μl, 0.571 mmol), EDC (177 mg, 1.14 mmol), HOBt (154 mg, 1.14 mmol) were dissolved in $CHCl_3$ (10 ml). NEt_3 (160 μl, 1.14 mmol) was then added and the mixture was stirred for 16 hr at room temperature under N_2. To workup, the solvent was evaporated under vacuum and the mixture redissolved in EtOAc. The organic layer was then washed with H_2O, brine, dried over sodium sulfate, and evaporated under vacuum. Flash column chromatography using a gradient of 10-50% EtOAc in Hex was used to purify the desired compound. Yield: 538 mg, 89%. R_f =0.21 (EtOAc/Hex, 1:1). **^{1}H NMR** ($CDCl_3$, 300 MHz) δ 7.36-7.24 (m, 5H), 6.51 (br s, 1H), 5.19 (br s, 1H), 4.46 (d, *J* = 5.6 Hz, 2H), 3.82 (d, *J* = 5.9 Hz, 2H), 1.42 (s, 9H); **^{13}C NMR** ($CDCl_3$, 75 MHz): δ 169.6, 156.3, 138.1, 128.9, 127.9, 127.8, 80.6, 44.7, 43.5, 28.3. **HRMS** for $C_{14}H_{20}N_2O_3$ $[M+Na]^+$ calcd. 287.1366, found 287.1369.

Preparation of **10c** :

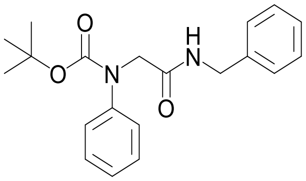

Compound **9c** (830 mg, 3.31 mmol), benzylamine (362 μl, 3.31 mmol), EDC (1025 mg, 6.61 mmol), HOBt (893 mg, 6.61 mmol) were dissolved in $CHCl_3$ (10 ml). NEt_3 (924 μl, 6.61 mmol) was then added and the mixture was stirred for 16 hr at room temperature under N_2. To workup, the solvent was evaporated under vacuum and the mixture redissolved in EtOAc. The organic layer was then washed with H_2O, brine, dried over sodium sulfate, and evaporated under vacuum. Flash column chromatography using a gradient of 10-50% EtOAc in Hex was used to purify the desired compound. Yield: 994 mg, 88%. R_f =0.53 (EtOAc/Hex, 1:1). **^{1}H NMR** ($CDCl_3$, 300 MHz) δ 7.36-7.18 (m, 10H), 6.52 (br s, 1H), 4.50 (d, *J* = 5.8 Hz, 2H), 4.29 (s, 2H), 1.39 (s, 9H); **^{13}C NMR** ($CDCl_3$, 75 MHz): δ 169.5, 155.0, 142.7, 138.2, 129.2, 128.9, 127.9, 127.8, 126.5, 125.9, 81.9, 54.6, 43.6, 28.2. **HRMS** for $C_{20}H_{24}N_2O_3$ $[M+Na]^+$ calcd. 363.1679, found 363.1683.

Preparation of **2a** :

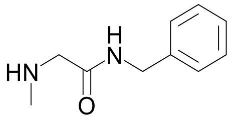

Compound **10a** (1293 mg, 4.65 mmol) was dissolved in a mixture of TFA:DCM (1 ml:1 ml) and stirred for 60 min at room temperature. Completion of the reaction was determined by disappearance of starting material spot on TLC (100% EtOAc). To workup, the reaction was evaporated under vacuum to an oil. Flash column chromatography using a gradient of 0-10% MeOH in $CHCl_3$ was used to purify the desired compound. Yield: 828 mg, quan. R_f =0.12 (MeOH/$CHCl_3$, 1:10). ^{1}**H NMR** (CD_3OD, 300 MHz) δ 7.36-7.23 (m, 5H), 4.43 (s, 2H), 3.82 (s, 2H), 2.73 (s, 3H); ^{13}**C NMR** (CD_3OD, 75 MHz): δ 166.5, 139.4, 129.8, 128.8, 128.6, 50.6, 44.2, 33.5. **HRMS** for $C_{10}H_{14}N_2O$ $[M+H]^+$ calcd. 179.1179, found 179.1182.

Preparation of **2b** :

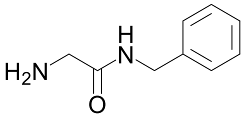

Compound **10b** (235 mg, 0.893 mmol) was dissolved in a mixture of TFA:DCM (1 ml:1 ml) and stirred for 60 min at room temperature. Completion of the reaction was determined by disappearance of starting material spot on TLC (MeOH/$CHCl_3$, 1:10). To workup, the reaction was evaporated under vacuum to an oil. Flash column chromatography using a gradient of 0-10% MeOH in $CHCl_3$ was used to purify the desired compound. Yield: 146 mg, quan. R_f =0.03 (MeOH/$CHCl_3$, 1:10). ^{1}**H NMR** (CD_3OD, 300 MHz) δ 7.32-7.18 (m, 5H), 4.39 (s, 2H), 3.68 (s, 2H); ^{13}**C NMR** (CD_3OD, 75 MHz): δ 167.4, 139.5, 129.7, 128.8, 128.6, 44.2, 41.5. **HRMS** for $C_9H_{12}N_2O$ $[M+Na]^+$ calcd. 187.0842, found 187.0846.

Preparation of **2c** :

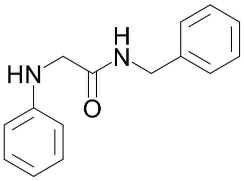

Compound **10c** (225 mg, 0.661 mmol) was dissolved in a mixture of TFA:DCM (1 ml:1 ml) and stirred for 60 min at room temperature. Completion of the reaction was determined by disappearance of starting material spot on TLC (MeOH/$CHCl_3$, 1:10). To workup, the reaction was evaporated under vacuum to an oil. Flash column chromatography using a gradient of 0-5% MeOH in $CHCl_3$ was used to purify the desired compound. Yield: 158 mg, quan. R_f =0.33 (MeOH/$CHCl_3$, 1:10). ^{1}**H NMR** ($CDCl_3$, 300 MHz) δ 7.57 (br s, 2H), 7.43 (br s, 1H), 7.31-7.17 (m, 6H), 6.94 (t, J = 7.4 Hz, 1H), 6.77 (d, J = 8.3 Hz, 2H), 4.44 (d, J = 6.0 Hz, 2H), 3.91 (s, 2H). ^{13}**C NMR** ($CDCl_3$, 75 MHz): δ 170.9, 145.1, 137.5, 129.8, 128.9, 127.8(2), 121.4, 115.1, 49.5, 43.5. **HRMS** for $C_{15}H_{16}N_2O$ $[M+H]^+$ calcd. 241.1335, found 241.1339.

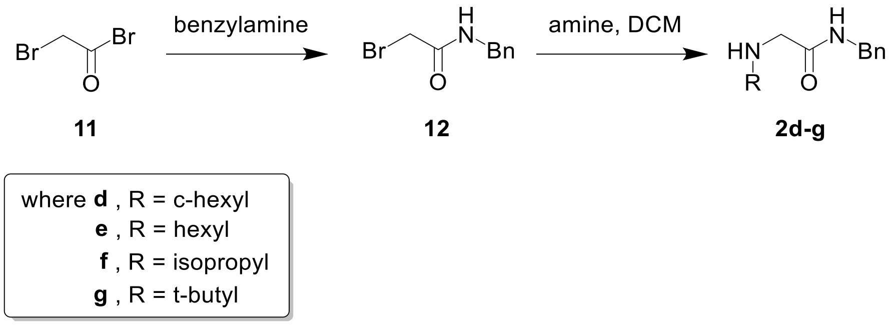

Scheme S3. Synthesis of **2d-g**

Preparation of **12** :

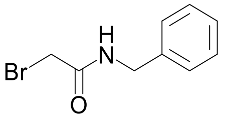

Bromoacetyl bromide **11** (120 µl, 1.38 mmol) was dissolved in DCM (3 ml) and K_2CO_3 (506 mg, 3.66 mmol) was dissolved in water (3 ml). Both mixtures were added simultaneously dropwise over 15 min to a solution of benzylamine (100 µl, 0.92 mmol) in DCM (3 ml) at 0°C. The reaction was then stirred at room temperature for 2 hr. To workup, the reaction was diluted with H_2O and then the organic layer was washed with H_2O (5×), dried over magnesium sulfate, and evaporated under vacuum to give the desired pure compound. Yield: 200 mg, 96%. R_f =0.42 (100% DCM). Characterization matched a previous report of this known compound.[3] **^{1}H NMR** (CD_2Cl_2, 300 MHz) δ 7.39-7.26 (m, 5H), 6.73 (br s, 1H), 4.45 (d, *J* = 5.9 Hz, 2H), 3.91 (s, 2H).

Preparation of **2d** :

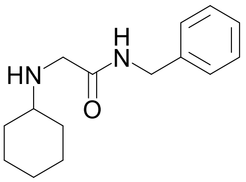

Compound **12** (200 mg, 0.915 mmol) was dissolved in DCM (2 ml). Cyclohexylamine (316 µl, 2.75 mmol) was then added and the mixture was stirred for 16 hr and at room temperature under N_2. To workup, the reaction was evaporated under vacuum to an oil. Flash column chromatography using a gradient of 0-5% MeOH in $CHCl_3$ was used to purify the desired compound. Yield: 203 mg, 90%. R_f =0.27 (MeOH/$CHCl_3$, 1:10). **^{1}H NMR** (CD_3OD, 300 MHz) δ 7.36-7.25 (m, 5H), 4.42 (s, 2H), 3.39 (s, 2H), 2.55-2.46 (m, 1H), 1.93-1.64 (m, 4H), 1.36-1.07 (m, 6H); **^{13}C NMR** (CD_3OD, 75 MHz): δ 173.2, 139.9, 129.7, 128.8, 128.5, 58.1, 44.0, 33.4, 26.9, 25.9, 18.3. **HRMS** for $C_{15}H_{22}N_2O$ $[M+H]^+$ calcd. 247.1805, found 247.1805.

Preparation of **2e** :

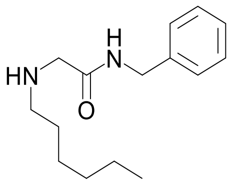

Compound **12** (209 mg, 0.920 mmol) was dissolved in DCM (2 ml). n-Hexylamine (1.2 ml, 9.920 mmol) was then added and the mixture was stirred for 16 hr and at room temperature under N_2. To workup, the reaction was evaporated under vacuum to an oil. Flash column chromatography using a gradient of 0-5% MeOH in $CHCl_3$ was used to purify the desired compound. Yield: 194 mg, 85%. R_f =0.39 (MeOH/$CHCl_3$, 1:10). **^{1}H NMR** (CD_3OD, 300 MHz) δ 7.36-7.23 (m, 5H), 4.42 (s, 2H), 3.27 (s, 2H), 2.55 (t, *J* = 7.3 Hz, 2H), 1.54-1.44 (m, 2H), 1.37-1.26 (m, 6H), 0.91 (t, *J* = 7.0 Hz, 3H); **^{13}C NMR** (CD_3OD, 75 MHz): δ 174.1, 140.0, 129.7, 128.7, 128.4, 52.8, 50.6, 43.9, 32.8, 30.6, 28.0, 23.6, 14.3. **HRMS** for $C_{15}H_{24}N_2O$ $[M+H]^+$ calcd. 249.1961, found 249.1959.

Preparation of **2f** :

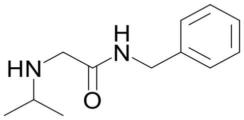

Compound **12** (180 mg, 0.791 mmol) was dissolved in DCM (2 ml). 2-Propylamine (339 μl, 3.96 mmol) was then added and the mixture was stirred for 16 hr and at room temperature under N_2. To workup, the reaction was evaporated under vacuum to an oil. Flash column chromatography using a gradient of 0-5% MeOH in $CHCl_3$ was used to purify the desired compound. Yield: 139 mg, 85%. R_f =0.28 (MeOH/$CHCl_3$, 1:10). **^{1}H NMR** (CD_3OD, 300 MHz) δ 7.37-7.22 (m, 5H), 4.41 (s, 2H), 3.29 (s, 2H), 2.78 (quin, J = 6.2 Hz, 1H), 1.09-1.05 (m, 6H); **^{13}C NMR** (CD_3OD, 75 MHz): δ 174.2, 139.9, 129.7, 128.7, 128.4, 50.4, 43.9, 22.6, 18.3. **HRMS** for $C_{12}H_{18}N_2O$ $[M+H]^+$ calcd. 207.1492, found 207.1496.

Preparation of **2g** :

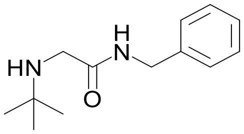

Compound **12** (216 mg, 0.952 mmol) was dissolved in DCM (2 ml). 2-Methyl-2-propylamine (486 μl, 4.58 mmol) was then added and the mixture was stirred for 16 hr and at room temperature under N_2. To workup, the reaction was evaporated under vacuum to an oil. Flash column chromatography using a gradient of 0-5% MeOH in $CHCl_3$ was used to purify the desired compound. Yield: 154 mg, 74%. R_f =0.27 (MeOH/$CHCl_3$, 1:10). **^{1}H NMR** (CD_3OD, 300 MHz) δ 7.35-7.22 (m, 5H), 4.41 (s, 2H), 3.26 (s, 2H), 1.09 (s, 9H); **^{13}C NMR** (CD_3OD, 75 MHz): δ 175.0, 139.9, 129.7, 128.7, 128.4, 51.5, 46.4, 43.9, 28.8. **HRMS** for $C_{13}H_{20}N_2O$ $[M+H]^+$ calcd. 221.1648, found 221.1648.

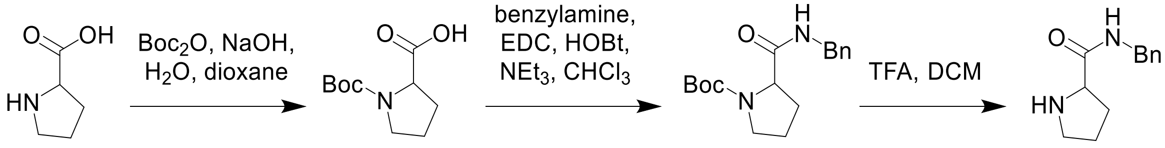

8h **9h** **10h** **2h**

Scheme S4. Synthesis of **2h**

Preparation of **9h** :

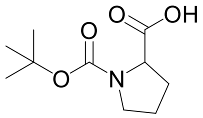

L-Proline **8h** (766 mg, 6.66 mmol) was dissolved in dioxane (6 ml) and H_2O (10 ml). NaOH (1.2 g, 30 mmol) dissolved in H_2O (2 ml) was added and the mixture was cooled to 0°C. Boc anhydride (1.73 g, 7.92 mmol) dissolved in dioxane (2 ml) was then added dropwise and the mixture was stirred for 16 hr at room temperature. To workup, dioxane in the mixture was evaporated under vacuum and the residual solution was washed with EtOAc. The aqueous layer was then acidified to pH~3 with 1N HCl, which allowed the product to be extracted with $CHCl_3$.The combined organic layer was washed with H_2O, brine, dried over sodium sulfate, and evaporated under vacuum to give the desired compound. Yield: 1220 mg, 85%. Characterization matched a previous report of this known compound.[4] **^{1}H NMR** ($CDCl_3$, 300 MHz) δ 8.39 (br s, 1H), 4.44-4.21 (m, 1H), 3.61-3.32 (m, 2H), 2.41-1.84 (m, 4H), 1.59-1.35 (m, 9H).

Preparation of **10h** :

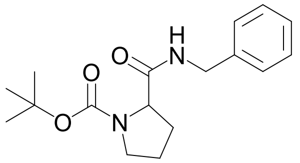

Compound **9h** (411 mg, 1.91 mmol), benzylamine (210 µl, 1.91 mmol), EDC (592 mg, 3.82 mmol), HOBt (516 mg, 3.82 mmol) were dissolved in $CHCl_3$ (10 ml). NEt_3 (534 µl, 3.82 mmol) was then added and the mixture was stirred for 16 hr at room temperature under N_2. To workup, the solvent was evaporated under vacuum and the mixture redissolved in EtOAc. The organic layer was then washed with H_2O, brine, dried over sodium sulfate, and evaporated under vacuum. Flash column chromatography using a gradient of 25-50% EtOAc in Hex was used to purify the desired compound. Yield: 543 mg, 93%. R_f =0.16 (EtOAc/Hex, 1:1). **^{1}H NMR** ($CDCl_3$, 300 MHz) δ 7.37-7.18 (m, 5H), 6.39 (br s, 1H), 4.63-4.20 (m, 3H), 3.41 (s, 2H), 2.42-1.76 (m, 4H), 1.39 (s, 9H). **^{13}C NMR** ($CDCl_3$, 75 MHz): δ 172.6, 138.4, 128.8, 127.9, 127.5, 80.7, 60.1, 47.2, 43.4, 31.3, 28.3, 24.6, 7.3. **HRMS** for $C_{17}H_{24}N_2O_3$ $[M+Na]^+$ calcd. 327.1679, found 327.1683.

Preparation of **2h** :

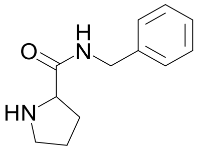

Compound **10h** (875 mg, 2.88 mmol) was dissolved in a mixture of TFA:DCM (1 ml:1 ml) and stirred for 60 min at room temperature. Completion of the reaction was determined by disappearance of starting material spot on TLC (EtOAc/Hex, 1:1). To workup, the reaction was evaporated under vacuum to an oil. Flash column chromatography using a gradient of 0-10% MeOH in $CHCl_3$ was used to purify the desired compound. Yield: 588 mg, quan. R_f =0.18 (100% EtOAc). **^{1}H NMR** (CD_3OD, 300 MHz) δ 7.30-7.18 (m, 5H), 4.38 (s, 2H), 4.24-4.19 (m, 1H), 3.35-3.23 (m, 2H), 2.42-2.32 (m, 1H), 2.04-1.89 (m, 3H). **^{13}C NMR** (CD_3OD, 75 MHz): δ 169.9, 139.5, 129.8, 128.8, 128.6, 61.2, 47.3, 44.4, 31.0, 25.1. **HRMS** for $C_{12}H_{16}N_2O$ $[M+H]^+$ calcd. 205.1335, found 205.1339.

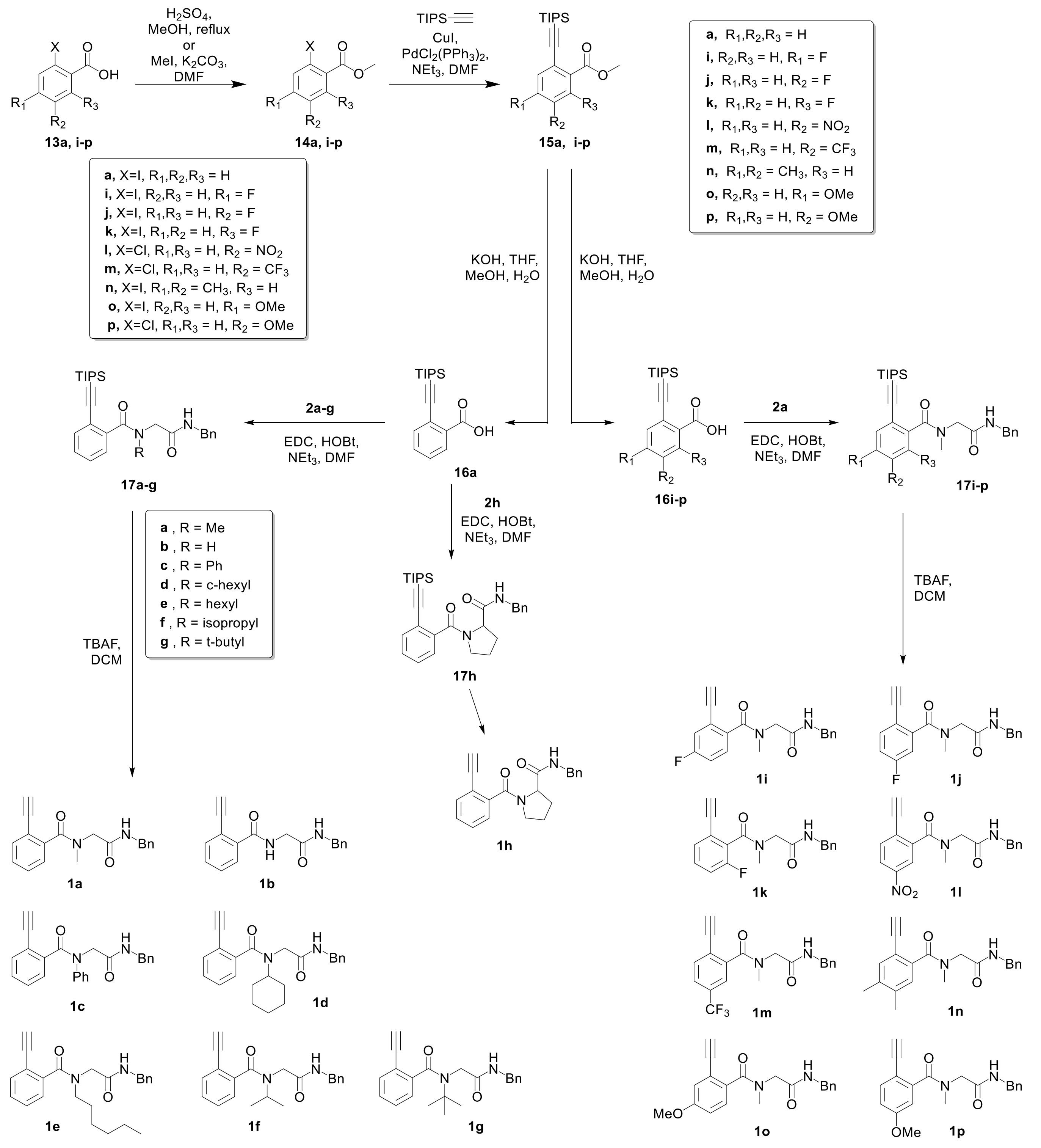

Scheme S5. Synthesis of **1a-p**

Preparation of **14a** :

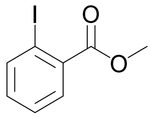

2-Iodobenzoic acid **13a** (1 g, 4.03 mmol) was dissolved in MeOH (20 ml). Sulfuric acid (2.7 ml) was then slowly added and the mixture was stirred for 16 hr and heated at reflux under N_2. To workup, the reaction was cooled to room temperature, before diluting with ether. The organic layer was then washed with H_2O, brine, dried over magnesium sulfate, and evaporated under vacuum to give the desired pure compound. Yield: 847 mg, 80%. Characterization matched a previous report of this known compound.[5] **^{1}H NMR** ($CDCl_3$, 300 MHz) δ 8.00 (dd, *J* = 1.0, 7.9 Hz, 1H), 7.80 (dd, *J* = 1.7, 7.8 Hz, 1H), 7.40 (td, *J* = 1.1, 7.6 Hz, 1H), 7.15 (td, *J* = 1.7, 7.7 Hz, 1H), 3.93 (s, 3H).

Preparation of **14i** :

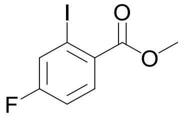

4-Fluoro-2-iodobenzoic acid **13i** (300 mg, 1.13 mmol) and K_2CO_3 (234 mg, 1.69 mmol) were dissolved in DMF (10 ml) and stirred for 5 min. CH_3I (105 μl, 1.69 mmol) was then added and the mixture was stirred for 16 hr at room temperature under N_2. To workup, the reaction was diluted with EtOAc and then the organic layer was washed with H_2O, brine, dried over sodium sulfate, and evaporated under vacuum to give the desired pure compound. Yield: 300 mg, quan. R_f =0.58 (EtOAc/Hex, 1:4). Characterization matched a previous report of this known compound.[6] **^{1}H NMR** ($CDCl_3$, 300 MHz) δ 7.86 (dd, *J* = 5.9, 8.8 Hz, 1H), 7.72 (dd, *J* = 2.5, 8.2 Hz, 1H), 7.11 (td, *J* = 2.6, 8.7 Hz, 1H), 3.91 (s, 3H).

Preparation of **14j** :

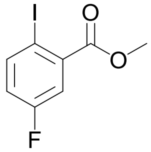

5-Fluoro-2-iodobenzoic acid **13j** (300 mg, 1.13 mmol) and K_2CO_3 (234 mg, 1.69 mmol) were dissolved in DMF (10 ml) and stirred for 5 min. CH_3I (105 μl, 1.69 mmol) was then added and the mixture was stirred for 16 hr at room temperature under N_2. To workup, the reaction was diluted with EtOAc and then the organic layer was washed with H_2O, brine, dried over sodium sulfate, and evaporated under vacuum to give the desired pure compound. Yield: 300 mg, quan. R_f =0.60 (EtOAc/Hex, 1:4). Characterization matched a previous report of this known compound.[7] **^{1}H NMR** ($CDCl_3$, 300 MHz) δ 7.94 (dd, *J* = 5.4, 8.7 Hz, 1H), 7.54 (dd, *J* = 3.0, 9.0 Hz, 1H), 6.94 (td, *J* = 3.0, 7.8 Hz, 1H), 3.94 (s, 3H).

Preparation of **14k** :

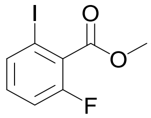

6-Fluoro-2-iodobenzoic acid **13k** (300 mg, 1.13 mmol) and K_2CO_3 (234 mg, 1.69 mmol) were dissolved in DMF (10 ml) and stirred for 5 min. CH_3I (105 μl, 1.69 mmol) was then added and the mixture was stirred for 16 hr at room temperature under N_2. To workup, the reaction was diluted with EtOAc and then the organic layer was washed with H_2O, brine, dried over sodium sulfate, and evaporated under vacuum to give the desired pure compound. Yield: 299 mg, quan. R_f =0.59 (EtOAc/Hex, 1:4). Characterization matched a previous report of this known compound.[8] **^{1}H NMR** ($CDCl_3$, 300 MHz) δ 7.68-7.61 (m, 1H), 7.13-7.09 (m, 2H), 3.98 (s, 3H).

Preparation of **14l** :

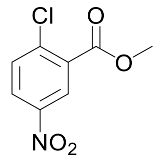

5-Nitro-2-chlorobenzoic acid **13l** (216 mg, 1.07 mmol) and K_2CO_3 (222 mg, 1.61 mmol) were dissolved in DMF (10 ml) and stirred for 5 min. CH_3I (100 µl, 1.61 mmol) was then added and the mixture was stirred for 16 hr at room temperature under N_2. To workup, the reaction was diluted with EtOAc and then the organic layer was washed with H_2O, brine, dried over sodium sulfate, and evaporated under vacuum to give the desired pure compound. Yield: 230 mg, quan. R_f =0.58 (EtOAc/Hex, 1:4). Characterization matched a previous report of this known compound.[9] ^{1}**H NMR** ($CDCl_3$, 300 MHz) δ 8.74-8.72 (m, 1H), 8.31-8.26 (m, 1H), 7.67 (dd, *J* = 3.5, 8.8 Hz, 1H), 4.00 (s, 3H).

Preparation of **14m** :

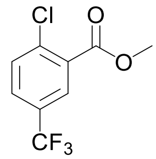

5-trifluoromethyl-2-chlorobenzoic acid **13m** (295 mg, 1.31 mmol) and K_2CO_3 (272 mg, 1.97 mmol) were dissolved in DMF (10 ml) and stirred for 5 min. CH_3I (123 µl, 1.97 mmol) was then added and the mixture was stirred for 16 hr at room temperature under N_2. To workup, the reaction was diluted with EtOAc and then the organic layer was washed with H_2O, brine, dried over sodium sulfate, and evaporated under vacuum to give the desired pure compound. Yield: 313 mg, quan. R_f =0.67 (EtOAc/Hex, 1:10). Characterization matched a previous report of this known compound.[10] ^{1}**H NMR** ($CDCl_3$, 300 MHz) δ 8.11 (s, 1H), 7.69-7.58 (m, 2H), 3.97 (s, 3H).

Preparation of **14n** :

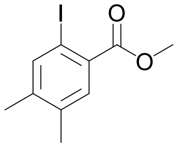

4,5-Dimethyl-2-iodobenzoic acid **13n** (300 mg, 1.09 mmol) and K_2CO_3 (225 mg, 1.63 mmol) were dissolved in DMF (10 ml) and stirred for 5 min. CH_3I (101 µl, 1.63 mmol) was then added and the mixture was stirred for 16 hr at room temperature under N_2. To workup, the reaction was diluted with EtOAc and then the organic layer was washed with H_2O, brine, dried over sodium sulfate, and evaporated under vacuum to give the desired pure compound. Yield: 299 mg, quan. R_f =0.60 (EtOAc/Hex, 1:4). Characterization matched a previous report of this known compound.[11] ^{1}**H NMR** ($CDCl_3$, 300 MHz) δ 7.77 (s, 1H), 7.62 (s, 1H), 3.91 (s, 3H), 2.23 (s, 6H).

Preparation of **14o** :

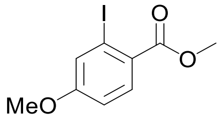

4-Methoxy-2-iodobenzoic acid **13o** (112 mg, 0.403 mmol) was dissolved in MeOH (2 ml). Sulfuric acid (270 µl) was then slowly added and the mixture was stirred for 16 hr and heated at reflux under N_2. To workup, the reaction was cooled to room temperature, before diluting with ether. The organic layer was then washed with H_2O, brine, dried over magnesium sulfate, and evaporated under vacuum to give the desired pure compound. Yield: 111 mg, quan. R_f =0.25 (EtOAc/Hex, 1:10). ^{1}**H NMR** ($CDCl_3$, 300 MHz) δ 7.89-7.84 (m, 1H), 7.55-7.52 (m, 1H), 6.94-6.88 (m, 1H), 3.90 (s, 3H), 3.84 (s, 3H); ^{13}**C NMR** ($CDCl_3$, 75 MHz): δ 166.3, 162.3, 132.8, 127.2, 126.3, 113.8, 95.8, 55.7, 52.3. **HRMS** for $C_9H_9IO_3$ $[M+H]^+$ calcd. 292.9669, found 292.9673.

Preparation of **14p** :

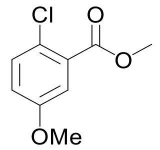

5-Methoxy-2-chlorobenzoic acid **13p** (285 mg, 1.53 mmol) and K_2CO_3 (317 mg, 2.29 mmol) were dissolved in DMF (10 ml) and stirred for 5 min. CH_3I (143 µl, 2.29 mmol) was then added and the mixture was stirred for 16 hr at room temperature under N_2. To workup, the reaction was diluted with EtOAc and then the organic layer was washed with H_2O, brine, dried over sodium sulfate, and evaporated under vacuum to give the desired pure compound. Yield: 306 mg, quan. R_f =0.26 (EtOAc/Hex, 1:10). Characterization matched a previous report of this known compound.[12] **^{1}H NMR** ($CDCl_3$, 300 MHz) δ 7.35-7.32 (m, 2H), 6.96 (dd, *J* = 2.9, 8.8 Hz, 1H), 3.93 (s, 3H), 3.82 (s, 3H).

Preparation of **15a** :

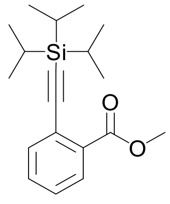

Compound **14a** (400 mg, 1.53 mmol), CuI (58 mg, 0.31 mmol), and $PdCl_2(PPh_3)_2$ (107 mg, 0.15 mmol) were dissolved in DMF (2 ml) and NEt_3 (2 ml). The mixture was degassed with N_2 for 5 min. (Triisopropylsilyl)acetylene (680 µl, 3.05 mmol) was then added and the mixture was stirred for 16 hr at 30°C under N_2. To workup, the reaction was diluted with EtOAc and then the organic layer was washed with H_2O, brine, dried over sodium sulfate, and evaporated under vacuum. Flash column chromatography using a gradient of 0-5% EtOAc in Hex was used to purify the desired compound. Yield: 474 mg, 98%. R_f =0.70 (EtOAc/Hex, 1:5). **^{1}H NMR** ($CDCl_3$, 300 MHz) δ 7.89 (dd, *J* = 1.3, 7.6 Hz, 1H), 7.60 (dd, *J* = 1.2, 7.8 Hz, 1H), 7.44 (td, *J* = 1.4, 7.5 Hz, 1H), 7.36 (td, *J* = 1.4, 7.7 Hz, 1H), 3.91 (s, 3H), 1.15 (s, 21H); **^{13}C NMR** ($CDCl_3$, 75 MHz): δ 167.5, 135.2, 132.8, 131.6, 130.4, 128.2, 123.6, 105.3, 96.5, 52.3, 18.7, 11.4. **HRMS** for $C_{19}H_{28}O_2Si$ $[M+H]^+$ calcd. 317.1931, found 317.1935.

Preparation of **15i** :

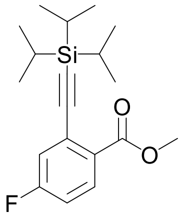

Compound **14i** (303 mg, 1.08 mmol), CuI (41 mg, 0.217 mmol), and $PdCl_2(PPh_3)_2$ (76 mg, 0.108 mmol) were dissolved in DMF (2 ml) and NEt_3 (2 ml). The mixture was degassed with N_2 for 5 min. (Triisopropylsilyl)acetylene (482 µl, 2.17 mmol) was then added and the mixture was stirred for 16 hr at 30°C under N_2. To workup, the reaction was diluted with EtOAc and then the organic layer was washed with H_2O, brine, dried over sodium sulfate, and evaporated under vacuum. Flash column chromatography using a gradient of 0-5% EtOAc in Hex was used to purify the desired compound. Yield: 350 mg, 96%. R_f =0.34 (EtOAc/Hex, 1:10). **^{1}H NMR** ($CDCl_3$, 300 MHz) δ 7.94 (dd, *J* = 5.8, 8.8 Hz, 1H), 7.28-7.25 (m, 2H), 7.06 (td, *J* = 2.6, 7.8 Hz, 1H), 3.90 (s, 3H), 1.15 (s, 21H); **^{13}C NMR** ($CDCl_3$, 75 MHz): δ 166.4, 166.0, 162.7, 133.2, 133.1, 121.9, 121.6, 115.9, 115.6, 104.0, 98.3, 52.3, 18.7, 11.3. **HRMS** for $C_{19}H_{27}FO_2Si$ $[M+H]^+$ calcd. 335.1837, found 335.1841.

Preparation of **15j** :

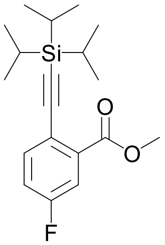

Compound **14j** (303 mg, 1.08 mmol), CuI (41 mg, 0.217 mmol), and $PdCl_2(PPh_3)_2$ (76 mg, 0.108 mmol) were dissolved in DMF (2 ml) and NEt_3 (2 ml). The mixture was degassed with N_2 for 5 min. (Triisopropylsilyl)acetylene (482 µl, 2.17 mmol) was then added and the mixture was stirred for 16 hr at 30°C under N_2. To workup, the reaction was diluted with EtOAc and then the organic layer was washed with H_2O, brine, dried over sodium sulfate, and evaporated under vacuum. Flash column chromatography using a gradient of 0-5% EtOAc in Hex was used to purify the desired compound. Yield: 318 mg, 88%. R_f =0.44 (EtOAc/Hex, 1:10). **^{1}H NMR** ($CDCl_3$, 300 MHz) δ 7.61-7.56 (m, 2H), 7.18-7.12 (m, 1H), 3.92 (s, 3H), 1.14 (s, 21H); **^{13}C NMR** ($CDCl_3$, 75 MHz): δ 166.2, 163.6, 160.3, 137.2, 137.1, 119.2, 118.9, 117.6, 117.3, 104.2, 96.3, 52.6, 18.7, 11.4. **HRMS** for $C_{19}H_{27}FO_2Si$ $[M+H]^+$ calcd. 335.1837, found 335.1840.

Preparation of **15k** :

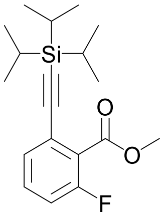

Compound **14k** (303 mg, 1.08 mmol), CuI (41 mg, 0.217 mmol), and $PdCl_2(PPh_3)_2$ (76 mg, 0.108 mmol) were dissolved in DMF (2 ml) and NEt_3 (2 ml). The mixture was degassed with N_2 for 5 min. (Triisopropylsilyl)acetylene (482 µl, 2.17 mmol) was then added and the mixture was stirred for 16 hr at 30°C under N_2. To workup, the reaction was diluted with EtOAc and then the organic layer was washed with H_2O, brine, dried over sodium sulfate, and evaporated under vacuum. Flash column chromatography using a gradient of 0-5% EtOAc in Hex was used to purify the desired compound. Yield: 320 mg, 89%. R_f =0.38 (EtOAc/Hex, 1:10). **^{1}H NMR** ($CDCl_3$, 300 MHz) δ 7.37-7.29 (m, 2H), 7.12-7.03 (m, 1H), 3.93 (s, 3H), 1.12 (s, 21H); **^{13}C NMR** ($CDCl_3$, 75 MHz): δ 165.1, 161.2, 157.9, 131.4, 131.3, 129.2(2), 116.4, 116.1, 102.8, 96.7, 52.8, 18.6, 11.2. **HRMS** for $C_{19}H_{27}FO_2Si$ $[M+H]^+$ calcd. 335.1837, found 335.1841.

Preparation of **15l** :

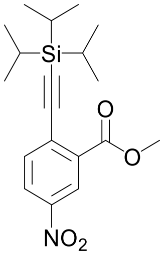

Compound **14l** (200 mg, 0.930 mmol), CuI (35 mg, 0.186 mmol), and $PdCl_2(PPh_3)_2$ (65 mg, 0.093 mmol) were dissolved in DMF (2 ml) and NEt_3 (2 ml). The mixture was degassed with N_2 for 5 min. (Triisopropylsilyl)acetylene (414 µl, 1.86 mmol) was then added and the mixture was stirred for 16 hr at 30°C under N_2. To workup, the reaction was diluted with EtOAc and then the organic layer was washed with H_2O, brine, dried over sodium sulfate, and evaporated under vacuum. Flash column chromatography using a gradient of 0-5% EtOAc in Hex was used to purify the desired compound. Yield: 337 mg, 94%. R_f =0.0.37 (EtOAc/Hex, 1:10). **^{1}H NMR** ($CDCl_3$, 300 MHz) δ 8.76 (d, *J* = 2.3 Hz, 1H), 8.28 (dd, *J* = 2.5, 8.6 Hz, 1H), 7.75 (d, *J* = 8.5 Hz, 1H), 3.97 (s, 3H), 1.16 (s, 21H); **^{13}C NMR** ($CDCl_3$, 75 MHz): δ 165.1, 146.7, 136.2, 133.9, 130.0, 125.9, 125.8, 104.4, 103.4, 52.9, 18.7, 11.3. **HRMS** for $C_{19}H_{27}NO_4Si$ $[M+Na]^+$ calcd. 384.1602, found 384.1605.

Preparation of **15m** :

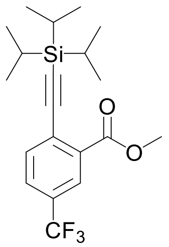

Compound **14m** (282 mg, 1.18 mmol), CuI (45 mg, 0.236 mmol), and $PdCl_2(PPh_3)_2$ (83 mg, 0.118 mmol) were dissolved in DMF (2 ml) and NEt_3 (2 ml). The mixture was degassed with N_2 for 5 min. (Triisopropylsilyl)acetylene (526 µl, 2.36 mmol) was then added and the mixture was stirred for 16 hr at 30°C under N_2. To workup, the reaction was diluted with EtOAc and then the organic layer was washed with H_2O, brine, dried over sodium sulfate, and evaporated under vacuum. Flash column chromatography using a gradient of 0-5% EtOAc in Hex was used to purify the desired compound. Yield: 148 mg, 33%. R_f =0.43 (EtOAc/Hex, 1:10). **^{1}H NMR** ($CDCl_3$, 300 MHz) δ 8.17 (s, 1H), 7.72-7.65 (m, 2H), 3.94 (s, 3H), 1.16 (s, 21H); **^{13}C NMR** ($CDCl_3$, 75 MHz): δ 166.1, 135.7, 133.3, 130.4, 129.9, 128.1, 128.0, 127.6, 127.5, 103.9, 100.4, 52.6, 18.7, 11.3. **HRMS** for $C_{20}H_{27}F_3O_2Si$ $[M+Na]^+$ calcd. 407.1625, found 407.1629.

Preparation of **15n** :

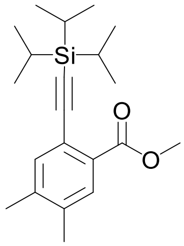

Compound **14n** (291 mg, 1.00 mmol), CuI (38 mg, 0.201 mmol), and $PdCl_2(PPh_3)_2$ (71 mg, 0.100 mmol) were dissolved in DMF (2 ml) and NEt_3 (2 ml). The mixture was degassed with N_2 for 5 min. (Triisopropylsilyl)acetylene (446 µl, 2.01 mmol) was then added and the mixture was stirred for 16 hr at 30°C under N_2. To workup, the reaction was diluted with EtOAc and then the organic layer was washed with H_2O, brine, dried over sodium sulfate, and evaporated under vacuum. Flash column chromatography using a gradient of 0-5% EtOAc in Hex was used to purify the desired compound. Yield: 337 mg, 97%. R_f =0.49 (EtOAc/Hex, 1:10). **^{1}H NMR** ($CDCl_3$, 300 MHz) δ 7.70 (s, 1H), 7.37 (s, 1H), 3.90 (s, 3H), 2.29 (s, 3H), 2.28 (s, 3H), 1.16 (s, 21H); **^{13}C NMR** ($CDCl_3$, 75 MHz): δ 167.6, 141.0, 137.3, 136.2, 131.7, 130.0, 121.0, 105.8, 94.8, 52.1, 19.6, 19.5, 18.7, 11.4. **HRMS** for $C_{21}H_{32}O_2Si$ $[M+H]^+$ calcd. 345.2244, found 345.2248.

Preparation of **15o** :

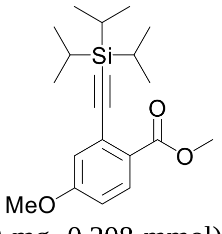

Compound **14o** (304 mg, 1.04 mmol), CuI (40 mg, 0.208 mmol), and $PdCl_2(PPh_3)_2$ (73 mg, 0.104 mmol) were dissolved in DMF (2 ml) and NEt_3 (2 ml). The mixture was degassed with N_2 for 5 min. (Triisopropylsilyl)acetylene (463 µl, 2.08 mmol) was then added and the mixture was stirred for 16 hr at 30°C under N_2. To workup, the reaction was diluted with EtOAc and then the organic layer was washed with H_2O, brine, dried over sodium sulfate, and evaporated under vacuum. Flash column chromatography using a gradient of 0-5% EtOAc in Hex was used to purify the desired compound. Yield: 321 mg, 89%. R_f =0.59 (EtOAc/Hex, 1:4). **^{1}H NMR** ($CDCl_3$, 300 MHz) δ 7.91 (d, J = 8.8 Hz, 1H), 7.07 (d, J = 2.7 Hz, 1H), 6.87 (dd, J = 2.7, 8.8 Hz, 1H), 3.88 (s, 3H), 3.85 (s, 3H), 1.16 (s, 21H); **^{13}C NMR** ($CDCl_3$, 75 MHz): δ 166.9, 162.0, 132.8, 125.6, 124.9, 120.0, 114.2, 105.4, 96.4, 55.6, 52.0, 18.7, 11.4. **HRMS** for $C_{20}H_{30}O_3Si$ $[M+Na]^+$ calcd. 369.1856, found 369.1860.

Preparation of **15p** :

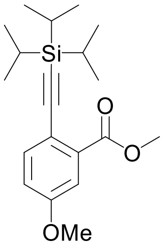

Compound **14p** (225 mg, 1.12 mmol), XPhos (107 mg, 0.224 mmol), $PdCl_2(CH_3CN)_2$ (29 mg, 0.112 mmol), and $CsCO_3$ (914 mg, 2.80 mmol) were dissolved in DMF (5 ml). The mixture was degassed with N_2 for 5 min. (Triisopropylsilyl)acetylene (500 µl, 2.24 mmol) was then added and the mixture was stirred for 16 hr at 90°C under N_2. To workup, the reaction was diluted with EtOAc and then the organic layer was washed with H_2O, brine, dried over sodium sulfate, and evaporated under vacuum. Flash column chromatography using a gradient of 0-5% EtOAc in Hex was used to purify the desired compound. Yield: 379 mg, 97%. R_f =0.63 (EtOAc/Hex, 1:4). **^{1}H NMR** ($CDCl_3$, 300 MHz) δ 7.53 (d, *J* = 8.6 Hz, 1H), 7.41 (d, *J* = 2.8 Hz, 1H), 6.99 (dd, *J* = 2.9, 8.6 Hz, 1H), 3.92 (s, 3H), 3.86 (s, 3H), 1.15 (s, 21H); **^{13}C NMR** ($CDCl_3$, 75 MHz): δ 167.4, 159.4, 136.6, 134.2, 118.3, 115.9, 114.8, 105.3, 94.2, 55.7, 52.3, 18.7, 11.4. **HRMS** for $C_{20}H_{30}O_3Si$ $[M+Na]^+$ calcd. 369.1856, found 369.1860.

Preparation of **16a** :

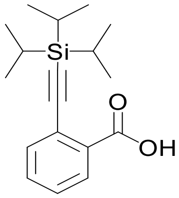

Compound **15a** (265 mg, 0.839 mmol) was dissolved in MeOH (3 ml) and THF (3 ml) at 0°C. KOH (471 mg, 8.39 mmol) dissolved in H_2O (3 ml) was then added and the mixture was stirred for 3 hr at rt under N_2. To workup, the mixture was acidified to pH~3 with 1M HCl solution, which allowed the product to be extracted with EtOAc. The organic layer was washed with H_2O, brine, dried over sodium sulfate, and evaporated under vacuum. Flash column chromatography using a gradient of 10-25% EtOAc in Hex was used to purify the desired compound. Yield: 265 mg, quan. R_f =0.73 (EtOAc/Hex, 1:1). **^{1}H NMR** ($CDCl_3$, 300 MHz) δ 8.07 (d, *J* = 7.8 Hz, 1H), 7.64 (d, *J* = 7.7 Hz, 1H), 7.51 (td, *J* = 1.3, 7.5 Hz, 1H), 7.41 (td, *J* = 1.4, 7.8 Hz, 1H), 1.16 (s, 21H); **^{13}C NMR** ($CDCl_3$, 75 MHz): δ 170.8, 135.2, 132.5, 131.5, 131.3, 128.4, 124.2, 104.8, 98.7, 18.7, 11.4; **HRMS** for $C_{18}H_{26}O_2Si$ $[M+H]^+$ calcd. 303.1775, found 303.1776.

Preparation of **16i** :

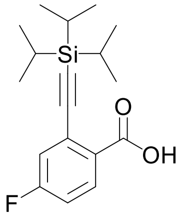

Compound **15i** (361 mg, 1.08 mmol) was dissolved in MeOH (6 ml) and THF (6 ml) at 0°C. KOH (607 mg, 10.83 mmol) dissolved in H_2O (6 ml) was then added and the mixture was stirred for 3 hr at rt under N_2. To workup, the mixture was acidified to pH~3 with 1M HCl solution, which allowed the product to be extracted with EtOAc. The organic layer was washed with H_2O, brine, dried over sodium sulfate, and evaporated under vacuum. Flash column chromatography using a gradient of 0-20% EtOAc in Hex was used to purify the desired compound. Yield: 292 mg, 84%. R_f =0.21 (EtOAc/Hex, 1:4). **^{1}H NMR** ($CDCl_3$, 300 MHz) δ 8.09 (dd, *J* = 5.8, 8.8 Hz, 1H), 7.33-7.29 (m, 2H), 7.10 (td, *J* = 2.6, 8.7 Hz, 1H), 1.15 (s, 21H); **^{13}C NMR** ($CDCl_3$, 75 MHz): δ 170.0, 166.7, 163.3, 134.0, 133.9, 122.1, 121.8, 116.0, 115.7, 103.6, 100.3, 18.6, 11.3. **HRMS** for $C_{18}H_{25}FO_2Si$ $[M+Na]^+$ calcd. 343.1500, found 343.1508.

Preparation of **16j** :

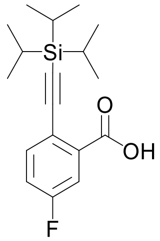

Compound **15j** (361 mg, 1.08 mmol) was dissolved in MeOH (6 ml) and THF (6 ml) at 0°C. KOH (607 mg, 10.83 mmol) dissolved in H_2O (6 ml) was then added and the mixture was stirred for 2.5 hr at rt under N_2. To workup, the mixture was acidified to pH~3 with 1M HCl solution, which allowed the product to be extracted with EtOAc. The organic layer was washed with H_2O, brine, dried over sodium sulfate, and evaporated under vacuum. Flash column chromatography using a gradient of 10-25% EtOAc in Hex was used to purify the desired compound. Yield: 300 mg, 87%. R_f =0.25 (EtOAc/Hex, 1:4). **^{1}H NMR** ($CDCl_3$, 300 MHz) δ 7.78 (dd, *J* = 2.7, 9.1 Hz, 1H), 7.63 (dd, *J* = 5.4, 8.6 Hz, 1H), 7.25-7.20 (m, 1H), 1.15 (s, 21H); **^{13}C NMR** ($CDCl_3$, 75 MHz): δ 165.9, 160.4, 137.1, 137.0, 120.3, 120.0, 118.6, 118.3, 102.8, 99.3, 18.6, 11.3. **HRMS** for $C_{18}H_{25}FO_2Si$ $[M+Na]^+$ calcd. 343.1500, found 343.1501.

Preparation of **16k** :

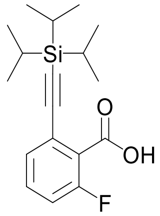

Compound **15k** (361 mg, 1.08 mmol) was dissolved in MeOH (6 ml) and THF (6 ml) at 0°C. KOH (607 mg, 10.83 mmol) dissolved in H_2O (6 ml) was then added and the mixture was stirred for 3 hr at 50°C under N_2. To workup, the mixture was acidified to pH~3 with 1M HCl solution, which allowed the product to be extracted with EtOAc. The organic layer was washed with H_2O, brine, dried over sodium sulfate, and evaporated under vacuum. Flash column chromatography using a gradient of 10-25% EtOAc in Hex was used to purify the desired compound. Yield: 251 mg, 72%. R_f =0.11 (EtOAc/Hex, 1:4). **^{1}H NMR** ($CDCl_3$, 300 MHz) δ 7.42-7.35 (m, 2H), 7.14-7.08 (m, 1H), 1.12 (s, 1H); **^{13}C NMR** ($CDCl_3$, 75 MHz): δ 161.7, 158.4, 132.1, 132.0, 129.5(2), 116.6, 116.3, 102.6, 98.0, 18.6, 11.3. **HRMS** for $C_{18}H_{25}FO_2Si$ $[M+Na]^+$ calcd. 343.1500, found 343.1502.

Preparation of **16l** :

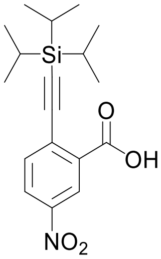

Compound **15l** (337 mg, 0.878 mmol) was dissolved in MeOH (6 ml) and THF (6 ml) at 0°C. KOH (492 mg, 8.78 mmol) dissolved in H_2O (6 ml) was then added and the mixture was stirred for 1 hr at rt under N_2. To workup, the mixture was acidified to pH~3 with 1M HCl solution, which allowed the product to be extracted with EtOAc. The organic layer was washed with H_2O, brine, dried over sodium sulfate, and evaporated under vacuum. Flash column chromatography using a gradient of 25-50% EtOAc in Hex was used to purify the desired compound. Yield: 296 mg, 97%. R_f =0.33 (EtOAc/Hex, 1:1). **^{1}H NMR** ($CDCl_3$, 300 MHz) δ 8.75 (s, 1H), 8.16 (d, *J* = 8.0 Hz, 1H), 7.65 (d, *J* = 8.5 Hz, 1H), 1.04 (s, 21H). **^{13}C NMR** ($CDCl_3$, 75 MHz): δ 146.6, 136.4, 130.2, 126.3, 126.2, 125.5(2), 104.6, 104.3, 58.8, 18.6, 11.3. **HRMS** for $C_{18}H_{25}NO_4Si$ $[M+Na]^+$ calcd. 370.1445, found 370.1449.

Preparation of **16m** :

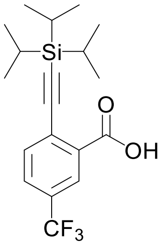

Compound **15m** (148 mg, 0.385 mmol) was dissolved in MeOH (6 ml) and THF (6 ml) at 0°C. KOH (216 mg, 3.85 mmol) dissolved in H_2O (6 ml) was then added and the mixture was stirred for 2.5 hr at rt under N_2. To workup, the mixture was acidified to pH~3 with 1M HCl solution, which allowed the product to be extracted with EtOAc. The organic layer was washed with H_2O, brine, dried over sodium sulfate, and evaporated under vacuum. Flash column chromatography using a gradient of 25-50% EtOAc in Hex was used to purify the desired compound. Yield: 85 mg, 59%. R_f=0.59 (EtOAc/Hex, 1:1). **^{1}H NMR** ($CDCl_3$, 300 MHz) δ 8.30 (s, 1H), 7.75 (s, 2H), 1.15 (s, 21H); **^{13}C NMR** ($CDCl_3$, 75 MHz): δ 169.6, 135.8, 131.9, 130.5, 130.1, 129.0, 128.2. 128.0, 103.5, 102.5, 18.6, 11.3. **HRMS** for $C_{19}H_{25}F_3O_2Si$ $[M+Na]^+$ calcd. 393.1468, found 393.1474.

Preparation of **16n** :

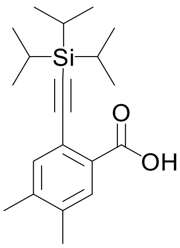

Compound **15n** (338 mg, 0.982 mmol) was dissolved in MeOH (6 ml) and THF (6 ml) at 0°C. KOH (551 mg, 9.82 mmol) dissolved in H_2O (6 ml) was then added and the mixture was stirred for 2 hr at 50°C under N_2. To workup, the mixture was acidified to pH~3 with 1M HCl solution, which allowed the product to be extracted with EtOAc. The organic layer was washed with H_2O, brine, dried over sodium sulfate, and evaporated under vacuum. Flash column chromatography using a gradient of 10-20% EtOAc in Hex was used to purify the desired compound. Yield: 320 mg, quan. R_f=0.33 (EtOAc/Hex, 1:5). **^{1}H NMR** ($CDCl_3$, 300 MHz) δ 7.87 (s, 1H), 7.38 (s, 1H), 2.31 (s, 3H), 2.29 (s, 3H), 1.15 (s, 21H); **^{13}C NMR** ($CDCl_3$, 75 MHz): δ 169.6, 142.2, 137.9, 136.0, 132.6, 128.8, 120.9, 105.2, 98.0, 19.7, 19.6, 18.7, 11.4. **HRMS** for $C_{20}H_{30}O_2Si$ $[M+H]^+$ calcd. 331.2088, found 331.2080.

Preparation of **16o** :

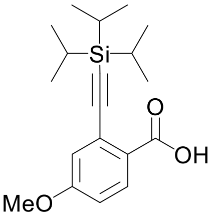

Compound **15o** (360 mg, 1.04 mmol) was dissolved in MeOH (6 ml) and THF (6 ml) at 0°C. KOH (584 mg, 10.41 mmol) dissolved in H_2O (6 ml) was then added and the mixture was stirred for 5 hr at rt under N_2. To workup, the mixture was acidified to pH~3 with 1M HCl solution, which allowed the product to be extracted with EtOAc. The organic layer was washed with H_2O, brine, dried over sodium sulfate, and evaporated under vacuum. Flash column chromatography using a gradient of 0-50% EtOAc in Hex was used to purify the desired compound. Yield: 276 mg, 80%. R_f=0.26 (EtOAc/Hex, 1:4). **^{1}H NMR** ($CDCl_3$, 300 MHz) δ 8.05 (d, J = 8.8 Hz, 1H), 7.08 (d, J = 2.3 Hz, 1H), 6.89 (dd, J = 2.4, 8.8 Hz, 1H), 3.86 (s, 3H), 1.16 (s, 21H); **^{13}C NMR** ($CDCl_3$, 75 MHz): δ 162.6, 133.7, 128.8, 128.7, 126.0, 119.9, 114.4, 105.0, 98.5, 55.7, 18.7, 11.4. **HRMS** for $C_{19}H_{28}O_3Si$ $[M+H]^+$ calcd. 333.1880, found 333.1872.

Preparation of **16p** :

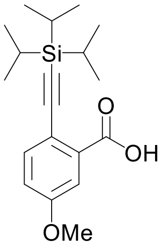

Compound **15p** (379 mg, 1.10 mmol) was dissolved in MeOH (6 ml) and THF (6 ml) at 0°C. KOH (614 mg, 10.95 mmol) dissolved in H_2O (6 ml) was then added and the mixture was stirred for 1 hr at rt under N_2. To workup, the mixture was acidified to pH~3 with 1M HCl solution, which allowed the product to be extracted with EtOAc. The organic layer was washed with H_2O, brine, dried over sodium sulfate, and evaporated under vacuum. Flash column chromatography using a gradient of 0-50% EtOAc in Hex was used to purify the desired compound. Yield: 361 mg, quan. R_f =0.09 (EtOAc/Hex, 1:4). **^{1}H NMR** ($CDCl_3$, 300 MHz) δ 7.58-7.54 (m, 2H), 7.04 (dd, *J* = 2.8, 8.6 Hz, 1H), 3.87 (s, 3H), 1.15 (s, 21H); **^{13}C NMR** ($CDCl_3$, 75 MHz): δ 170.4, 159.5, 136.5, 132.9, 119.1, 116.2, 115.6, 104.8, 96.8, 55.7, 18.7, 11.4. **HRMS** for $C_{19}H_{28}O_3Si$ $[M+Na]^+$ calcd. 355.1700, found 355.1706.

Preparation of **17a** :

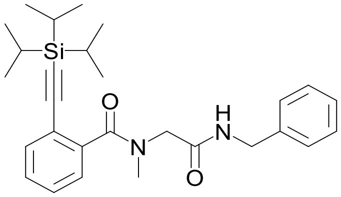

Compound **16a** (199 mg, 0.660 mmol), **2a** (118 mg, 0.660 mmol), EDC (204 mg, 1.32 mmol), HOBt (178 mg, 1.32 mmol) were dissolved in DMF (10 ml). NEt_3 (185 μl, 1.32 mmol) was then added and the mixture was stirred for 16 hr at room temperature under N_2. To workup, the reaction was diluted with EtOAc and then the organic layer was washed with H_2O, brine, dried over sodium sulfate, and evaporated under vacuum. Flash column chromatography using a gradient of 10-20% EtOAc in Hex was used to purify the desired compound. Yield: 236 mg, 77%. R_f =0.46 (EtOAc/Hex, 1:1). **^{1}H NMR** ($CDCl_3$, 300 MHz, mixture of rotamers) δ 7.55-7.48 (m, 1H), 7.38-7.28 (m, 6H), 7.23-7.14 (m, 2H), 6.98 (br s, 1H), 4.48 (d, *J*= 5.9, 2H), 3.98 (s, 2H), 3.02 (s, 3H), 1.10 (s, 21H); **^{13}C NMR** ($CDCl_3$, 75 MHz): δ 171.0, 168.5, 138.0, 132.8, 129.0, 128.7, 128.5, 127.5, 127.3, 127.2, 126.6, 120.0, 103.3, 94.7, 54.3, 52.0, 43.0, 37.5, 34.0, 18.2, 10.8. **HRMS** for $C_{28}H_{38}N_2O_2Si$ $[M+H]^+$ calcd. 463.2775, found 463.2770.

Preparation of **17b** :

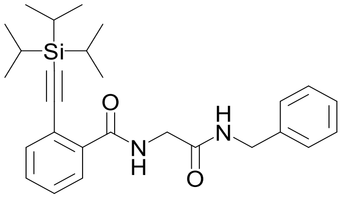

Compound **16a** (100 mg, 0.330 mmol), **2b** (54 mg, 0.330 mmol), EDC (102 mg, 0.660 mmol), HOBt (89 mg, 0.660 mmol) were dissolved in DMF (10 ml). NEt_3 (93 μl, 0.660 mmol) was then added and the mixture was stirred for 16 hr at room temperature under N_2. To workup, the reaction was diluted with EtOAc and then the organic layer was washed with H_2O, brine, dried over sodium sulfate, and evaporated under vacuum. Flash column chromatography using a gradient of 10-25% EtOAc in Hex was used to purify the desired compound. Yield: 134 mg, 91%. R_f =0.37 (EtOAc/Hex, 1:1). **^{1}H NMR** ($CDCl_3$, 300 MHz) δ 8.36 (br s, 1H), 8.07-8.04 (m, 1H), 7.59-7.56 (m, 1H), 7.45-7.42 (m, 2H), 7.33-7.23 (m, 5H), 6.71 (br s, 1H), 4.46 (d, *J*= 5.9, 2H), 4.16 (d, *J*= 5.8, 2H), 1.13 (s, 21H); **^{13}C NMR** ($CDCl_3$, 75 MHz): δ 169.0, 167.2, 138.2, 134.8, 134.3, 131.3, 130.5, 129.3, 128.9, 127.8, 127.7, 120.2, 105.5, 99.8, 44.5, 43.5, 18.7, 11.2. **HRMS** for $C_{27}H_{36}N_2O_2Si$ $[M+H]^+$ calcd. 449.2619, found 449.2627.

Preparation of **17c** :

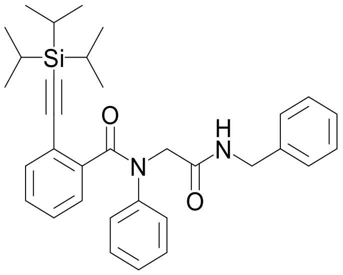

Compound **16a** (222 mg, 0.734 mmol), **2c** (177 mg, 0.735 mmol), EDC (228 mg, 1.47 mmol), HOBt (199 mg, 1.47 mmol) were dissolved in $CHCl_3$ (10 ml). NEt_3 (206 μl, 1.47 mmol) was then added and the mixture was stirred for 16 hr at room temperature under N_2. To workup, the reaction was diluted with EtOAc and then the organic layer was washed with H_2O, brine, dried over sodium sulfate, and evaporated under vacuum. Flash column chromatography using a gradient of 10-20% EtOAc in Hex was used to purify the desired compound. Yield: 93 mg, 24%. R_f =0.63 (EtOAc/Hex, 1:1). **^{1}H NMR** ($CDCl_3$, 300 MHz) δ 7.43-7.23 (m, 8H), 7.15-7.05 (m, 6H), 4.53-4.47 (m, 4H), 1.15 (s, 21H); **^{13}C NMR** ($CDCl_3$, 75 MHz): δ 170.9, 168.9, 142.6, 138.4, 133.1, 129.8, 129.2, 129.0(2), 128.8, 128.1, 128.0, 127.9, 127.7, 127.4, 127.3, 80.4, 80.1, 54.3, 43.7, 18.7, 11.4. **HRMS** for $C_{33}H_{40}N_2O_2Si$ $[M+H]^+$ calcd. 525.2932, found 525.2928.

Preparation of **17d** :

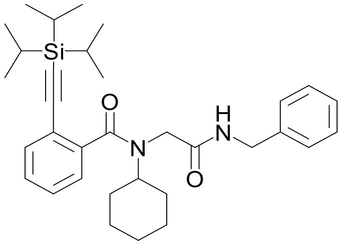

Compound **16a** (110 mg, 0.364 mmol), **2d** (90 mg, 0.364 mmol), HCTU (300 mg, 0.728 mmol), HOBt(6-Cl) (123 mg, 0.728 mmol) were dissolved in DMF (5 ml). DIPEA (247 μl, 1.46 mmol) was then added and the mixture was stirred for 16 hr at room temperature under N_2. To workup, the reaction was diluted with EtOAc and then the organic layer was washed with H_2O, brine, dried over sodium sulfate, and evaporated under vacuum. Flash column chromatography using a gradient of 10-30% EtOAc in Hex was used to purify the desired compound. Yield: 181 mg, 93%. R_f =0.59 (EtOAc/Hex, 1:1). **^{1}H NMR** ($CDCl_3$, 300 MHz) δ 7.54-7.50 (m, 1H), 7.43-7.27 (m, 8H), 7.02-6.96 (m, 1H), 4.67-4.49 (m, 2H), 4.39-4.24 (m, 1H), 3.74-3.61 (m, 1H), 3.29-3.19 (m, 1H), 2.00-1.85 (m, 1H), 1.73-1.65(m, 2H), 1.58-1.43 (m, 4H), 1.06 (s, 21H), 1.02-0.85 (m, 3H). **^{13}C NMR** ($CDCl_3$, 75 MHz): δ 171.7, 170.7, 139.2, 138.6, 133.7, 128.9 (2), 128.8, 127.7, 127.5, 125.7, 120.2, 104.0, 95.5, 59.9, 46.5, 43.5, 31.7, 31.0, 30.4, 25.6, 24.9, 18.6, 11.3. **HRMS** for $C_{33}H_{46}N_2O_2Si$ $[M+H]^+$ calcd. 531.3401, found 531.3410.

Preparation of **17e** :

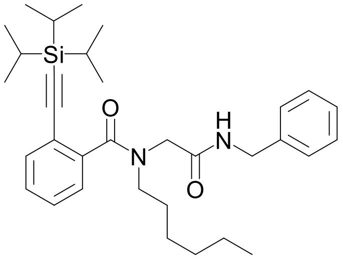

Compound **16a** (121 mg, 0.400 mmol), **2e** (99 mg, 0.400 mmol), HCTU (331 mg, 0.801 mmol), HOBt(6-Cl) (136 mg, 0.801 mmol) were dissolved in DMF (5 ml). DIPEA (272 μl, 1.60 mmol) was then added and the mixture was stirred for 16 hr at room temperature under N_2. To workup, the reaction was diluted with EtOAc and then the organic layer was washed with H_2O, brine, dried over sodium sulfate, and evaporated under vacuum. Flash column chromatography using a gradient of 15-30% EtOAc in Hex was used to purify the desired compound. Yield: 210 mg, quan. R_f =0.64 (EtOAc/Hex, 1:1). **^{1}H NMR** ($CDCl_3$, 300 MHz) δ 7.12-7.05 (m, 1H), 6.98-6.87 (m,

7H), 6.76-6.66 (m, 2H), 4.12-3.88 (m, 2H), 2.79 (s, 2H), 1.13-1.00 (m, 1H), 0.92-0.85 (m, 1H), 0.74-0.54 (m, 8H), 0.67 (s, 21H), 0.35 (t, *J* = 6.9, 3H). ^{13}**C NMR** ($CDCl_3$, 75 MHz): δ 171.6, 170.0, 168.4, 138.7, 138.5, 133.3, 129.1, 128.8(2), 127.9, 127.8, 127.6, 126.7, 120.5, 103.8, 95.4, 51.9, 51.8, 43.4, 31.0, 28.4, 26.1, 22.4, 18.7, 13.9, 11.3. **HRMS** for $C_{33}H_{48}N_2O_2Si$ $[M+Na]^+$ calcd. 555.3377, found 555.3387.

Preparation of **17f** :

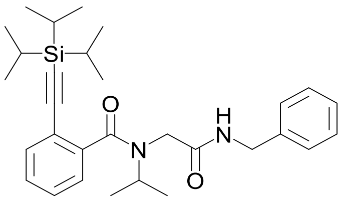

Compound **16a** (130 mg, 0.430 mmol), **2f** (89 mg, 0.430 mmol), EDC (133 mg, 0.860 mmol), HOBt (116 mg, 0.860 mmol) were dissolved in DMF (5 ml). NEt_3 (240 µl, 1.72 mmol) was then added and the mixture was stirred for 16 hr at room temperature under N_2. To workup, the reaction was diluted with EtOAc and then the organic layer was washed with H_2O, brine, dried over sodium sulfate, and evaporated under vacuum. Flash column chromatography using a gradient of 15-30% EtOAc in Hex was used to purify the desired compound. Yield: 210 mg, quan. R_f =0.54 (EtOAc/Hex, 1:1). ^{1}**H NMR** ($CDCl_3$, 300 MHz) δ 7.53-7.56 (m, 1H), 7.28-7.45 (m, 7H), 7.01-7.04 (m, 1H), 4.72-4.52 (m, 2H), 4.41-4.24 (m, 2H), 3.76 (quin, *J* = 6.7 Hz, 1H), 1.23 (m, 6H), 1.09 (m, 21H); ^{13}**C NMR** ($CDCl_3$, 75 MHz): δ 171.5, 170.6, 139.2, 138.7, 133.8, 128.9(2), 128.8, 127.7, 127.5, 125.6, 120.3, 103.9, 95.6, 51.4, 45.5, 43.5, 18.7(2), 11.3. **HRMS** for $C_{30}H_{42}N_2O_2Si$ $[M+H]^+$ calcd. 491.3088, found 491.3092.

Preparation of **17g** :

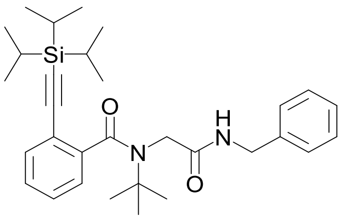

Compound **16a** (140 mg, 0.463 mmol), **2g** (102 mg, 0.463 mmol), HCTU (383 mg, 0.926 mmol), HOBt(6-Cl) (157 mg, 0.926 mmol) were dissolved in DMF (5 ml). DIPEA (260 µl, 1.85 mmol) was then added and the mixture was stirred for 16 hr at room temperature under N_2. To workup, the reaction was diluted with EtOAc and then the organic layer was washed with H_2O, brine, dried over sodium sulfate, and evaporated under vacuum. Flash column chromatography using a gradient of 10-25% EtOAc in Hex was used to purify the desired compound. Yield: 26 mg, 11%. R_f =0.14 (EtOAc/Hex, 1:4). ^{1}**H NMR** ($CDCl_3$, 300 MHz) δ 7.46 (d, *J*= 8.7, 1H), 7.36-7.16 (m, 7H), 7.10 (d, *J*= 7.1, 1H), 4.40 (d, *J*= 5.9, 2H), 4.00 (s, 2H), 1.54 (s, 9H), 1.10 (s, 21H); ^{13}**C NMR** ($CDCl_3$, 75 MHz): δ 164.8, 162.6, 137.9, 134.0, 131.8, 129.0, 128.9, 128.6, 128.0, 127.9, 127.5, 126.2, 95.6, 92.5, 58.5, 50.9, 43.6, 28.7, 18.7, 11.3. **HRMS** for $C_{31}H_{44}N_2O_2Si$ $[M+H]^+$ calcd. 505.3245, found 505.3247.

Preparation of **17h** :

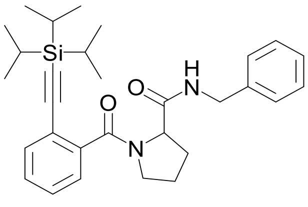

Compound **16a** (132 mg, 0.437 mmol), **2h** (89 mg, 0.437 mmol), HCTU (361 mg, 0.873 mmol), HOBt(6-Cl) (148 mg, 0.873 mmol) were dissolved in DMF (5 ml). DIPEA (296 µl, 1.75 mmol) was then added and the mixture was stirred for 16 hr at room temperature under N_2. To workup, the reaction was diluted with EtOAc and then the organic layer was washed with H_2O, brine, dried over sodium sulfate, and evaporated under vacuum. Flash column chromatography using a gradient of 25-50% EtOAc in Hex was used to purify the desired compound. Yield: 213 mg, quan. R_f =0.42 (EtOAc/Hex,1:1). ^{1}**H NMR** ($CDCl_3$, 300 MHz) δ 7.92-7.82 (m, 2H), 7.71 (d, *J*=

1.7, 1H), 7.51-7.02 (m, 6H), 4.63-4.38 (m, 1H), 4.26-4.03 (m, 1H), 3.84-3.63 (m, 1H), 3.49-3.25 (m, 2H), 2.32-2.17 (m, 1H), 2.10-1.82 (m, 3H), 1.10 (s, 21H); **^{13}C NMR** ($CDCl_3$, 75 MHz): δ 174.3, 171.4, 134.9, 134.0, 130.5, 130.4, 130.1, 129.6, 128.9, 128.4, 128.2, 127.5, 121.2, 110.5, 63.0, 61.5, 44.0, 33.2, 31.3, 25.7, 23.8, 19.1, 12.4. **HRMS** for $C_{30}H_{40}N_2O_2Si$ $[M+H]^+$ calcd. 489.2932, found 489.2942.

Preparation of **17i** :

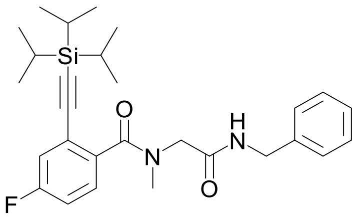

Compound **16i** (132 mg, 0.413 mmol), **2a** (88 mg, 0.495 mmol), EDC (128 mg, 0.825 mmol), HOBt (111 mg, 0.825 mmol) were dissolved in DMF (8 ml). NEt_3 (230 μl, 1.65 mmol) was then added and the mixture was stirred for 16 hr at room temperature under N_2. To workup, the reaction was diluted with EtOAc and then the organic layer was washed with H_2O, brine, dried over sodium sulfate, and evaporated under vacuum. Flash column chromatography using a gradient of 30-50% EtOAc in Hex was used to purify the desired compound. Yield: 167 mg, 84%. R_f =0.43 (EtOAc/Hex, 1:1). **^{1}H NMR** ($CDCl_3$, 300 MHz, mixture of rotamers) δ 7.37-7.28 (m, 4H), 7.22-7.16 (m, 3H), 7.10-7.03 (m, 1H), 4.48 (d, *J*= 5.8, 2H), 4.07 (s, 2H), 3.02 (s, 3H), 1.10 (s, 21H); **^{13}C NMR** ($CDCl_3$, 75 MHz): δ 170.7, 168.8, 163.5, 129.3, 129.0(2), 128.0, 127.8, 120.0, 116.8, 116.5, 115.8, 102.6, 94.9, 55.8, 52.5, 43.5, 38.0, 25.8, 18.7, 11.2. **HRMS** for $C_{28}H_{37}FN_2O_2Si$ $[M+H]^+$ calcd. 481.2681, found 481.2685.

Preparation of **17j** :

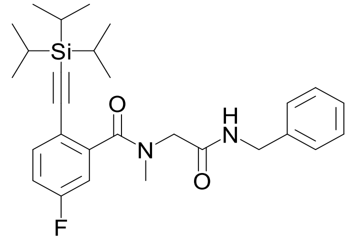

Compound **16j** (132 mg, 0.413 mmol), **2a** (88 mg, 0.495 mmol), EDC (128 mg, 0.825 mmol), HOBt (111 mg, 0.825 mmol) were dissolved in DMF (8 ml). NEt_3 (230 μl, 1.65 mmol) was then added and the mixture was stirred for 16 hr at room temperature under N_2. To workup, the reaction was diluted with EtOAc and then the organic layer was washed with H_2O, brine, dried over sodium sulfate, and evaporated under vacuum. Flash column chromatography using a gradient of 15-30% EtOAc in Hex was used to purify the desired compound. Yield: 198 mg, quan. R_f =0.59 (EtOAc/Hex, 1:1). **^{1}H NMR** ($CDCl_3$, 300 MHz, mixture of rotamers) δ 7.52-7.45 (m, 1H), 7.38-7.28 (m, 3H), 7.15-6.88 (m, 4H), 4.46 (d, *J*= 5.9, 2H), 3.97 (s, 2H), 3.02 (s, 3H), 1.09 (s, 21H); **^{13}C NMR** ($CDCl_3$, 75 MHz): δ 169.9, 168.5, 167.7, 140.5, 138.3, 135.3, 129.0, 127.7, 116.9, 116.6, 114.6, 114.3, 102.6, 94.9, 54.5, 52.2, 43.5, 37.8, 34.4, 18.6, 11.2. **HRMS** for $C_{28}H_{37}FN_2O_2Si$ $[M+H]^+$ calcd. 481.2681, found 481.2691.

Preparation of **17k** :

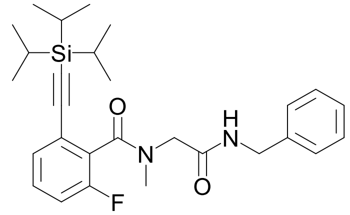

Compound **16k** (143 mg, 0.447 mmol), **2a** (95 mg, 0.536 mmol), HCTU (369 mg, 0.894 mmol), HOBt(6-Cl) (152 mg, 0.894 mmol) were dissolved in DMF (10 ml). DIPEA (303 μl, 1.79 mmol) was then added and the mixture was stirred for 16 hr at room temperature under N_2. To workup, the reaction was diluted with EtOAc and then the organic layer was washed with H_2O, brine, dried over sodium sulfate, and evaporated under vacuum. Flash column chromatography using a gradient of 15-30% EtOAc in Hex was used to purify the desired compound.

Yield: 181 mg, 84%. R_f =0.50 (EtOAc/Hex, 1:1). **^{1}H NMR** ($CDCl_3$, 300 MHz, mixture of rotamers) δ 7.36-7.28 (m, 8H), 7.12-6.97 (m, 1H), 6.77 (br s, 1H), 4.65-4.37 (m, 3H), 3.47-3.37 (m, 1H), 2.99 (s, 3H), 1.10 (s, 21H); **^{13}C NMR** ($CDCl_3$, 75 MHz): δ 168.4, 167.8, 166.4, 138.0, 137.6, 130.8, 130.7, 129.0, 128.9, 127.9, 127.7, 122.6, 116.5, 116.2, 102.2, 96.7, 54.8, 52.1, 43.6, 37.3, 34.1, 18.6, 11.2. **HRMS** for $C_{28}H_{37}FN_2O_2Si$ $[M+H]^+$ calcd. 481.2681, found 481.2685.

Preparation of **17l** :

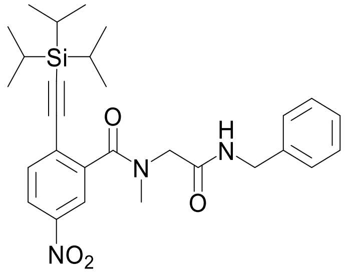

Compound **16l** (207 mg, 0.561 mmol), **2a** (120 mg, 0.674 mmol), EDC (174 mg, 1.12 mmol), HOBt (152 mg, 1.12 mmol) were dissolved in DMF (5 ml). NEt_3 (314 µl, 2.25 mmol) was then added and the mixture was stirred for 16 hr at room temperature under N_2. To workup, the reaction was diluted with EtOAc and then the organic layer was washed with H_2O, brine, dried over sodium sulfate, and evaporated under vacuum. Flash column chromatography using a gradient of 15-30% EtOAc in Hex was used to purify the desired compound. Yield: 207 mg, 73%. R_f =0.61 (EtOAc/Hex, 1:1). **^{1}H NMR** ($CDCl_3$, 300 MHz, mixture of rotamers) δ 8.26-8.13 (m, 2H), 7.71-7.61 (m, 1H), 7.43-7.29 (m, 5H), 6.87 (br s, 1H), 4.48 (d, *J*= 5.9, 2H), 3.97 (s, 2H), 3.03 (s, 3H), 1.11 (s, 21H); **^{13}C NMR** ($CDCl_3$, 75 MHz): δ 168.9, 168.1, 167.2, 147.3, 139.8, 138.2, 134.2, 129.0, 127.8, 127.7, 126.7, 124.1, 122.6, 101.8, 54.4, 52.1, 43.5, 37.9, 34.5, 18.6, 11.1. **HRMS** for $C_{28}H_{37}N_3O_4Si$ $[M+H]^+$ calcd. 508.2626, found 508.2622.

Preparation of **17m** :

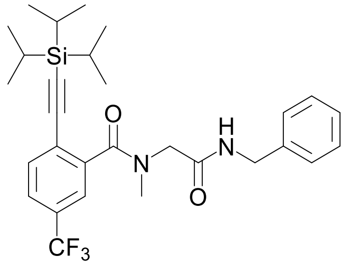

Compound **16m** (85 mg, 0.229 mmol), **2a** (41 mg, 0.229 mmol), EDC (72 mg, 0.459 mmol), HOBt (62 mg, 0.459 mmol) were dissolved in DMF (8 ml). NEt_3 (128 µl, 0.918 mmol) was then added and the mixture was stirred for 16 hr at room temperature under N_2. To workup, the reaction was diluted with EtOAc and then the organic layer was washed with H_2O, brine, dried over sodium sulfate, and evaporated under vacuum. Flash column chromatography using a gradient of 25-50% EtOAc in Hex was used to purify the desired compound. Yield: 120 mg, quan. R_f =0.16 (EtOAc/Hex, 1:4). **^{1}H NMR** ($CDCl_3$, 300 MHz, mixture of rotamers) δ 7.66-7.45 (m, 4H), 7.37-7.25 (m, 4H), 6.94 (br s, 1H), 4.48 (d, *J*= 5.8, 2H), 3.99 (s, 2H), 3.02 (s, 3H), 1.11 (s, 21H); **^{13}C NMR** ($CDCl_3$, 75 MHz): δ 169.9, 168.4, 167.5, 139.1, 138.3, 133.6, 128.9, 127.8, 127.7, 126.1, 124.0, 121.7, 102.3, 98.6, 54.5, 52.2, 43.5, 37.9, 34.4, 18.6, 11.2. **HRMS** for $C_{29}H_{37}F_3N_2O_2Si$ $[M+H]^+$ calcd. 531.2649, found 531.2653.

Preparation of **17n** :

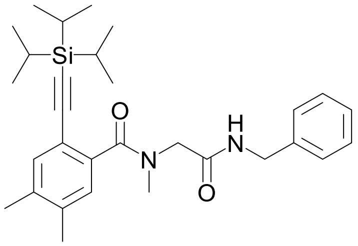

Compound **16n** (168 mg, 0.509 mmol), **2a** (109 mg, 0.610 mmol), EDC (158 mg, 1.02 mmol), HOBt (137 mg, 1.02 mmol) were dissolved in DMF (10 ml). NEt_3 (345 µl, 2.03 mmol) was then added and the mixture was stirred for 16 hr at room temperature under N_2. To workup, the reaction was diluted with EtOAc and then the organic layer was washed with H_2O, brine, dried over sodium sulfate, and evaporated under vacuum. Flash column chromatography using a gradient of 25-50% EtOAc in Hex was used to purify the desired compound. Yield: 243 mg, 97%. R_f =0.53 (EtOAc/Hex, 1:1). **^{1}H NMR** ($CDCl_3$, 300 MHz, mixture of rotamers) δ 7.37-7.27 (m, 6H), 7.07-6.99 (m, 1H), 6.95 (br s, 1H), 4.48 (d, *J*= 5.8, 2H), 4.01 (s, 2H), 3.02 (s, 3H), 2.24 (s, 6H), 1.09 (s, 21H); **^{13}C NMR** ($CDCl_3$, 75 MHz): δ 171.8, 169.2, 168.2, 138.5, 138.3, 135.9, 134.0, 128.9, 128.3, 127.7, 127.6, 117.7, 104.2, 93.7, 54.9, 52.7, 43.4, 37.9, 34.3, 19.7, 19.4, 18.7, 11.3. **HRMS** for $C_{30}H_{42}N_2O_2Si$ $[M+H]^+$ calcd. 491.3088, found 491.3097.

Preparation of **17o** :

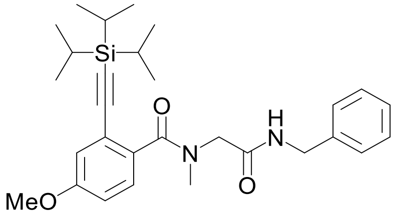

Compound **16o** (126 mg, 0.379 mmol), **2a** (88 mg, 0.493 mmol), EDC (117 mg, 0.758 mmol), HOBt (102 mg, 0.758 mmol) were dissolved in DMF (10 ml). NEt_3 (212 µl, 1.52 mmol) was then added and the mixture was stirred for 16 hr at room temperature under N_2. To workup, the reaction was diluted with EtOAc and then the organic layer was washed with H_2O, brine, dried over sodium sulfate, and evaporated under vacuum. Flash column chromatography using a gradient of 0-50% EtOAc in Hex was used to purify the desired compound. Yield: 186 mg, quan. R_f =0.36 (EtOAc/Hex, 1:1). **^{1}H NMR** ($CDCl_3$, 300 MHz, mixture of rotamers) δ 7.36-7.28 (m, 4H), 7.17-7.08 (m, 3H), 6.99-6.87 (m, 2H), 4.47 (d, *J*= 5.9, 2H), 4.13 (s, 2H), 3.82 (s, 3H), 3.03 (s, 3H), 1.10 (s, 21H); **^{13}C NMR** ($CDCl_3$, 75 MHz): δ 171.5, 169.1, 160.2, 138.5, 130.9, 128.9, 127.9, 127.7, 127.6, 121.8, 117.8, 115.5, 103.8, 95.0, 55.6, 54.9, 52.6, 43.4, 38.0, 34.5, 18.7, 11.3. **HRMS** for $C_{29}H_{40}N_2O_3Si$ $[M+H]^+$ calcd. 493.2881, found 493.2887.

Preparation of **17p** :

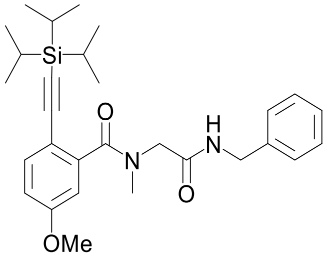

Compound **16p** (176 mg, 0.530 mmol), **2a** (142 mg, 0.795 mmol), EDC (164 mg, 1.06 mmol), HOBt (143 mg, 1.06 mmol) were dissolved in DMF (10 ml). NEt_3 (289 µl, 2.12 mmol) was then added and the mixture was stirred for 16 hr at room temperature under N_2. To workup, the reaction was diluted with EtOAc and then the organic layer was washed with H_2O, brine, dried over sodium sulfate, and evaporated under vacuum. Flash column chromatography using a gradient of 20-50% EtOAc in Hex was used to purify the desired compound. Yield: 178 mg, 68%. R_f =0.36 (EtOAc/Hex, 1:1). **^{1}H NMR** ($CDCl_3$, 300 MHz, mixture of rotamers) δ 7.45-7.28 (m, 5H), 6.94-6.70 (m, 4H), 4.48 (d, *J*= 5.9, 2H), 4.13 (s, 2H), 3.78 (s, 3H), 3.04 (s, 3H), 1.09 (s, 21H); **^{13}C NMR**

($CDCl_3$, 75 MHz): δ 171.2, 168.9, 160.2, 140.0, 138.4, 134.8, 128.9, 127.7, 115.9, 112.6, 111.9, 111.7, 103.8, 93.1, 55.6, 55.5, 52.5, 43.4, 37.9, 34.3, 18.7, 11.3. **HRMS** for $C_{29}H_{40}N_2O_3Si$ $[M+H]^+$ calcd. 493.2881, found 493.2890.

Preparation of **1a** :

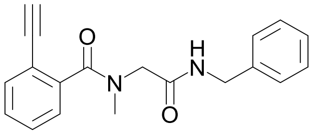

Compound **17a** (236 mg, 0.510 mmol) was dissolved in DCM (2 ml). TBAF (764 μl of 1.0M solution, 0.764 mmol) was then added and the mixture was stirred for 1 hr at room temperature under N_2. To workup, the reaction was diluted with EtOAc and then the organic layer was washed with H_2O, brine, dried over sodium sulfate, and evaporated under vacuum. Flash column chromatography using a gradient of 50-100% EtOAc in Hex was used to purify the desired compound. Yield: 155 mg, quan. R_f =0.13 (EtOAc/Hex, 1:1). **^{1}H NMR** ($CDCl_3$, 300 MHz, mixture of rotamers) δ 7.51-7.29 (m, 9H), 6.88 (br s, 1H), 4.49 (d, *J*= 5.6, 2H), 4.30 (s, 2H), 2.96 (s, 3H), 2.40 (s, 1H); **^{13}C NMR** ($CDCl_3$, 75 MHz): δ 170.9, 168.4, 138.7, 138.1, 133.2, 129.7, 129.5, 129.0, 128.3, 127.9, 126.6, 118.9, 81.2, 81.0, 55.0, 52.0, 43.7, 37.9, 34.0. **HRMS** for $C_{19}H_{18}N_2O_2$ $[M+Na]^+$ calcd. 329.1260, found 329.1270.

Preparation of **1b** :

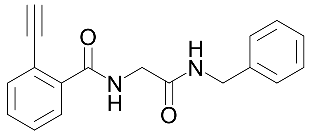

Compound **17b** (134 mg, 0.299 mmol) was dissolved in DCM (2 ml). TBAF (450 μl of 1.0M solution, 0.448 mmol) was then added and the mixture was stirred for 1 hr at room temperature under N_2. To workup, the reaction was diluted with EtOAc and then the organic layer was washed with H_2O, brine, dried over sodium sulfate, and evaporated under vacuum to give a white precipitate, which was then washed with hexanes and filtered to give the desired compound. Yield: 42 mg, 48%. R_f =0.29 (EtOAc/Hex, 1:1). **^{1}H NMR** ($CDCl_3$, 300 MHz) δ 8.13-8.05 (m, 1H), 7.76-7.69 (m, 1H), 7.56-7.49 (m, 1H), 7.42-7.19 (m, 6H), 4.44 (d, *J*= 5.7, 2H), 4.24 (d, *J*= 5.0, 2H), 3.36 (s, 1H); **^{13}C NMR** ($CDCl_3$, 75 MHz): δ 168.8, 166.8, 138.1, 135.8, 134.3, 130.9, 129.7, 129.4, 128.8, 128.0, 127.7, 119.1, 84.3, 81.9, 44.2, 43.6. **HRMS** for $C_{18}H_{16}N_2O_2$ $[M+Na]^+$ calcd. 315.1104, found 315.1110.

Preparation of **1c** :

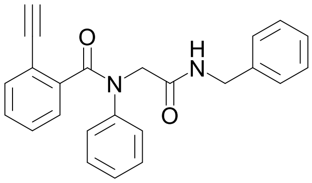

Compound **17c** (93 mg, 0.177 mmol) was dissolved in DCM (2 ml). TBAF (265 μl of 1.0M solution, 0.265 mmol) was then added and the mixture was stirred for 1 hr at room temperature under N_2. To workup, the reaction was diluted with EtOAc and then the organic layer was washed with H_2O, brine, dried over sodium sulfate, and evaporated under vacuum. Flash column chromatography using a gradient of 20-50% EtOAc in Hex was used to purify the desired compound. Yield: 44 mg, 47%. R_f =0.73 (EtOAc/Hex, 1:1). **^{1}H NMR** ($CDCl_3$, 300 MHz) δ 7.39-7.28 (m, 6H), 7.20-7.08 (m, 8H), 4.61 (s, 2H), 4.53 (d, *J* = 5.7 Hz, 2H), 2.62 (s, 1H); **^{13}C NMR** ($CDCl_3$, 75 MHz): δ 170.7, 168.6, 144.2, 143.7, 142.4, 139.3, 138.2, 133.1, 129.4, 129.1, 129.0, 128.7, 128.4, 127.9, 127.3(2), 81.7, 81.6, 54.6, 43.9. **HRMS** for $C_{24}H_{20}N_2O_2$ $[M+Na]^+$ calcd. 391.1417, found 391.1421.

Preparation of **1d** :

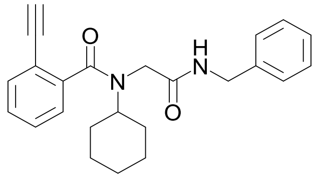

Compound **17d** (193 mg, 0.364 mmol) was dissolved in DCM (2 ml). TBAF (546 μl of 1.0M solution, 0.546 mmol) was then added and the mixture was stirred for 1 hr at room temperature under N_2. To workup, the reaction was diluted with EtOAc and then the organic layer was washed with H_2O, brine, dried over sodium sulfate, and evaporated under vacuum. Flash column chromatography using a gradient of 20-50% EtOAc in Hex was used to purify the desired compound. Yield: 124 mg, 90%. R_f =0.31 (EtOAc/Hex, 1:1). **^{1}H NMR** ($CDCl_3$, 300 MHz) δ 7.48-7.43 (m, 1H), 7.40-7.25 (m, 7H), 7.19-7.16 (m, 1H), 7.08 (br s, 1H), 4.70-4.50 (m, 2H), 4.40-4.24 (m, 1H), 3.88-3.72 (m, 1H), 3.27-3.17 (m, 1H), 2.18 (s, 1H), 1.93-1.80 (m, 1H), 1.77-1.60(m, 2H), 1.58-1.37 (m, 4H), 1.08-0.85 (m, 3H); **^{13}C NMR** ($CDCl_3$, 75 MHz): δ 171.3, 169.8, 139.5, 138.4, 133.2, 129.5, 129.1, 128.9, 128.4, 127.8, 125.6, 118.7, 81.2, 81.1, 59.4, 46.1, 43.7, 31.2, 30.8, 25.5, 25.4, 24.8. **HRMS** for $C_{24}H_{26}N_2O_2$ $[M+H]^+$ calcd. 375.2067, found 375.2060.

Preparation of **1e** :

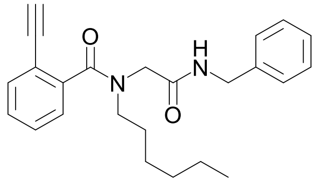

Compound **17e** (213 mg, 0.400 mmol) was dissolved in DCM (2 ml). TBAF (655 μl of 1.0M solution, 0.655 mmol) was then added and the mixture was stirred for 1 hr at room temperature under N_2. To workup, the reaction was diluted with EtOAc and then the organic layer was washed with H_2O, brine, dried over sodium sulfate, and evaporated under vacuum. Flash column chromatography using a gradient of 25-50% EtOAc in Hex was used to purify the desired compound. Yield: 130 mg, 86%. R_f =0.35 (EtOAc/Hex, X:X). **^{1}H NMR** ($CDCl_3$, 300 MHz) δ 7.51-7.44 (m, 1H), 7.41-7.27 (m, 7H), 7.09-7.00 (m, 1H), 4.48 (s, 2H), 3.17 (t, *J*= 7.8, 2H), 2.35 (s, 1H), 1.81-1.72 (m, 1H), 1.54-1.40 (m, 2H), 1.32-1.00 (m, 7H), 0.80 (t, *J*= 6.8, 3H); **^{13}C NMR** ($CDCl_3$, 75 MHz): δ 171.1, 169.0, 138.9, 138.2, 133.2, 129.5, 129.3, 129.0, 128.3, 127.8, 126.5, 118.9, 81.3, 81.2, 50.5, 49.9, 43.7, 31.1, 28.2, 26.0, 22.4, 13.9. **HRMS** for $C_{24}H_{28}N_2O_2$ $[M+H]^+$ calcd. 377.2224, found 377.2230.

Preparation of **1f** :

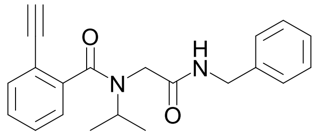

Compound **17f** (231 mg, 0.472 mmol) was dissolved in DCM (2 ml). TBAF (708 μl of 1.0M solution, 0.708 mmol) was then added and the mixture was stirred for 1 hr at room temperature under N_2. To workup, the reaction was diluted with EtOAc and then the organic layer was washed with H_2O, brine, dried over sodium sulfate, and evaporated under vacuum. Flash column chromatography using a gradient of 50-75% EtOAc in Hex was used to purify the desired compound. Yield: 156 mg, 98%. R_f =0.57 (EtOAc/Hex, 3:4). **^{1}H NMR** ($CDCl_3$, 300 MHz) δ 7.49-7.11 (m, 9H), 4.68-4.27 (m, 3H), 3.83-3.72 (m, 2H), 2.19 (s, 1H), 1.24 (s, 3H), 1.07 (s, 3H); **^{13}C NMR** ($CDCl_3$, 75 MHz): δ 171.1, 169.7, 139.4, 138.4, 133.3, 129.6, 129.2, 128.9, 128.5, 127.8, 125.7, 118.8, 81.2, 51.1, 45.0, 43.7, 20.7. **HRMS** for $C_{21}H_{22}N_2O_2$ $[M+Na]^+$ calcd. 357.1573, found 357.1565.

Preparation of **1g** :

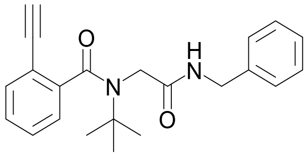

Compound **17g** (26 mg, 0.051 mmol) was dissolved in DCM (2 ml). TBAF (76 µl of 1.0M solution, 0.076 mmol) was then added and the mixture was stirred for 1 hr at room temperature under N_2. To workup, the reaction was diluted with EtOAc and then the organic layer was washed with H_2O, brine, dried over sodium sulfate, and evaporated under vacuum. Flash column chromatography using a gradient of 30-50% EtOAc in Hex was used to purify the desired compound. Yield: 18 mg, 99%. R_f =0.41 (EtOAc/Hex, 1:1). ^{1}**H NMR** ($CDCl_3$, 300 MHz) δ 7.48-7.43 (m, 1H), 7.37-7.19 (m, 7H), 7.16-7.13 (m, 1H), 4.40 (s, 2H), 3.95 (s, 2H), 2.92 (s, 1H), 1.50 (s, 9H); ^{13}**C NMR** ($CDCl_3$, 75 MHz): δ 171.7, 169.8, 137.8, 133.4, 129.5, 129.0(2), 128.7, 128.3(2), 128.0, 125.6, 81.4, 58.8, 51.1, 50.1, 43.8, 28.5. **HRMS** for $C_{22}H_{24}N_2O_2$ $[M+Na]^+$ calcd. 371.1730, found 371.1725.

Preparation of **1h** :

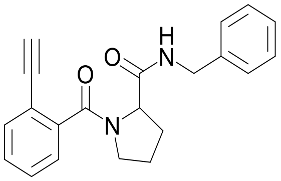

Compound **17h** (213 mg, 0.437 mmol) was dissolved in DCM (2 ml). TBAF (655 µl of 1.0M solution, 0.655 mmol) was then added and the mixture was stirred for 1 hr at room temperature under N_2. To workup, the reaction was diluted with EtOAc and then the organic layer was washed with H_2O, brine, dried over sodium sulfate, and evaporated under vacuum. Flash column chromatography using a gradient of 50-75% EtOAc in Hex was used to purify the desired compound. Yield: 133 mg, 91%. R_f =0.39 (EtOAc/Hex, 3:4). ^{1}**H NMR** ($CDCl_3$, 300 MHz) δ 7.50-7.22 (m, 9H), 4.93-4.82 (m, 1H), 4.62-4.33 (m, 2H), 3.52-3.42 (m, 1H), 3.27-3.13 (m, 1H), 2.61-2.44 (m, 2H), 2.16-1.81 (m, 4H); ^{13}**C NMR** ($CDCl_3$, 75 MHz): δ 171.0, 170.0, 140.0, 138.4, 133.2, 129.6, 129.4, 128.9, 128.1, 127.6, 126.4, 118.8, 81.2, 80.8, 60.1, 49.0, 43.7, 28.2, 24.9. **HRMS** for $C_{21}H_{20}N_2O_2$ $[M+H]^+$ calcd. 333.1598, found 333.1591.

Preparation of **1i** :

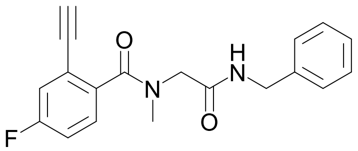

Compound **17i** (167 mg, 0.347 mmol) was dissolved in DCM (2 ml). TBAF (521 µl of 1.0M solution, 0.521 mmol) was then added and the mixture was stirred for 1 hr at room temperature under N_2. To workup, the reaction was diluted with EtOAc and then the organic layer was washed with H_2O, brine, dried over sodium sulfate, and evaporated under vacuum. Flash column chromatography using a gradient of 50-75% EtOAc in Hex was used to purify the desired compound. Yield: 87 mg, 77%. R_f =0.14 (EtOAc/Hex, 1:1). ^{1}**H NMR** ($CDCl_3$, 300 MHz, mixture of rotamers) δ 7.38-7.28 (m, 6H), 7.23-7.10 (m, 2H), 6.79 (br s, 1H), 4.49 (d, *J*= 5.6, 2H), 4.28 (s, 2H), 2.97 (s, 3H), 2.45 (s, 1H); ^{13}**C NMR** ($CDCl_3$, 75 MHz): δ 170.2, 168.3, 161.0, 138.1, 129.1, 128.9, 128.8, 128.3, 128.0, 121.2, 120.2, 119.9, 117.5, 117.2, 82.1, 80.0, 52.2, 43.7, 38.0. **HRMS** for $C_{19}H_{17}FN_2O_2$ $[M+Na]^+$ calcd. 347.1166, found 347.1176.

Preparation of **1j** :

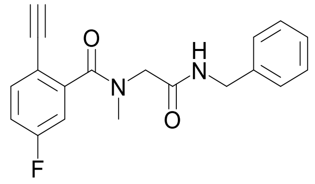

Compound **17j** (200 mg, 0.417 mmol) was dissolved in DCM (2 ml). TBAF (630 µl of 1.0M solution, 0.626 mmol) was then added and the mixture was stirred for 1 hr at room temperature under N_2. To workup, the reaction was diluted with EtOAc and then the organic layer was washed with H_2O, brine, dried over sodium sulfate, and evaporated under vacuum. Flash column chromatography using a gradient of 50-75% EtOAc in Hex was used to purify the desired compound. Yield: 117 mg, 87%. R_f =0.20 (EtOAc/Hex, 1:1). **^{1}H NMR** ($CDCl_3$, 300 MHz, mixture of rotamers) δ 7.51-7.46 (m, 1H), 7.38-7.29 (m, 6H), 7.11-6.99 (m, 2H), 4.49 (d, *J*= 5.6, 2H), 4.27 (s, 2H), 2.98 (s, 3H), 2.40 (s, 1H); **^{13}C NMR** ($CDCl_3$, 75 MHz): δ 169.5, 168.1, 164.6, 138.0, 135.5, 135.4, 129.1, 128.3, 128.1, 128.0, 117.1, 116.8, 114.4, 114.1, 80.8, 80.2, 52.1, 43.7, 37.8. **HRMS** for $C_{19}H_{17}FN_2O_2$ $[M+Na]^+$ calcd. 347.1166, found 347.1172.

Preparation of **1k** :

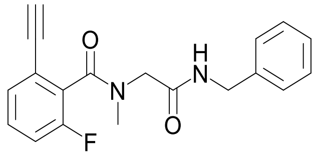

Compound **17k** (181 mg, 0.376 mmol) was dissolved in DCM (2 ml). TBAF (565 µl of 1.0M solution, 0.565 mmol) was then added and the mixture was stirred for 1 hr at room temperature under N_2. To workup, the reaction was diluted with EtOAc and then the organic layer was washed with H_2O, brine, dried over sodium sulfate, and evaporated under vacuum. Flash column chromatography using a gradient of 50-75% EtOAc in Hex was used to purify the desired compound. Yield: 102 mg, 84%. R_f =0.21 (EtOAc/Hex, 1:1). **^{1}H NMR** ($CDCl_3$, 300 MHz, mixture of rotamers) δ 7.39-7.29 (m, 8H), 7.19-7.09 (m, 1H), 4.69-4.63 (m, 1H), 4.52-4.48 (m, 2H), 4.00-3.95 (m, 1H), 2.98 (s, 3H), 2.51 (s, 1H); **^{13}C NMR** ($CDCl_3$, 75 MHz): δ 168.2, 159.8, 138.0, 131.1, 130.9, 129.2, 129.0, 128.2, 127.9, 117.2, 117.0, 82.0, 79.8, 52.0, 43.8, 37.3, 34.0. **HRMS** for $C_{19}H_{17}FN_2O_2$ $[M+Na]^+$ calcd. 347.1166, found 347.1169.

Preparation of **1l** :

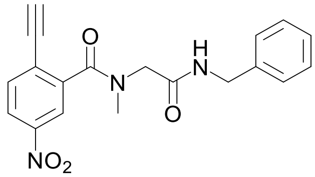

Compound **17l** (207 mg, 0.408 mmol) was dissolved in DCM (2 ml). TBAF (612 µl of 1.0M solution, 0.612 mmol) was then added and the mixture was stirred for 1 hr at room temperature under N_2. To workup, the reaction was diluted with EtOAc and then the organic layer was washed with H_2O, brine, dried over sodium sulfate, and evaporated under vacuum. Flash column chromatography using a gradient of 50-75% EtOAc in Hex was used to purify the desired compound.Yield: 143 mg, quan. R_f =0.44 (EtOAc/Hex, 3:4). **^{1}H NMR** ($CDCl_3$, 300 MHz, mixture of rotamers) δ 8.24-8.18 (m, 2H), 7.70-7.64 (m, 1H), 7.39-7.29 (m, 5H), 6.73 (br s, 1H), 4.49 (d, *J*= 5.6, 2H), 4.28 (s, 2H), 3.00 (s, 3H), 2.75 (s, 1H); **^{13}C NMR** ($CDCl_3$, 75 MHz): δ 168.5, 167.8, 147.8, 140.1, 138.0, 134.3, 129.1, 128.3, 128.0, 125.5, 124.2, 122.2, 86.1, 79.3, 54.6, 52.1, 43.8, 37.9, 34.4. **HRMS** for $C_{19}H_{17}N_3O_4$ $[M+Na]^+$ calcd. 374.1111, found 374.1104.

Preparation of **1m** :

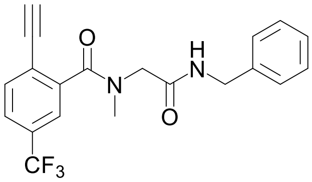

Compound **17m** (84 mg, 0.158 mmol) was dissolved in DCM (2 ml). TBAF (236 µl of 1.0M solution, 0.236 mmol) was then added and the mixture was stirred for 1 hr at room temperature under N_2. To workup, the reaction was diluted with EtOAc and then the organic layer was washed with H_2O, brine, dried over sodium sulfate, and evaporated under vacuum. Flash column chromatography using a gradient of 25-50% EtOAc in Hex was used to purify the desired compound. Yield: 58 mg, quan. R_f =0.19 (EtOAc/Hex, 1:1). **^{1}H NMR** ($CDCl_3$, 300 MHz, mixture of rotamers) δ 7.62-7.57 (m, 3H), 7.38-7.29 (m, 5H), 6.81 (br s, 1H), 4.49 (d, *J*= 5.6, 2H), 4.27 (s, 2H), 2.98 (s, 3H), 2.61 (s, 1H); **^{13}C NMR** ($CDCl_3$, 75 MHz): δ 169.4, 168.0, 167.3, 139.4, 138.0, 133.7, 129.0, 128.2, 127.9, 125.2, 122.7, 83.6, 79.8, 54.6, 52.0, 43.7, 37.9, 34.2. **HRMS** for $C_{20}H_{17}F_3N_2O_2$ $[M+Na]^+$ calcd. 397.1134, found 397.1140.

Preparation of **1n** :

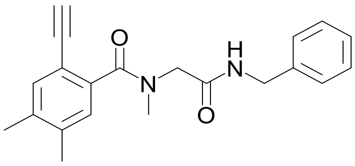

Compound **17n** (243 mg, 0.510 mmol) was dissolved in DCM (2 ml). TBAF (765 µl of 1.0M solution, 0.765 mmol) was then added and the mixture was stirred for 1 hr at room temperature under N_2. To workup, the reaction was diluted with EtOAc and then the organic layer was washed with H_2O, brine, dried over sodium sulfate, and evaporated under vacuum. Flash column chromatography using a gradient of 50-75% EtOAc in Hex was used to purify the desired compound. Yield: 159 mg, 93%. R_f =0.20 (EtOAc/Hex, 1:1). **^{1}H NMR** ($CDCl_3$, 300 MHz, mixture of rotamers) δ 7.37-7.21 (m, 7H), 6.92 (br s, 1H), 4.49 (d, *J*= 5.6, 2H), 4.29 (s, 2H), 2.97 (s, 3H), 2.36 (s, 1H), 2.26 (s, 3H), 2.24 (s, 3H); **^{13}C NMR** ($CDCl_3$, 75 MHz): δ 171.3, 168.6, 168.0, 139.1, 138.4, 138.1, 136.2, 134.0, 129.0, 128.2, 127.8(2), 116.1, 81.6, 79.9, 52.0, 43.6, 37.9, 19.7. **HRMS** for $C_{21}H_{22}N_2O_2$ $[M+Na]^+$ calcd. 357.1573, found 357.1574.

Preparation of **1o** :

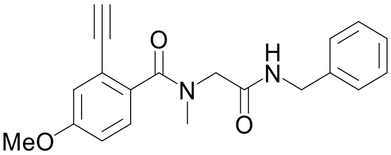

Compound **17o** (208 mg, 0.423 mmol) was dissolved in DCM (2 ml). TBAF (635 µl of 1.0M solution, 0.635 mmol) was then added and the mixture was stirred for 1 hr at room temperature under N_2. To workup, the reaction was diluted with EtOAc and then the organic layer was washed with H_2O, brine, dried over sodium sulfate, and evaporated under vacuum. Flash column chromatography using a gradient of 75-100% EtOAc in Hex was used to purify the desired compound. Yield: 103 mg, 72%. R_f =0.09 (EtOAc/Hex, 1:1). **^{1}H NMR** ($CDCl_3$, 300 MHz, mixture of rotamers) δ 7.37-7.20 (m, 6H), 6.98-6.91 (m, 3H), 4.49 (d, *J*= 5.6, 2H), 4.28 (s, 2H), 3.81 (s, 3H), 2.98 (s, 3H), 2.40 (s, 1H); **^{13}C NMR** ($CDCl_3$, 75 MHz): δ 171.0, 168.6, 160.1, 138.1, 131.1, 129.0, 128.4, 128.3, 127.9, 120.3, 117.8, 116.1, 81.3, 80.8, 55.6, 52.2, 43.6, 38.1. **HRMS** for $C_{20}H_{20}N_2O_3$ $[M+Na]^+$ calcd. 359.1366, found 359.1370.

Preparation of **1p** :

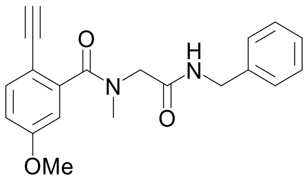

Compound **17p** (178mg, 0.362 mmol) was dissolved in DCM (2 ml). TBAF (550 µl of 1.0M solution, 0.544 mmol) was then added and the mixture was stirred for 1 hr at room temperature under N_2. To workup, the reaction was diluted with EtOAc and then the organic layer was washed with H_2O, brine, dried over sodium sulfate, and evaporated under vacuum. Flash column chromatography using a gradient of 50-75% EtOAc in Hex was used to purify the desired compound. Yield: 121 mg, 99%. R_f =0.10 (EtOAc/Hex, 1:1). **^{1}H NMR** ($CDCl_3$, 300 MHz, mixture of rotamers) δ 7.42-7.28 (m, 6H), 6.92-6.78 (m, 3H), 4.49 (d, *J*= 5.6, 2H), 4.29 (s, 2H), 3.82 (s, 3H), 2.98 (s, 3H), 2.32 (s, 1H); **^{13}C NMR** ($CDCl_3$, 75 MHz): δ 170.7, 168.4, 160.6, 140.2, 138.0, 134.8, 129.0, 128.3, 128.1, 127.9, 115.8, 111.7, 110.8, 81.4, 79.5, 55.7, 52.0, 43.6, 37.8, 33.9. **HRMS** for $C_{20}H_{20}N_2O_3$ $[M+Na]^+$ calcd. 359.1366, found 359.1376.

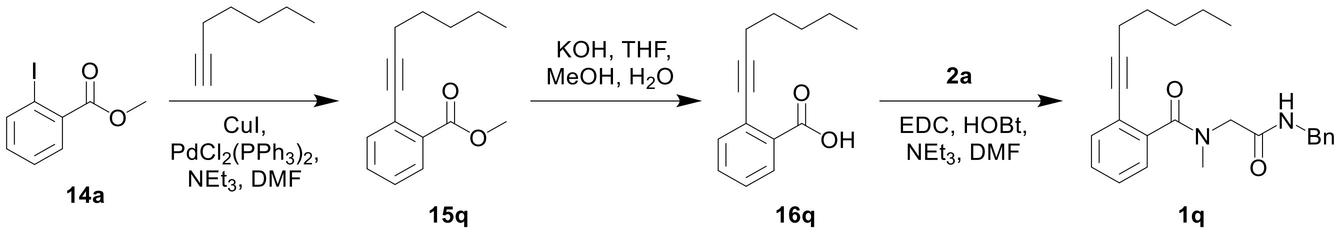

Scheme S6. Synthesis of **1q**

Preparation of **15q** :

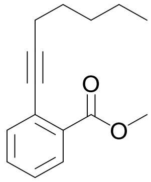

Compound **14a** (520 mg, 1.99 mmol), CuI (76 mg, 0.397 mmol), and $PdCl_2(PPh_3)_2$ (140 mg, 0.199 mmol) were dissolved in DMF (2 ml) and NEt_3 (2 ml). The mixture was degassed with N_2 for 5 min. 1-Heptyne (521 µl, 3.97 mmol) was then added and the mixture was stirred for 16 hr at 30°C under N_2. To workup, the reaction was diluted with EtOAc and then the organic layer was washed with H_2O, brine, dried over sodium sulfate, and evaporated under vacuum. Flash column chromatography using a gradient of 10-20% EtOAc in Hex was used to purify the desired compound. Yield: 413 mg, 90%. R_f =0.63 (EtOAc/Hex, 1:4). **^{1}H NMR** ($CDCl_3$, 300 MHz) δ 7.88 (d, *J*= 7.9, 1H), 7.52-7.28 (m, 3H), 3.92 (s, 3H), 2.47 (t, *J*= 7.0, 2H), 1.69-1.60 (m, 2H), 1.51-1.31 (m, 4H), 0.93 (t, *J* = 7.2, 3H); **^{13}C NMR** ($CDCl_3$, 75 MHz): δ 167.3, 134.4, 132.2, 131.7, 130.3, 127.3, 124.7, 96.2, 79.3, 52.1, 31.2, 28.5, 22.3, 19.8, 14.0. **HRMS** for $C_{15}H_{18}O_2$ $[M+H]^+$ calcd. 231.1380, found 231.1385.

Preparation of **16q** :

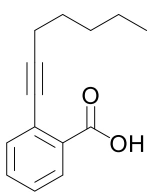

Compound **15q** (457 mg, 1.99 mmol) was dissolved in MeOH (6 ml) and THF (6 ml) at 0°C. KOH (1110 mg, 19.85 mmol) dissolved in H_2O (6 ml) was then added and the mixture was stirred for 4.5 hr at rt under N_2. To workup, the mixture was acidified to pH~3 with 1M HCl solution, which allowed the product to be extracted with EtOAc. The organic layer was washed with H_2O, brine, dried over sodium sulfate, and evaporated under vacuum. Flash column chromatography using a gradient of 0-50% EtOAc in Hex was used to purify the desired compound. Yield: 347 mg, 80%. R_f =0.53 (EtOAc/Hex, 1:1). **^{1}H NMR** ($CDCl_3$, 300 MHz) δ 8.08 (d, *J*= 7.9, 1H), 7.56-7.34 (m, 3H), 2.50 (t, *J*= 7.0, 2H), 1.71-1.61 (m, 2H), 1.52-1.31 (m, 4H), 0.93 (t, *J* = 7.1, 3H); **^{13}C NMR** ($CDCl_3$, 75 MHz): δ 170.4, 134.5, 132.6, 131.5, 130.9, 127.7, 124.7, 98.3, 79.1, 31.2, 28.2, 22.3, 19.8, 14.0. **HRMS** for $C_{14}H_{16}O_2$ $[M+H]^+$ calcd. 217.1223, found 217.1224.

Preparation of **1q** :

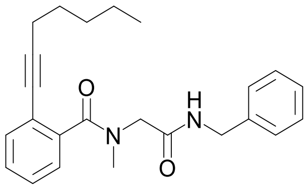

Compound **16q** (134 mg, 0.620 mmol), **2a** (144 mg, 0.806 mmol), EDC (192 mg, 1.24 mmol), HOBt (167 mg, 1.24 mmol) were dissolved in DMF (5 ml). NEt_3 (347 µl, 2.48 mmol) was then added and the mixture was stirred for 16 hr at room temperature under N_2. To workup, the reaction was diluted with EtOAc and then the organic layer was washed with H_2O, brine, dried over sodium sulfate, and evaporated under vacuum. Flash column chromatography using a gradient of 25-50% EtOAc in Hex was used to purify the desired compound. Yield: 181 mg, 78%. R_f =0.26 (EtOAc/Hex, 1:1). **^{1}H NMR** ($CDCl_3$, 300 MHz, mixture of rotamers) δ 7.45-7.18 (m, 9H), 7.01 (br s, 1H), 4.51 (d, *J*= 5.9, 2H), 4.24 (s, 2H), 3.00 (s, 3H), 2.21 (t, *J*= 7.1, 2H), 1.55-1.45 (m, 2H), 1.37-1.24 (m, 4H), 0.90 (t, *J*= 7.0, 3H); **^{13}C NMR** ($CDCl_3$, 75 MHz): δ 171.7, 168.9, 138.3, 137.9, 132.8, 129.4, 128.9, 128.3, 127.9, 127.8, 127.6, 127.4, 126.7, 121.1, 94.7, 52.5, 43.5, 38.1, 34.0, 31.1, 28.4, 22.2, 19.3, 14.0. **HRMS** for $C_{24}H_{28}N_2O_2$ $[M+H]^+$ calcd. 377.2224, found 377.2227.

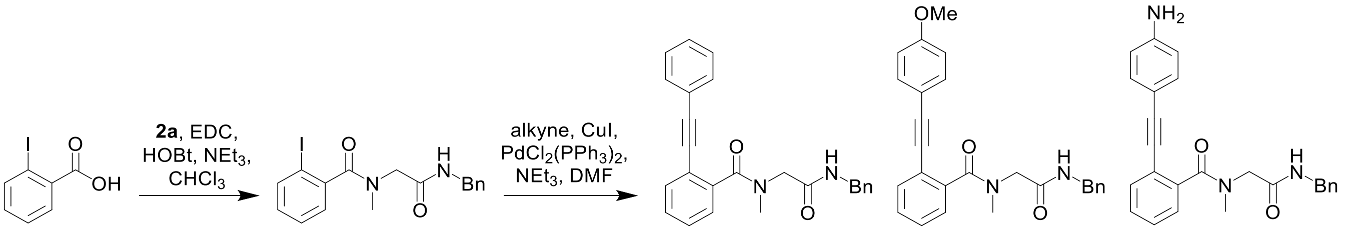

Scheme S7. Synthesis of **1r-t**

Preparation of **18** :

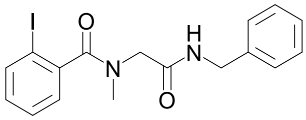

2-Iodobenzoic acid **13a** (320 mg, 1.26 mmol), **2a** (225 mg, 1.26 mmol), EDC (391 mg, 2.53 mmol), HOBt (341 mg, 2.53 mmol) were dissolved in $CHCl_3$ (20 ml). NEt_3 (353 µl, 2.53 mmol) was then added and the mixture was stirred for 16 hr at 30°C under N_2. To workup, the reaction was diluted with EtOAc and then the organic layer was washed with H_2O, brine, dried over sodium sulfate, and evaporated under vacuum. Flash column chromatography using a gradient of 50-75% EtOAc in Hex was used to purify the desired compound. Yield: 383 mg, 74%. R_f =0.11 (EtOAc/Hex, 1:1). **^{1}H NMR** ($CDCl_3$, 300 MHz, mixture of rotamers) δ 7.78 (d, *J*= 8.0, 1H), 7.43-7.06 (m, 8H), 6.94 (br s, 1H), 4.48 (s, 2H), 3.85 (s, 2H), 2.91 (s, 3H); **^{13}C NMR** ($CDCl_3$, 75 MHz): δ 171.8, 168.2, 141.6, 139.3, 137.9, 130.8, 129.0, 128.8, 128.5, 127.9, 127.3, 92.3, 52.1, 43.9, 37.9. **HRMS** for $C_{17}H_{17}IN_2O_2$ $[M+Na]^+$ calcd. 431.0227, found 431.0230.

Preparation of **1r** :

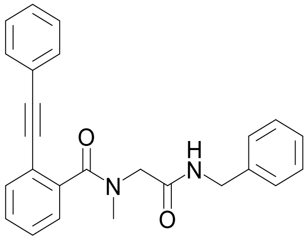

Compound **18** (98 mg, 0.240 mmol), CuI (9 mg, 0.048 mmol), and $PdCl_2(PPh_3)_2$ (17 mg, 0.024 mmol) were dissolved in DMF (2 ml) and NEt_3 (2 ml). The mixture was degassed with N_2 for 5 min. Phenylacetylene (53 µl, 0.480 mmol) was then added and the mixture was stirred for 16 hr at 30°C under N_2. To workup, the reaction was diluted with EtOAc and then the organic layer was washed with H_2O, brine, dried over sodium sulfate, and evaporated under vacuum. Flash column chromatography using a gradient of 10-50% EtOAc in Hex was used to purify the desired compound. Yield: 88 mg, 95%. R_f =0.21 (EtOAc/Hex, 1:1). **^{1}H NMR** ($CDCl_3$, 300 MHz, mixture of rotamers) δ 7.59-7.04 (m, 14H), 6.96 (br s, 1H), 4.28 (s, 2H), 4.20 (d, *J*= 5.6, 2H), 3.06 (s, 3H); **^{13}C NMR** ($CDCl_3$, 75 MHz): δ 171.4, 168.8, 138.2(2), 132.6, 131.9, 129.6, 129.2, 129.1, 128.8(2), 127.8, 127.4, 126.9, 122.4, 120.3, 93.0, 86.5, 52.5, 43.2, 38.1. **HRMS** for $C_{25}H_{22}N_2O_2$ $[M+Na]^+$ calcd. 405.1573, found 405.1577.

Preparation of **1s** :

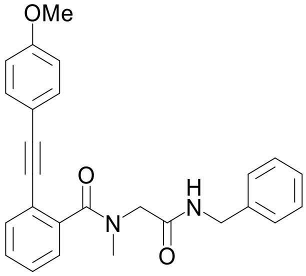

Compound **18** (172 mg, 0.422 mmol), CuI (16 mg, 0.084 mmol), and $PdCl_2(PPh_3)_2$ (30 mg, 0.042 mmol) were dissolved in DMF (2 ml) and NEt_3 (2 ml). The mixture was degassed with N_2 for 5 min. 4-Ethynylanisole (109 µl, 0.843 mmol) was then added and the mixture was stirred for 16 hr at 30°C under N_2. To workup, the reaction was diluted with EtOAc and then the organic layer was washed with H_2O, brine, dried over sodium sulfate, and evaporated under vacuum. Flash column chromatography using a gradient of 20-40% EtOAc in Hex was used to purify the desired compound. Yield: 45 mg, 26%. R_f =0.21 (EtOAc/Hex, 1:1). **^{1}H NMR** ($CDCl_3$, 300 MHz, mixture of rotamers) δ 7.71-7.11 (m, 13H), 6.83 (d, *J*= 8.8, 2H), 4.29 (s, 2H), 4.21 (d, *J*= 5.6, 2H), 3.82 (s, 3H), 3.06 (s, 3H); **^{13}C NMR** ($CDCl_3$, 75 MHz): δ 171.5, 168.8, 160.4, 138.3, 138.0, 133.4, 132.5, 132.4, 132.2(3), 129.6, 128.8, 128.7, 128.6, 127.4(2), 126.8, 120.7, 114.4, 93.2, 85.4, 55.4, 52.4, 43.2, 38.1. **HRMS** for $C_{26}H_{24}N_2O_3$ $[M+H]^+$ calcd. 413.1860, found 413.1866.

Preparation of **1t** :

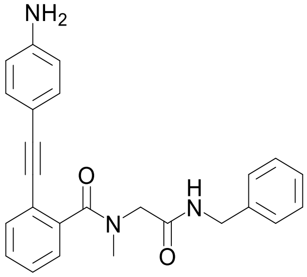

Compound **18** (81 mg, 0.199 mmol), CuI (8 mg, 0.040 mmol), and $PdCl_2(PPh_3)_2$ (14 mg, 0.020 mmol) were dissolved in DMF (2 ml) and NEt_3 (2 ml). The mixture was degassed with N_2 for 5 min. 4-Ethynylaniline (47 mg, 0.397 mmol) dissolved in DMF (0.5 ml) was then added and the mixture was stirred for 16 hr at 30°C under N_2. To workup, the reaction was diluted with EtOAc and then the organic layer was washed with H_2O, brine, dried over sodium sulfate, and evaporated under vacuum. Flash column chromatography using a gradient of 50-75% EtOAc in Hex was used to purify the desired compound. Yield: 80 mg, quan. R_f =0.33 (EtOAc/Hex, 3:4). **^{1}H NMR** ($CDCl_3$, 300 MHz, mixture of rotamers) δ 7.55-6.98 (m, 13H), 6.58 (d, *J*= 8.5, 2H), 6.53 (br s, 1H), 4.29 (s, 2H), 4.20 (s, 2H), 3.05 (s, 3H); **^{13}C NMR** ($CDCl_3$, 75 MHz): δ 171.6, 168.9, 147.6, 138.4, 137.8, 133.3, 132.4, 129.5, 128.7, 128.5, 127.4(2), 126.8, 121.0, 114.9, 111.4, 94.1, 84.6, 52.4, 43.2, 38.1. **HRMS** for $C_{25}H_{23}N_3O_2$ $[M+H]^+$ calcd. 398.1863, found 398.1867.

1.5.2 Endoxifen Derivatives

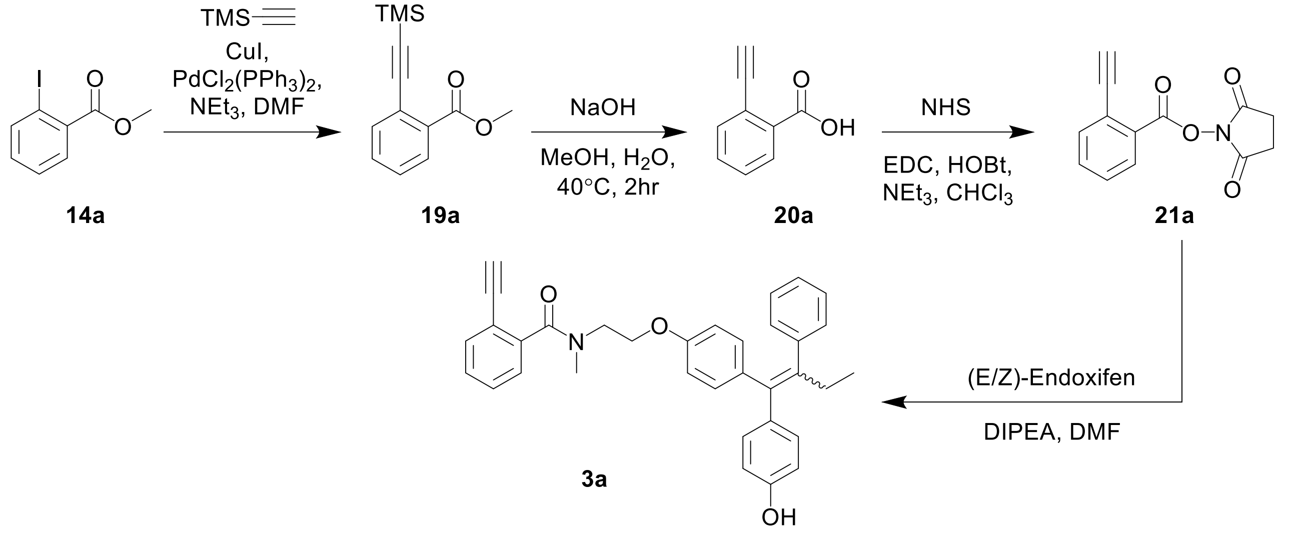

Scheme S8. Synthesis of **3a**

Preparation of **19a** :

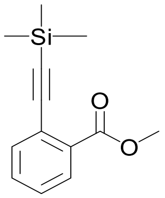

Compound **14a** (405 mg, 1.55 mmol), CuI (59 mg, 0.31 mmol), and $PdCl_2(PPh_3)_2$ (108 mg, 0.15 mmol) were dissolved in DMF (2 ml) and NEt_3 (2 ml). The mixture was degassed with N_2 for 5 min. (Trimethylsilyl)acetylene (430 μl, 3.09 mmol) was then added and the mixture was stirred for 16 hr at 30°C under N_2. To workup, the reaction was diluted with EtOAc and then the organic layer was washed with H_2O, brine, dried over sodium sulfate, and evaporated under vacuum. Flash column chromatography using a gradient of 5-10% EtOAc in Hex was used to purify the desired compound. Yield: 346 mg, 96%. R_f =0.41 (EtOAc/Hex, 1:10). **^{1}H NMR** ($CDCl_3$, 300 MHz) δ 7.91 (d, *J* = 7.7 Hz, 1H), 7.59 (d, *J* = 7.5 Hz, 1H), 7.47-7.34 (m, 2H), 3.93 (s, 3H), 0.28 (s, 9H); **^{13}C NMR** ($CDCl_3$, 75 MHz): δ 167.2, 134.8, 132.8, 131.7, 130.5, 128.4, 123.4, 103.5, 99.8, 52.1, 0.1.
HRMS for $C_{13}H_{16}O_2Si$ $[M+H]^+$ calcd. 233.0992, found 233.0992.

Preparation of **20a** :

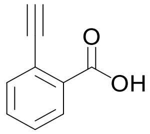

Compound **19a** (366 mg, 1.58 mmol) was dissolved in MeOH (5 ml), followed by the addition of aqueous NaOH (5 ml of 2.0M solution). The mixture was then stirred for 2 hr at 40°C. To workup, the H_2O layer was washed with EtOAc (1×) and acidified to pH~1 with 1M HCl, which allowed the product to be extracted with EtOAc. The organic layer was then washed with H_2O, brine, dried over sodium sulfate, and evaporated under vacuum. Flash column chromatography using a gradient of 30-50% EtOAc in Hex was used to purify the desired compound. Yield: 165 mg, 48%. R_f =0.14 (EtOAc/Hex, 1:1). Characterization matched a previous report of this known compound.[13] **^{1}H NMR** ($CDCl_3$, 300 MHz) δ 11.69 (br s, 1H), 8.10 (d, *J* = 7.8 Hz, 1H), 7.67 (d, *J* = 7.7 Hz, 1H), 7.55 (td, *J* = 1.4, 7.5 Hz, 1H), 7.46 (td, *J* = 1.4, 7.7 Hz, 1H), 3.46 (s, 1H).

Preparation of **21a** :

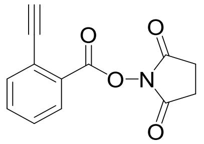

Compound **20a** (27 mg, 0.185 mmol), *N*-hydroxysuccinimide (32 mg, 0.277 mmol), EDC (71 mg, 0.370 mmol), HOBt (50 mg, 0.370 mmol) were dissolved in $CHCl_3$ (5 ml). NEt_3 (126 µl, 0.740 mmol) was then added and the mixture was stirred for 16 hr at room temperature under N_2. To workup, the solvent was evaporated under vacuum and the mixture redissolved in EtOAc. The organic layer was then washed with H_2O, brine, dried over sodium sulfate, and evaporated under vacuum. Flash column chromatography using a gradient of 0-50% EtOAc in Hex was used to purify the desired compound. Yield: 39 mg, 87%. R_f =0.40 (EtOAc/Hex, 1:1). ^{1}**H NMR** ($CDCl_3$, 300 MHz) δ 8.16 (d, *J* = 8.2 Hz, 1H), 7.70-7.57 (m, 2H), 7.47 (t, *J* = 7.7 Hz, 1H), 3.45 (s, 1H), 2.89 (s, 4H); ^{13}**C NMR** ($CDCl_3$, 75 MHz): δ 169.4, 160.9, 135.5, 133.9, 131.3, 128.9, 127.1, 124.6, 84.3, 80.9, 25.7.
HRMS for $C_{13}H_9NO_4$ $[M+Na]^+$ calcd. 266.0424, found 266.0421.

Preparation of **3a** :

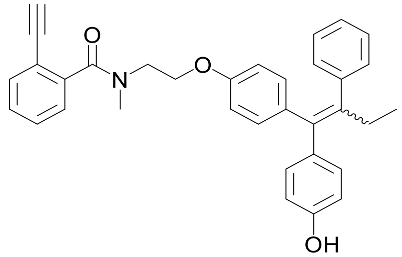

Compound **21a** (6.3 mg, 0.026 mmol) and (E/Z)-Endoxifen (4.7 mg, 0.019 mmol) were dissolved in DMF (300 µl). DIPEA (8 µl, 0.043 mmol) was then added and the mixture was stirred for 16 hr at room temperature. To workup, additional DMF (1 ml) was added. The reaction was then filtered and directly purified by reverse phase HPLC using method 6 (retention time ~27.4 min). Yield: 4.1 mg, 42%. ^{1}**H NMR** ($CDCl_3$, 400 MHz, mixture of rotamers) δ 7.54-7.46 (m, 1H), 7.41-7.26 (m, 3H), 7.17-7.05 (m, 7H), 6.88 (d, *J* = 8.7 Hz, 1H), 6.81-6.67 (m, 3H), 6.53 (d, *J* = 8.8 Hz, 1H), 6.48-6.40 (m, 2H), 4.31 (t, *J* = 5.0 Hz, 1H), 4.15 (t, *J* = 4.9 Hz, 1H), 3.77-3.71 (m, 2H), 3.07 (s, 3H), 2.55 (s, 1H), 2.50-2.42 (m, 2H), 0.92 (t, *J* = 7.4 Hz, 3H); ^{13}**C NMR** ($CDCl_3$, 75 MHz): δ 170.5, 157.6, 156.8, 154.7, 153.8, 142.9, 141.5, 140.3, 138.0, 136.4, 136.1, 133.1, 132.9, 132.3, 132.2, 130.9, 129.9, 129.4, 129.0, 128.1, 126.4, 126.2, 119.2, 115.2, 114.5, 114.2, 113.4, 81.0, 80.7, 68.1, 47.7, 30.4, 29.1, 13.7. **HRMS** for $C_{34}H_{31}NO_3$ $[M+H]^+$ calcd. 502.2377, found 502.2389.

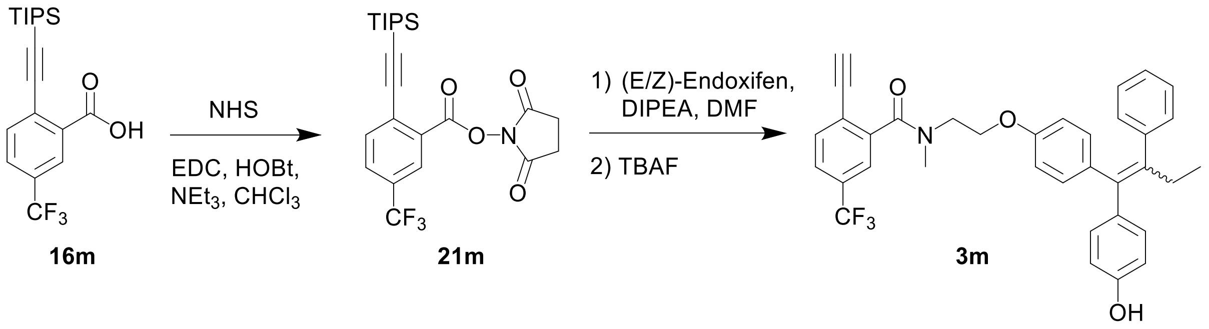

Scheme S9. Synthesis of **3m**

Preparation of **21m** :

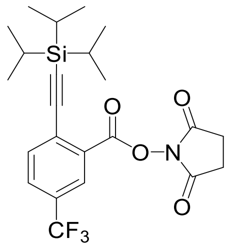

Compound **16m** (170 mg, 0.459 mmol), *N*-hydroxysuccinimide (79 mg, 0.689 mmol), EDC (176 mg, 0.918 mmol), HOBt (124 mg, 0.918 mmol) were dissolved in $CHCl_3$ (5 ml). NEt_3 (312 µl, 1.837 mmol) was then added and the mixture was stirred for 16 hr at room temperature under N_2. To workup, the solvent was evaporated under vacuum and the mixture redissolved in EtOAc. The organic layer was then washed with H_2O, brine, dried over sodium sulfate, and evaporated under vacuum. Flash column chromatography using a gradient of 15-25% EtOAc in Hex was used to purify the desired compound. Yield: 78 mg, 37%. R_f =0.31 (EtOAc/Hex, 1:4). **^{1}H NMR** ($CDCl_3$, 300 MHz) δ 8.35 (s, 1H), 7.82-7.73 (m, 2H), 2.91 (s, 4H), 1.12 (s, 21H); **^{13}C NMR** ($CDCl_3$, 75 MHz): δ 169.1, 159.8, 136.0. 130.1, 130.0, 129.2, 128.3, 127.6, 121.5, 103.4, 102.5, 25.7, 18.6, 11.2. **HRMS** for $C_{23}H_{28}F_3NO_4Si$ $[M+Na]^+$ calcd. 490.1632, found 490.1634.

Preparation of **3m** :

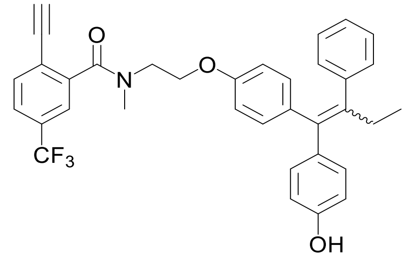

Compound **21m** (6.2 mg, 0.013 mmol) and (E/Z)-Endoxifen (2.5 mg, 0.007 mmol) were dissolved in DMF (300 µl), followed by the addition of DIPEA (3 µl, 0.013 mmol). After stirring for 16 hr at room temperature, TBAF (400 µl of 1M solution in THF) was added to the mixture, which was allowed to further stir for 1 hr. To workup, additional DMF (1 ml) was added. The reaction was then filtered and directly purified by reverse phase HPLC using method 7 (retention time ~29.9 min). Yield: 3.3 mg, 87%. **^{1}H NMR** ($CDCl_3$, 400 MHz, mixture of rotamers) δ 7.66-7.54 (m, 3H), 7.18-7.05 (m, 7H), 6.89 (d, *J* = 8.6 Hz, 1H), 6.83-6.68 (m, 3H), 6.56-6.43 (m, 2H), 4.32 (t, *J* = 4.9 Hz, 1H), 4.16 (t, *J* = 4.7 Hz, 1H), 3.94-3.79 (m, 2H), 3.01 (s, 3H), 2.62 (s, 1H), 2.51-2.42 (m, 2H), 0.95-0.88 (m, 3H); **^{13}C NMR** ($CDCl_3$, 75 MHz): δ 168.9, 157.5, 156.7, 155.1, 154.0, 142.8, 141.4, 140.8, 138.1, 137.9, 136.9, 136.6, 136.0, 133.6, 133.3, 132.3, 132.2, 130.9, 129.9, 128.1, 126.2, 125.8, 123.5, 123.0, 115.3, 114.6, 114.1, 113.4, 83.5, 83.3, 66.7, 47.8, 38.9, 29.1, 13.6. **HRMS** for $C_{35}H_{30}F_3NO_3$ $[M+H]^+$ calcd. 570.2251, found 570.2232.

1.5.3 *Doxorubicin Derivatives*

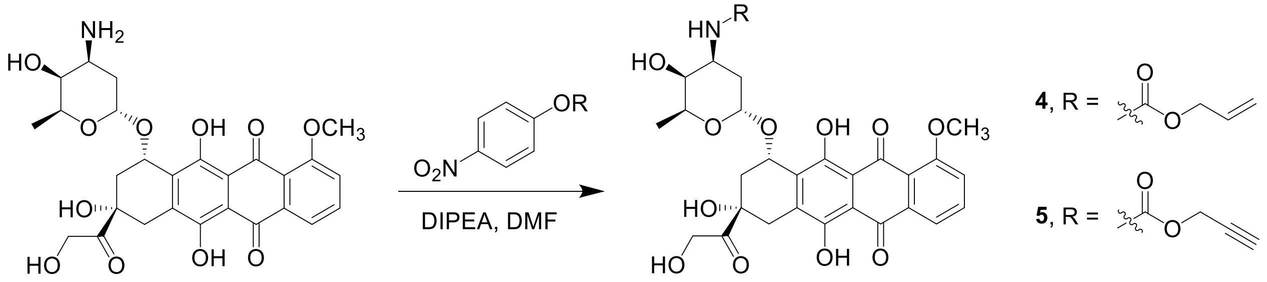

Scheme S10. Synthesis of **4** and **5**

Preparation of **4** :

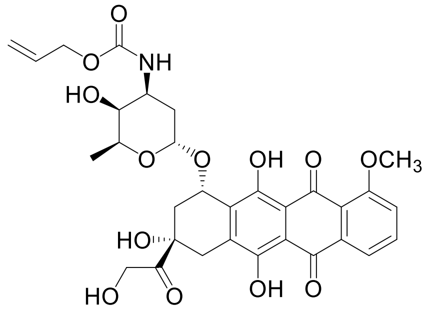

O-(4-Nitrophenyl)-O'-allylcarbonate (18.1 mg, 0.081 mmol), which was prepared according to literature,[14] was added to Doxorubicin hydrochloride (47 mg, 0.081 mmol) dissolved in DMF (1 ml). DIPEA (28 µl, 0.162 mmol) was then added and the mixture was stirred for 16 hr at room temperature. To workup, additional DMF (1 ml) was added. The reaction was then filtered and directly purified by reverse phase HPLC using method 8 (retention time ~20.8 min). Yield: 20.6 mg, 40%. Characterization matched a previous report of this known compound.[15] **^{1}H NMR** (Acetone-d6, 400 MHz) δ 8.02 (dd, *J* = 7.7, 1.1 Hz, 1H), 7.95-7.91 (m, 1H), 7.67 (d, *J* = 8.4 Hz, 1H), 6.00-5.83 (m, 2H), 5.47 (d, *J* = 3.7 Hz, 1H), 5.29-5.22 (m, 2H), 5.11 (d, *J* = 9.9 Hz, 1H), 4.91 (s, 1H), 4.77-4.73 (m, 2H), 4.44 (d, *J* = 5.4 Hz, 2H), 4.29-4.24 (m, 1H), 4.07 (s, 3H), 4.05-3.98 (m, 1H), 3.90-3.83 (m, 1H), 3.67-3.63 (m, 2H), 3.21 (d, *J* = 18.8 Hz, 1H), 3.06 (d, *J* = 18.7 Hz, 1H), 2.53-2.46 (m, 1H), 2.27-2.22 (m, 1H), 2.02-1.99 (m, 1H), 1.78-1.71 (m, 1H), 1.28 (d, *J* = 6.6 Hz, 3H). **LRMS** for $C_{31}H_{33}NO_{13}$ $[M+Na]^+$ calcd. 650.1, found 650.1.

Preparation of **5** :

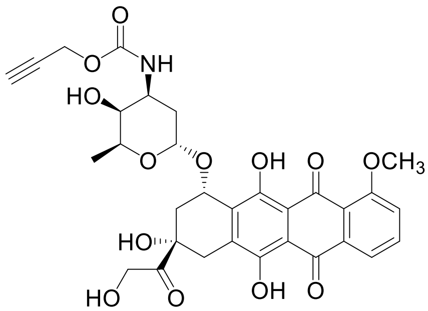

O-(4-Nitrophenyl)-O'-propargylcarbonate (13.7 mg, 0.062 mmol), which was prepared according to literature,[16] was added to Doxorubicin hydrochloride (39.5 mg, 0.068mmol) dissolved in DMF (1 ml). DIPEA (16 µl, 0.093 mmol) was then added and the mixture was stirred for 16 hr at room temperature. To workup, additional DMF (1 ml) was added. The reaction was then filtered and directly purified by reverse phase HPLC using method 8 (retention time ~19.3 min). Yield: 17.7 mg, 46%. Characterization matched a previous report of this known compound.[16] **^{1}H NMR** (Acetone-d6, 400 MHz) δ 8.02 (dd, *J* = 7.7, 0.9 Hz, 1H), 7.95-7.91 (m, 1H), 7.67 (d, *J* = 8.6 Hz, 1H), 5.48 (d, *J* = 3.8 Hz, 1H), 5.29-5.26 (m, 1H), 4.91 (s, 1H), 4.77-4.73 (m, 2H), 4.59 (d, *J* = 2.4 Hz, 2H), 4.30-4.24 (m, 1H), 4.07 (s, 3H), 4.05-4.01 (m, 1H), 3.92-3.83 (m, 1H), 3.69-3.63 (m, 2H), 3.22 (dd, *J* = 18.7, 1.7 Hz, 1H), 3.06 (d, *J* = 18.7 Hz, 1H), 2.53 (s, 1H), 2.51-2.47 (m, 1H), 2.27 (d, *J* = 4.6 Hz, 1H), 1.97 (s, 2H), 1.78-1.71 (m, 1H), 1.28 (d, *J* = 6.6 Hz, 3H). **LRMS** for $C_{31}H_{31}NO_{13}$ $[M+Na]^+$ calcd. 648.1, found 648.1.

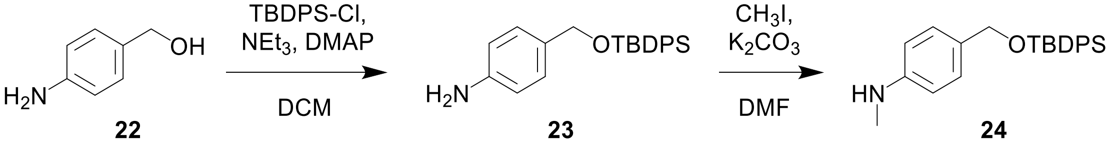

Scheme S11. Synthesis of **24**

Preparation of **23** :

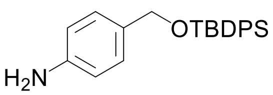

Compound **22** (200 mg, 1.62 mmol), NEt_3 (680 µl, 4.86 mmol), and DMAP (60 mg, 0.486 mmol) were dissolved in DCM (10 ml). TBDPS-Cl (520 µl, 2.00 mmol) was then added and the mixture was stirred for 16 hr at room temperature under N_2. To workup, the reaction was quenched with H_2O and the mixture was extracted with EtOAc (3×). The combined organic layers were dried over sodium sulfate and evaporated under vacuum. Flash column chromatography using a gradient of 10-20% EtOAc in Hex was used to purify the desired compound. Yield: 547 mg, 93%. R_f =0.19 (EtOAc/Hex, 1:5). Characterization matched a previous report of this known compound.[17] ^{1}**H NMR** ($CDCl_3$, 300 MHz) δ 7.72-7.65 (m, 4H), 7.45-7.34 (m, 6H), 7.12 (d, J = 8.3 Hz, 2H), 6.66 (d, J = 8.4 Hz, 2H), 4.66 (s, 2H), 3.61 (s, 2H), 1.07 (s, 9H).

Preparation of **24** :

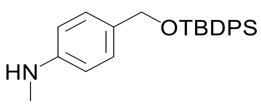

Compound **23** (668 mg, 1.85 mmol) and K_2CO_3 (383 mg, 2.77 mmol) were dissolved in DMF (5 ml) and stirred for 5 min. CH_3I (115 µl, 1.85 mmol) was then added in 3 portions of 40 µl that were separated by 15 minutes each time. The mixture was then stirred for 2 hr at 50°C under N_2. To workup, the reaction was diluted with EtOAc and then the organic layer was washed with H_2O, brine, dried over sodium sulfate, and evaporated under vacuum. Flash column chromatography using a gradient of 7-15% EtOAc in Hex was used to purify the desired compound. Yield: 260 mg, 38%. R_f =0.38 (EtOAc/Hex, 1:5). ^{1}**H NMR** ($CDCl_3$, 300 MHz) δ 7.73-7.67 (m, 4H), 7.45-7.34 (m, 6H), 7.16 (d, J = 8.4 Hz, 2H), 6.59 (d, J = 8.5 Hz, 2H), 4.66 (s, 2H), 2.84 (s, 3H), 1.07 (s, 9H); ^{13}**C NMR** ($CDCl_3$, 75 MHz): δ 148.7, 135.9, 134.1, 130.1, 129.8, 127.9, 127.8, 112.4, 65.7, 31.0, 26.9, 19.4. **HRMS** for $C_{24}H_{29}NOSi$ $[M+H]^+$ calcd. 376.2091, found 376.2100.

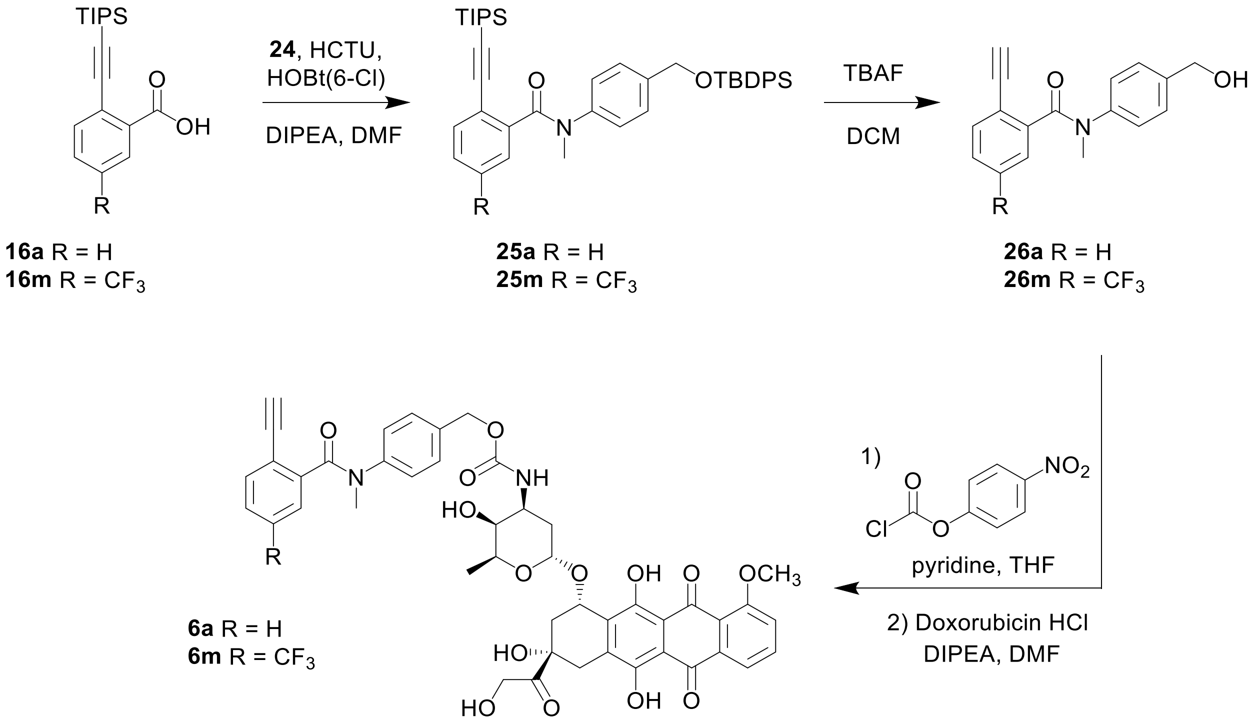

Scheme S12. Synthesis of **6a,m**

Preparation of **25a** :

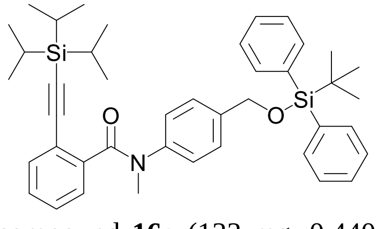

Compound **24** (165 mg, 0.440 mmol), compound **16a** (133 mg, 0.440 mmol), HCTU (363 mg, 0.879 mmol), HOBt(6-Cl) (149 mg, 0.879 mmol) were dissolved in DMF (7 ml). DIPEA (246 μl, 1.76 mmol) was then added and the mixture was stirred for 16 hr at room temperature under N_2. To workup, the reaction was diluted with EtOAc and then the organic layer was washed with H_2O, brine, dried over sodium sulfate, and evaporated under vacuum. Flash column chromatography using a gradient of 0-15% EtOAc in Hex was used to purify the desired compound. Yield: 163 mg, 56%. R_f =0.18 (EtOAc/Hex, 1:5). **^{1}H NMR** ($CDCl_3$, 400 MHz) δ 7.70-7.57 (m, 4H), 7.48-7.29 (m, 8H), 7.20-7.05 (m, 6H), 4.62 (s, 2H), 3.46 (s, 3H), 1.17 (s, 21H), 1.05 (s, 9H); **^{13}C NMR** ($CDCl_3$, 75 MHz): δ 140.0, 135.8, 133.5, 133.0, 129.9, 127.9, 127.0, 126.6, 95.1, 65.1, 37.4, 26.9, 19.3, 18.8, 11.4. **HRMS** for $C_{42}H_{53}NO_2Si_2$ $[M+H]^+$ calcd. 660.3688, found 660.3680.

Preparation of **25m** :

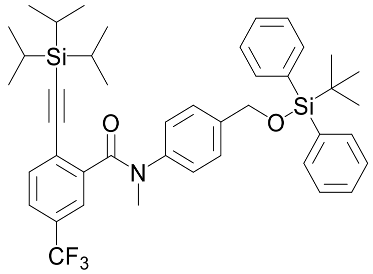

Compound **24** (180 mg, 0.481 mmol), compound **16m** (178 mg, 0.481 mmol), HCTU (397 mg, 0.962 mmol), HOBt(6-Cl) (163 mg, 0.962 mmol) were dissolved in DMF (5 ml). DIPEA (163 μl, 0.962 mmol) was then added and the mixture was stirred for 16 hr at room temperature under N_2. To workup, the reaction was diluted with

EtOAc and then the organic layer was washed with H_2O, brine, dried over sodium sulfate, and evaporated under vacuum. Flash column chromatography using a gradient of 0-10% EtOAc in Hex was used to purify the desired compound. Yield: 116 mg, 33%. R_f =0.31 (EtOAc/Hex, 1:5). **^{1}H NMR** ($CDCl_3$, 300 MHz) δ 7.74-7.69 (m, 1H), 7.66-7.57 (m, 3H), 7.44-7.30 (m, 10H), 7.16-7.11 (m, 3H), 4.63 (s, 2H), 3.47 (s, 3H), 1.18 (s, 21H), 1.04 (s, 9H); **^{13}C NMR** ($CDCl_3$, 75 MHz): δ 142.0, 141.3, 140.7, 135.9, 135.7, 135.0, 133.5, 133.4, 130.2, 130.0, 129.9, 127.9, 127.1, 126.8, 98.5, 65.0, 37.4, 26.8, 26.6, 19.3, 18.7, 17.7, 12.3, 11.4. **HRMS** for $C_{43}H_{52}F_3NO_2Si_2$ $[M+H]^+$ calcd. 728.3561, found 728.3555.

Preparation of **26a** :

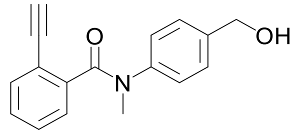

Compound **25a** (343 mg, 0.520 mmol) was dissolved in DCM (2 ml). TBAF (2.6 ml of 1.0M solution, 2.60 mmol) was then added and the mixture was stirred for 1 hr at room temperature under N_2. To workup, the reaction was diluted with EtOAc and then the organic layer was washed with H_2O, brine, dried over sodium sulfate, and evaporated under vacuum. Flash column chromatography using a gradient of 50-75% EtOAc in Hex was used to purify the desired compound. Yield: 129 mg, 93%. R_f =0.38 (EtOAc/Hex, 3:4). **^{1}H NMR** ($CDCl_3$, 400 MHz) δ 7.37-7.30 (m, 2H), 7.21-7.02 (m, 7H), 4.55 (s, 2H), 3.49 (s, 3H), 3.28 (s, 1H); **^{13}C NMR** ($CDCl_3$, 75 MHz): δ 169.9, 139.9, 133.1, 128.6, 128.5, 128.4, 127.5, 127.1, 126.5, 81.6, 81.2, 64.5, 37.4. **HRMS** for $C_{17}H_{15}NO_2$ $[M+H]^+$ calcd. 266.1176, found 266.1170.

Preparation of **26m** :

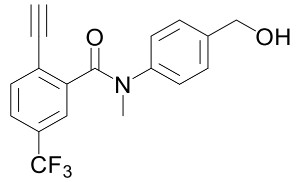

Compound **25m** (116 mg, 0.156 mmol) was dissolved in DCM (2 ml). TBAF (239 μl of 1.0M solution, 0.239 mmol) was then added and the mixture was stirred for 2 hr at 40°C. To workup, the reaction was diluted with EtOAc and then the organic layer was washed with H_2O, brine, dried over sodium sulfate, and evaporated under vacuum. Flash column chromatography using a gradient of 15-30% EtOAc in Hex was used to purify the desired compound. Yield: 27.9 mg, 52%. R_f =0.20 (EtOAc/Hex, 1:1). **^{1}H NMR** ($CDCl_3$, 300 MHz) δ 7.51-7.31 (m, 4H), 7.24-7.05 (m, 4H), 4.58 (s, 2H), 3.50 (s, 3H), 3.42 (s, 1H); **^{13}C NMR** ($CDCl_3$, 75 MHz): δ 168.4, 142.5, 141.3, 140.4, 133.6, 127.7, 127.3, 125.3, 124.7, 123.6, 121.7, 83.7, 80.4, 64.5, 37.5. **HRMS** for $C_{18}H_{14}F_3NO_2$ $[M+H]^+$ calcd. 334.1049, found 334.1046.

Preparation of **6a** :

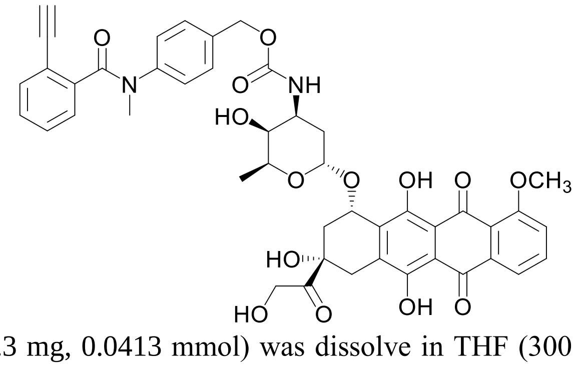

4-Nitrophenyl chloroformate (8.3 mg, 0.0413 mmol) was dissolve in THF (300 μl) and cooled to 0°C on an ice bath. Pyridine (4 μl, 0.0413 mmol) was added and after stirring for 20 min, compound **26a** (7.3 mg, 0.0275 mmol) dissolved in THF (200 μl) was added and the mixture was stirred for 16 hr at room temperature. To workup, THF was removed and the residue was redissolved in EtOAc. The organic layer was then washed with H_2O, brine, dried over sodium sulfate, and evaporated under vacuum. Flash column chromatography using a gradient of 25-

50% EtOAc in Hex was used to purify the desired intermediate. Yield: 5.2 mg, 44%. R_f =0.67 (EtOAc/Hex, 3:4). **HRMS** for $C_{24}H_{18}N_2O_6$ $[M+H]^+$ calcd. 431.1238, found 431.1234.

The intermediate compound and doxorubicin hydrochloride (7.0 mg, 0.012 mmol) were dissolved in DMF (300 µl). DIPEA (4 µl, 0.024 mmol) was then added and the mixture was stirred for 16 hr at room temperature. To workup, additional DMF (1 ml) was added. The reaction was then filtered and directly purified by reverse phase HPLC using method 8 (retention time ~28.1 min). Yield: 5.4 mg, 53%. **^{1}H NMR** (DMSO-d6, 400 MHz) δ 7.96-7.89 (m, 2H), 7.70-7.64 (m, 1H), 7.33-7.08 (m, 7H), 6.90 (d, *J* = 7.7 Hz, 1H), 5.46 (s, 1H), 5.27-5.19 (m, 1H), 4.99-4.93 (m, 1H), 4.90-4.77 (m, 3H), 4.70 (d, *J* = 5.3 Hz, 1H), 4.57 (d, *J* = 6.0 Hz, 2H), 4.33 (s, 1H), 4.15 (q, *J* = 6.8 Hz, 1H), 3.99 (s, 3H), 3.43 (s, 3H), 2.98 (d, *J* = 5.0 Hz, 2H), 2.23-2.08 (m, 2H), 1.88-1.76 (m, 1H), 1.49-1.40 (m, 1H), 1.12 (d, *J* = 6.5 Hz, 3H); **^{13}C NMR** (DMSO-d6, 75 MHz): δ 215.1, 187.9, 187.8, 169.2, 162.2, 157.2, 156.4, 156.1, 144.5, 142.1, 136.7, 136.5, 136.1, 133.4, 129.3, 129.1, 128.3, 127.7, 122.1, 120.6, 120.1, 119.5, 112.3, 112.2, 112.1, 99.8, 92.0, 82.7, 77.7, 76.9, 71.1, 70.0, 63.6, 63.1, 61.5, 56.7, 48.1, 40.7, 30.4, 27.8, 17.1. **HRMS** for $C_{45}H_{42}N_2O_{14}$ $[M+Na]^+$ calcd. 857.2528, found 857.2520.

Preparation of **6m** :

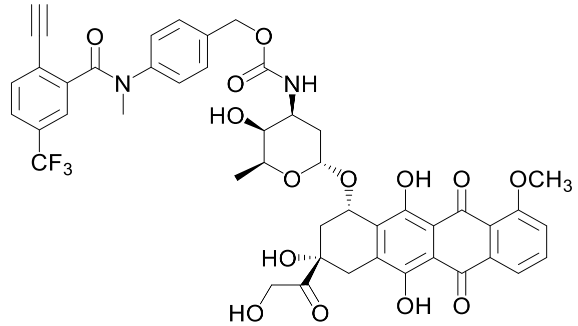

4-Nitrophenyl chloroformate (3.2 mg, 0.0158 mmol) was dissolve in THF (300 µl) and cooled to 0°C on an ice bath. Pyridine (2 µl, 0.0158 mmol) was added and after stirring for 20 min, compound **26m** (3.5 mg, 0.0105 mmol) dissolved in THF (200 µl) was added and the mixture was stirred for 16 hr at room temperature. To workup, THF was removed and the residue was redissolved in EtOAc. The organic layer was then washed with H_2O, brine, dried over sodium sulfate, and evaporated under vacuum. Flash column chromatography using a gradient of 25-50% EtOAc in Hex was used to purify the desired intermediate. Yield: 2.9 mg, 54%. R_f =0.63 (EtOAc/Hex, 1:1). **HRMS** for $C_{25}H_{17}F_3N_2O_6$ $[M+Na]^+$ calcd. 521.0931, found 521.0922.

The intermediate compound and doxorubicin hydrochloride (7.0 mg, 0.0114 mmol) were dissolved in DMF (300 µl). DIPEA (3 µl, 0.0171 mmol) was then added and the mixture was stirred for 16 hr at room temperature. To workup, additional DMF (1 ml) was added. The reaction was then filtered and directly purified by reverse phase HPLC using method 9 (retention time ~20.9 min). Yield: 3.9 mg, 75%. **^{1}H NMR** (DMSO-d6, 400 MHz) δ 7.94-7.83 (m, 2H), 7.73 (s, 1H), 7.62-7.48 (m, 3H), 7.45-7.35 (m, 1H), 7.24-7.07 (m, 3H), 6.88 (d, *J* = 7.8 Hz, 1H), 5.42 (s, 1H), 5.21 (s, 1H), 4.98-4.78 (m, 4H), 4.72-4.66 (m, 1H), 4.60-4.56 (m, 3H), 4.18-4.10 (m, 1H), 3.97 (s, 3H), 3.73-3.62 (m, 1H), 3.43 (s, 3H), 2.95 (d, *J* = 7.5 Hz, 2H), 2.22-2.09 (m, 2H), 1.87-1.77 (m, 1H), 1.49-1.38 (m, 1H), 1.12 (d, *J* = 6.4 Hz, 3H); **^{13}C NMR** (DMSO-d6, 75 MHz): δ 214.0, 186.7, 180.4, 166.9, 161.0, 156.3, 155.3, 142.3, 141.5, 136.3, 136.2, 135.7, 134.9, 133.3, 128.1, 127.1, 125.3, 125.2, 124.7, 124.6, 123.1, 120.2, 120.1, 119.8, 119.1, 110.9, 110.7, 110.5, 100.4, 87.0, 80.2, 75.0, 70.0, 68.0, 66.7, 64.3, 63.7, 56.6, 47.2, 36.7, 32.1, 29.8, 17.0. **HRMS** for $C_{46}H_{41}F_3N_2O_{14}$ $[M+Na]^+$ calcd. 925.2402, found 925.2412.

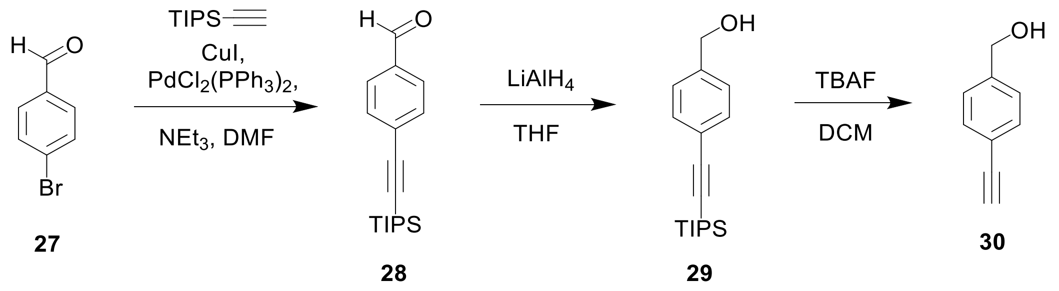

Scheme S13. Synthesis of **30**

Preparation of **28** :

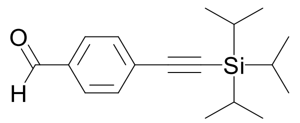

Compound **27** (500 mg, 2.702 mmol), CuI (103 mg, 0.540 mmol), and $PdCl_2(PPh_3)_2$ (189 mg, 0.270 mmol) were dissolved in DMF (2 ml) and NEt_3 (2 ml). The mixture was degassed with N_2 for 5 min. (Triisopropylsilyl)acetylene (1.2 ml, 5.404 mmol) was then added and the mixture was stirred for 16 hr at 40°C under N_2. To workup, the reaction was diluted with EtOAc and then the organic layer was washed with H_2O, brine, dried over sodium sulfate, and evaporated under vacuum. Flash column chromatography using a gradient of 0-10% EtOAc in Hex was used to purify the desired compound. Yield: 770 mg, quan. R_f =0.52 (EtOAc/Hex, 1:10). Characterization matched a previous report of this known compound.[18] **^{1}H NMR** (CD_2Cl_2, 300 MHz) δ 9.99 (s, 1H), 7.83 (d, *J* = 8.2 Hz, 2H), 7.63 (d, *J* = 8.1 Hz, 2H), 1.15 (s, 21H).

Preparation of **29** :

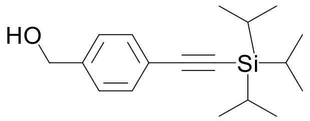

LiAlH4 (205 mg, 5.404 mmol) was suspended in THF (10 ml) and cooled to O°C on an ice bath. Compound **28** (770 mg, 2.702 mmol) dissolved in THF (20 ml) was then added dropwise and the mixture was stirred for 2 hr at room temperature under N_2. To workup, the mixture was cooled on an ice bath, followed by the addition of H_2O dropwise to quench the reaction. When bubbles stopped forming, HCl (2 ml of a 1N solution) was added, followed by the addition of EtOAc. The organic layer was washed with H_2O, brine, dried over sodium sulfate, and evaporated under vacuum. Flash column chromatography using a gradient of 10-25% EtOAc in Hex was used to purify the desired compound. Yield: 463 mg, 60%. R_f =0.31 (EtOAc/Hex, 1:4). Characterization matched a previous report of this known compound.[19] **^{1}H NMR** ($CDCl_3$, 300 MHz) δ 7.47 (d, *J* = 8.1 Hz, 2H), 7.29 (d, *J* = 8.4 Hz, 2H), 4.66 (d, *J* = 5.3 Hz, 2H), 1.13 (s, 21H).

Preparation of **30** :

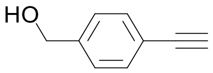

Compound **29** (463.3 mg, 1.609 mmol) was dissolved in DCM (1 ml). TBAF (2 ml of 1.0M solution, 2.413 mmol) was then added and the mixture was stirred for 2 hr at room temperature. To workup, the reaction was diluted with EtOAc and then the organic layer was washed with H_2O, brine, dried over sodium sulfate, and evaporated under vacuum. Flash column chromatography using a gradient of 20-40% EtOAc in Hex was used to purify the desired compound. Yield: 212 mg, quan. R_f =0.24 (EtOAc/Hex, 1:4). Characterization matched a previous report of this known compound.[20] **^{1}H NMR** ($CDCl_3$, 300 MHz) δ 7.47 (d, *J* = 8.1 Hz, 2H), 7.29 (d, *J* = 8.4 Hz, 2H), 4.63 (s, 2H), 3.07 (s, 1H).

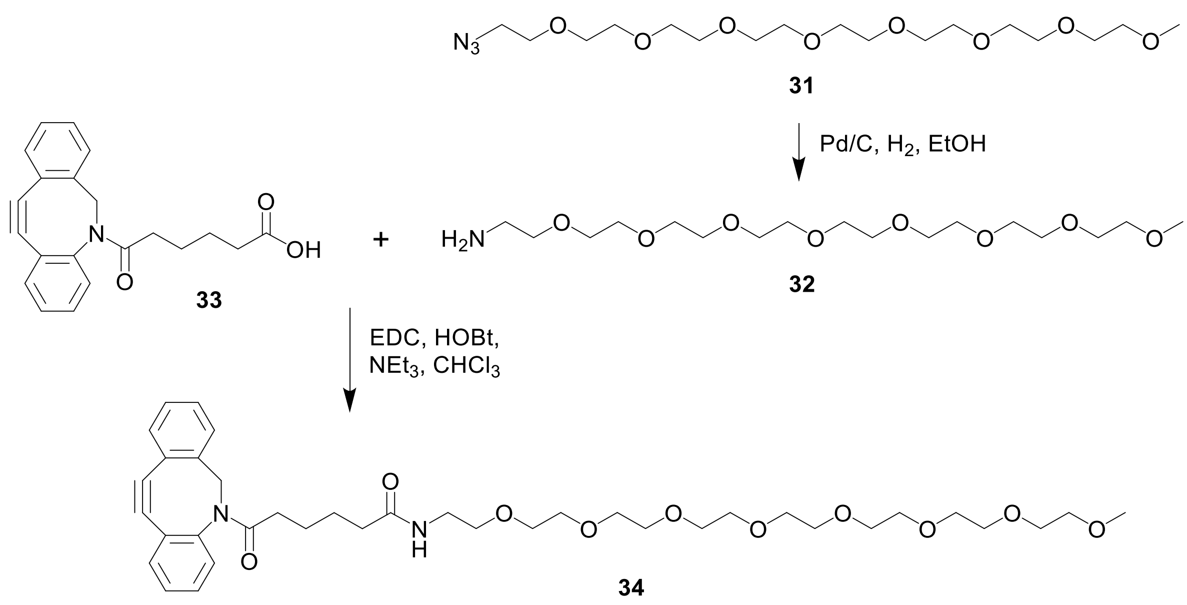

Scheme S14. Synthesis of **34**

Preparation of **32** :

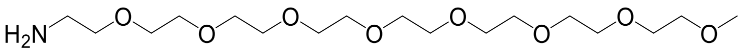

Compound **31** (7.5 mg, 0.0183 mmol), and 10% Pd/C (1 mg, 0.006 mmol) were dissolved in EtOH (5 ml). The reaction mixture was first purged with N_2 gas, followed by H_2 gas. The mixture was then stirred for 16 hr at room temperature under H_2. To workup, the reaction was diluted with EtOAc and then filtered through celite. The collected organic layer was dried over sodium sulfate, and evaporated under vacuum to give a clear oil. Yield: 7.0 mg, quan. Characterization matched a previous report of this known compound.[21] **HRMS** for $C_{17}H_{37}NO_8$ $[M+H]^+$ calcd. 384.2592, found 284.2603.

Preparation of **34** :

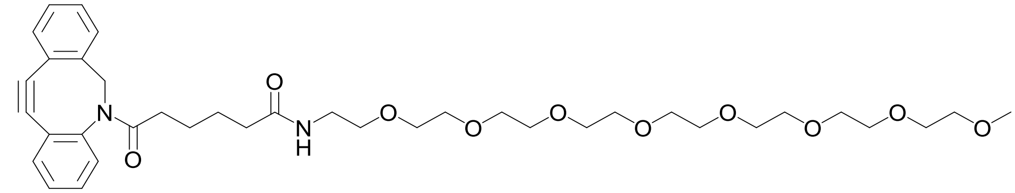

Compound **32** (8.5 mg, 0.022 mmol), compound **33** (7 mg, 0.022 mmol), EDC (7 mg, 0.044 mmol), HOBt (6 mg, 0.022 mmol) were dissolved in $CHCl_3$ (0.5 ml). NEt_3 (6 μl, 0.044 mmol) was then added and the mixture was stirred for 16 hr at room temperature under N_2. To workup, the solvent was removed by vacuum and the residue was redissolved in acetonitrile with 1-2 drops of H_2O. The mixture was then filtered and directly purified by reverse phase HPLC using method 10 (retention time ~27.7 min). Yield: 3.9 mg, 25%. **^{1}H NMR** (DMSO-d6, 400 MHz) δ 7.72-7.29 (m, 8H), 5.04 (d, *J* = 14.0 Hz, 1H), 3.61 (d, *J* = 14.0 Hz, 1H), 3.58-3.41 (m, 32H), 3.23 (s, 3H), 2.22-2.13 (m, 1H), 1.89-1.81 (m, 2H), 1.79-1.69 (m, 1H), 1.37-1.10 (m, 4H); **^{13}C NMR** (DMSO-d6, 75 MHz): δ 172.0, 171.8, 152.0, 148.6, 132.5, 129.6, 129.0, 128.2, 128.1, 127.8, 126.9, 125.3, 122.6, 121.5, 114.5, 108.3, 71.3, 69.8 (br s), 69.7, 69.6, 69.1, 58.1, 54.8, 34.9, 33.9, 24.4. **HRMS** for $C_{38}H_{54}N_2O_{10}$ $[M+H]^+$ calcd. 699.3851, found 699.3857.

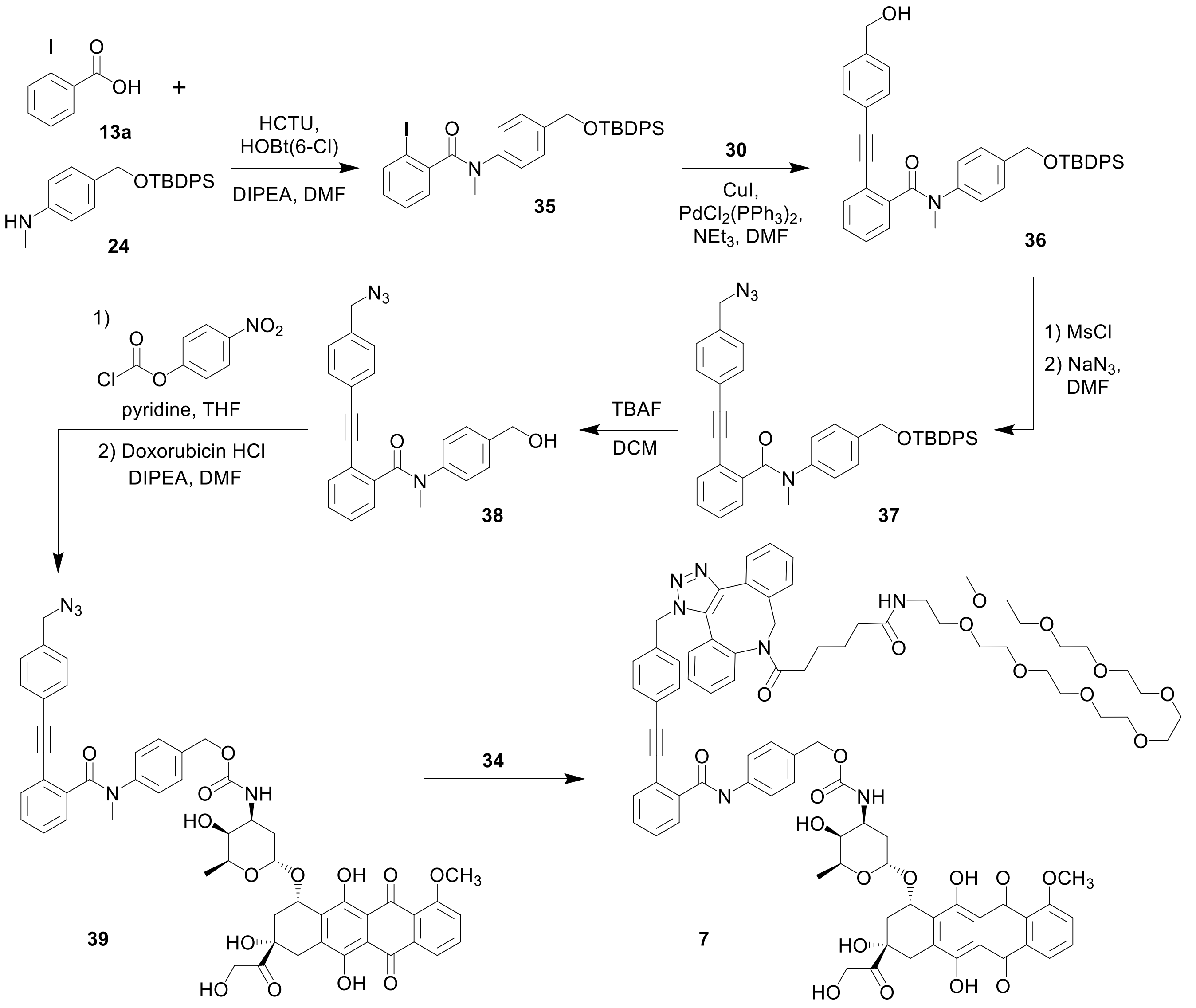

Scheme S15. Synthesis of **7**

Preparation of **35** :

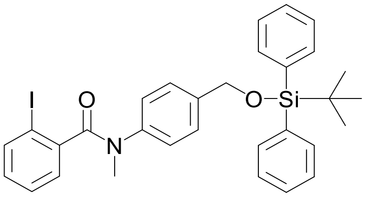

Compound **13a** (306 mg, 1.24 mmol), **24** (309 mg, 0.82 mmol), HCTU (680 mg, 1.65 mmol), HOBt(6-Cl) (279 mg, 1.65 mmol) were dissolved in DMF (5 ml). DIPEA (279 µl, 1.65 mmol) was then added and the mixture was stirred for 16 hr at 50°C under N_2. To workup, the reaction was diluted with EtOAc and then the organic layer was washed with H_2O, brine, dried over sodium sulfate, and evaporated under vacuum. Flash column chromatography using a gradient of 15-25% EtOAc in Hex was used to purify the desired compound. Yield: 112 mg, 15%. R_f=0.17 (EtOAc/Hex, 1:4). **^{1}H NMR** ($CDCl_3$, 400 MHz) δ 7.73-7.60 (m, 6H), 7.46-7.32 (m, 9H), 7.12-7.02 (m, 2H), 6.89-6.83 (m, 1H), 4.64 (s, 2H), 3.51 (s, 3H), 1.05 (s, 9H); **^{13}C NMR** ($CDCl_3$, 75 MHz): δ 170.5, 142.8, 142.2, 140.3, 139.4, 135.7, 133.5, 130.0, 129.9, 128.8, 127.9, 127.5, 127.0, 126.7, 125.8, 93.8, 65.0, 37.5, 26.9, 19.3. **HRMS** for $C_{31}H_{32}INO_2Si$ $[M+H]^+$ calcd. 606.1320, found 606.1345.

Preparation of **36** :

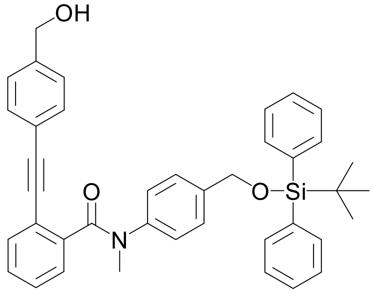

Compound **35** (257 mg, 0.425 mmol), CuI (16 mg, 0.085 mmol), and $PdCl_2(PPh_3)_2$ (30 mg, 0.043 mmol) were dissolved in DMF (2 ml) and NEt_3 (2 ml). The mixture was degassed with N_2 for 5 min. Compound **30** (87 mg, 0.552 mmol) dissolved in DMF (0.5 ml) was then added and the mixture was stirred for 16 hr at 40°C under N_2. To workup, the reaction was diluted with EtOAc and then the organic layer was washed with H_2O, brine, dried over sodium sulfate, and evaporated under vacuum. Flash column chromatography using a gradient of 30-50% EtOAc in Hex was used to purify the desired compound. Yield: 135 mg, 52%. R_f =0.40 (EtOAc/Hex, 1:1). **^{1}H NMR** ($CDCl_3$, 300 MHz) δ 7.71-7.29 (m, 18H), 7.22-6.98 (m, 4H), 4.71 (s, 2H), 4.63 (s, 2H), 3.52 (s, 3H), 1.05 (s, 9H); **^{13}C NMR** ($CDCl_3$, 75 MHz): δ 170.3, 141.7, 139.9, 135.8, 133.6, 132.4, 132.3, 132.1, 130.0, 128.8, 128.7, 127.9, 127.1, 126.9 (br s), 126.5 (br s), 92.2, 88.6, 65.1, 65.0, 54.4, 26.9, 19.3. **HRMS** for $C_{40}H_{39}NO_3Si$ $[M+H]^+$ calcd. 610.2772, found 610.2785.

Preparation of **37** :

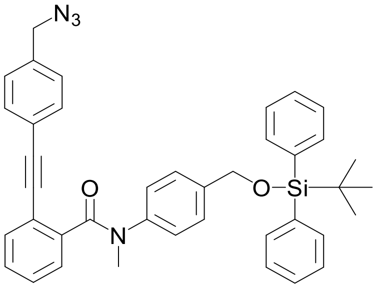

Compound **36** (135 mg, 0.221 mmol) was dissolved in THF (5 ml) and cooled to 0°C on an ice bath. Mesyl chloride (22 µl, 0.287 mmol) and triethylamine (40 µl, 0.287 mmol) was then added and the mixture was stirred for 1 hr at 0°C under N_2. To workup, the reaction was quenched with cold Na_2CO_3 and then the product was extracted with DCM. The combined organic layers were dried over sodium sulfate, and evaporated under vacuum to give the intermediate compound as an oil.

In the next step, the intermediate compound was dissolved in DMF (5 ml). Sodium azide (29 mg, 0.442 mmol) was then added and the mixture was stirred for 16 hr at room temperature under N_2. To workup, the reaction was diluted with EtOAc and then the organic layer was washed with H_2O, brine, dried over sodium sulfate, and evaporated under vacuum. Flash column chromatography using a gradient of 10-25% EtOAc in Hex was used to purify the desired compound. Yield: 140 mg, 99%. R_f =0.71 (EtOAc/Hex, 1:1). **^{1}H NMR** ($CDCl_3$, 300 MHz) δ 7.51-7.75 (m, 7H), 7.28-7.44 (m, 11H), 7.02-7.20 (m, 4H), 4.64 (s, 2H), 4.38 (s, 2H), 3.53 (s, 3H), 1.05 (s, 9H); **^{13}C NMR** ($CDCl_3$, 75 MHz): δ 171.2, 139.9, 136.0, 135.0, 133.6, 132.3, 132.2, 130.0, 128.6, 128.4, 127.9, 126.9 (br s), 126.6 (br s), 92.7, 88.8, 65.1, 60.5, 54.6, 26.9, 19.3. **HRMS** for $C_{40}H_{38}N_4O_2Si$ $[M+H]^+$ calcd. 635.2837, found 635.2840.

Preparation of **38** :

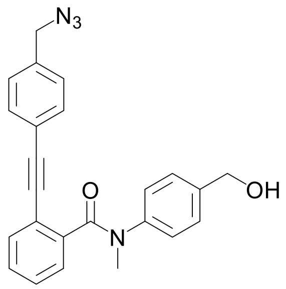

Compound **37** (57.3 mg, 0.090 mmol) was dissolved in DCM (1 ml). TBAF (135 μl of 1.0M solution, 0.135 mmol) was then added and the mixture was stirred for 2 hr at 40°C. To workup, the reaction was diluted with EtOAc and then the organic layer was washed with H_2O, brine, dried over sodium sulfate, and evaporated under vacuum. Flash column chromatography using a gradient of 25-50% EtOAc in Hex was used to purify the desired compound. Yield: 29.9 mg, 84%. R_f =0.23 (EtOAc/Hex, 1:1). **^{1}H NMR** ($CDCl_3$, 300 MHz) δ 7.69-7.47 (m, 1H), 7.56 (d, *J* = 7.9 Hz, 2H), 7.45-7.28 (m, 1H), 7.33 (d, *J* = 8.0 Hz, 2H), 7.24-7.00 (m, 6H), 4.57 (s, 2H), 4.38 (s, 2H), 3.51 (s, 3H); **^{13}C NMR** ($CDCl_3$, 75 MHz): δ 170.2, 139.7, 136.1, 132.4, 132.3, 132.2, 128.5, 128.8, 128.7, 128.5, 128.2, 127.4 (br s), 127.1 (br s), 92.7, 88.2, 64.6, 60.5, 54.6. **HRMS** for $C_{24}H_{20}N_4O_2$ $[M+H]^+$ calcd. 397.1659, found 397.1663.

Preparation of **39** :

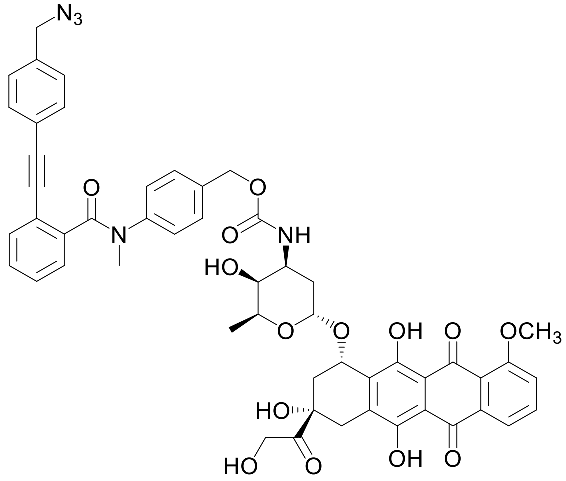

4-Nitrophenyl chloroformate (9.0 mg, 0.046 mmol) was dissolve in THF (300 μl) and cooled to 0°C on an ice bath. Pyridine (4 μl, 0.046 mmol) was added and after stirring for 20 min, compound **38** (12.1 mg, 0.031 mmol) dissolved in THF (200 μl) was added and the mixture was stirred for 16 hr at room temperature. To workup, THF was removed and the residue was redissolved in EtOAc. The organic layer was then washed with H_2O, brine, dried over sodium sulfate, and evaporated under vacuum. Flash column chromatography using a gradient of 25-50% EtOAc in Hex was used to purify the desired intermediate. Yield: 11.7 mg, 68%. R_f =0.36 (EtOAc/Hex, 1:1). **HRMS** for $C_{31}H_{23}N_5O_6$ $[M+H]^+$ calcd. 562.1721, found 562.1728.
The intermediate compound and doxorubicin hydrochloride (24.0 mg, 0.042 mmol) were dissolved in DMF (300 μl). DIPEA (7 μl, 0.042 mmol) was then added and the mixture was stirred for 16 hr at room temperature. To workup, additional DMF (1 ml) was added. The reaction was then filtered and directly purified by reverse phase HPLC using method 11 (retention time ~20.4 min). Yield: 10.4 mg, 51%. **^{1}H NMR** (DMF-d7, 400 MHz) δ 8.00-7.92 (m, 2H), 7.73 (dd, *J* = 7.7, 1.9 Hz, 1H), 7.68-7.49 (m, 4H), 7.47-7.14 (m, 7H), 6.78 (d, *J* = 8.1 Hz, 1H), 5.57 (s, 1H), 5.40-5.36 (m, 1H), 5.15-5.09 (m, 1H), 5.02-4.82 (m, 3H), 4.80-4.71 (m, 3H), 4.59 (s, 2H), 4.09 (s, 3H), 3.49 (s, 3H), 3.10 (q, *J* = 18.5 Hz, 2H), 2.45-2.37 (m, 1H), 2.26 (dd, *J* = 14.5, 5.3 Hz, 1H), 2.03 (td, *J* = 13.0, 3.8 Hz, 1H), 1.70-1.61 (m, 1H), 1.23 (d, *J* = 6.5 Hz, 3H); **^{13}C NMR** (THF-d8, 75 MHz): δ 214.7, 187.8, 187.5, 169.2, 162.3, 157.3, 156.4, 156.0, 144.3, 141.8, 137.4, 136.6, 136.3, 135.8, 134.7, 132.6, 132.4, 129.0, 128.9, 128.8, 128.6, 128.4, 127.6, 127.5, 127.4, 119.8, 119.7, 112.1, 102.1, 92.9, 76.8, 70.7, 69.7, 65.7, 65.5, 56.6, 54.7, 48.0, 36.8, 33.9, 30.8, 17.0. **HRMS** for $C_{52}H_{47}N_5O_{14}$ $[M+Na]^+$ calcd. 988.3012, found 988.3022.

Preparation of **7** :

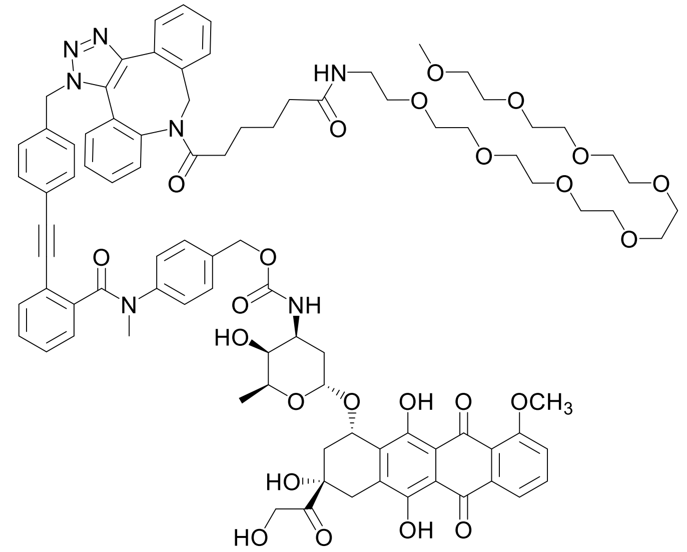

Compound **39** (5.3 mg, 0.0055 mmol) and **34** (3.9 mg, 0.0055 mmol) were dissolved in a mixture of 1:1:1 PBS/MeOH/ACN (1.2 ml). The mixture was stirred for 16 hr at room temperature. To workup, additional MeOH (1 ml) was added. The reaction was then filtered and directly purified by reverse phase HPLC using method 12 (retention time 17.7 min). Yield: 4.7 mg, 51%. **^{1}H NMR** (1,4-Dioxane-d8, 400 MHz) δ 8.04 (d, *J* = 7.7 Hz, 1H), 7.82 (t, *J* = 8.0 Hz, 1H), 7.61-7.43 (m, 7H), 7.40-7.21 (m, 9H), 7.20-7.03 (m, 6H), 6.93 (s, 1H), 6.84 (s, 1H), 6.22-5.98 (m, 1H), 5.90-5.66 (m, 1H), 5.41-5.35 (m, 1H), 5.25-5.19 (m, 1H), 4.97-4.80 (m, 2H), 4.78-4.75 (m, 1H), 4.72 (t, *J* = 5.8 Hz, 1H), 4.30-4.22 (m, 1H), 4.03 (s, 3H), 3.85-3.73 (m, 4H), 3.56-3.40 (m, 35H), 3.30 (s, 3H), 3.21-3.06 (m, 2H), 2.46-2.35 (m, 1H), 2.20-2.08 (m, 1H), 2.03-1.87 (m, 2H), 1.84-1.73 (m, 1H), 1.72-1.52 (m, 2H), 1.43-1.27 (m, 4H), 1.23 (d, *J* = 6.5 Hz, 3H); **^{13}C NMR** (1,4-Dioxane-d8, 75 MHz): δ 214.9, 187.8, 187.7, 172.8, 172.4, 162.1, 157.1, 156.3, 156.0, 141.9, 140.1, 139.3, 137.8, 137.6, 137.2, 136.5, 136.2, 136.0, 135.9, 135.6, 135.5, 135.4, 135.1, 134.8, 133.3, 132.9, 132.8, 132.1, 131.2, 130.5, 130.3, 130.0, 129.8, 129.4, 129.1, 129.0, 128.9, 128.8, 128.0, 127.5, 125.5, 122.0, 120.8, 119.9, 119.3, 112.2, 112.1, 110.0, 101.8, 76.7, 72.5, 71.1, 70.7, 58.7, 56.6, 47.9, 41.0, 39.6, 36.1, 33.9, 30.8, 25.4, 24.8, 16.9. **HRMS** for $C_{90}H_{101}N_7O_{24}$ $[M+Na]^+$ calcd. 1686.6796, found 1686.6798.

1.5.4 Gold Catalyst Complexes

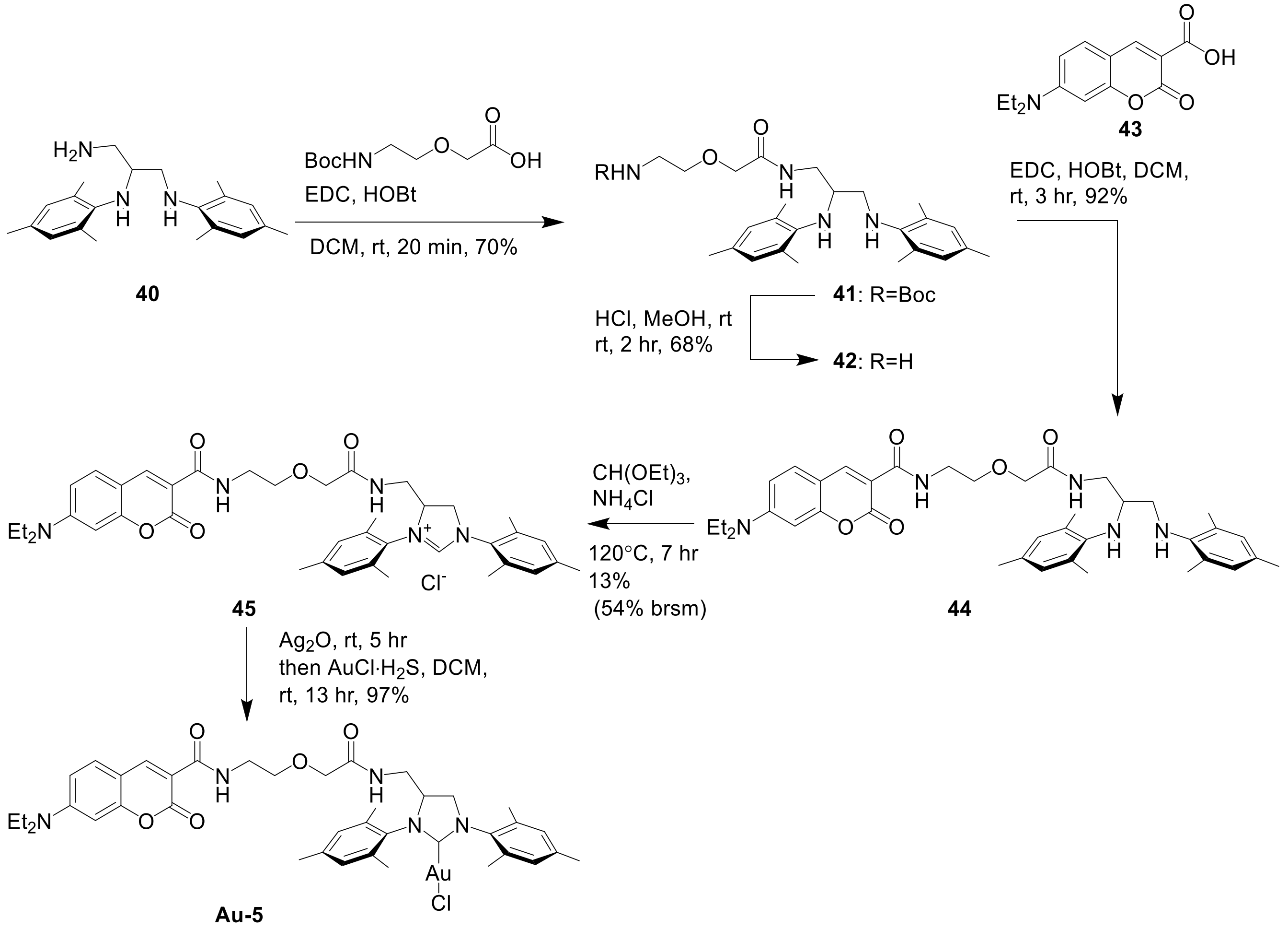

Scheme S16. Synthesis of **Au-5**

Preparation of **41** :

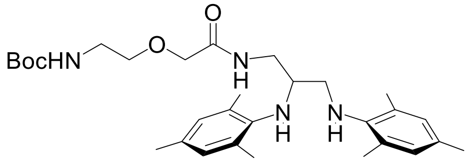

Compound **40** was prepared according to the previous literature.[22] Compound **40** (275.0 mg, 844.9 μmol) and Boc-amino-3-oxapentanoic acid (277.8 mg, 1.270 mmol) were dissolved in DCM. EDC (1.270 mmol) and HOBt (1.270 mmol) were added and the mixture was stirred at rt for 20 min. To workup, H_2O was added to the mixture and the product was extracted to CH_2Cl_2.. The organic layer was dried over Na_2SO_4 and then evaporated under vacuum after the filteration. Flash column chromatography using a gradient of 25-50% of EtOAc in Hex was used to purify the desired compound. Yield: 311.5 mg, 70%. R_f=0.74 ($CHCl_3$/MeOH, 9:1). ^{1}**H NMR** ($CDCl_3$, 400 MHz) δ 7.08 (m, 1H), 6.81 (s, 2H), 6.79 (s,2H), 3.94 (s, 2H), 3.72-3.60 (m, 2H), 3.60-3.50 (m, 2H), 3.46-3.21 (m, 4H), 3.09 (dd, *J* =12.4, 6.0 Hz, 1H), 2.90 (dd, *J* = 12.4, 5.2 Hz, 1H), 2.25 (s, 6H), 2.22 (s, 3H), 2.20 (s, 3H), 2.19 (s, 6H), 1.43 (s, 9H) ^{13}**C NMR** ($CDCl_3$, 100 MHz): δ 170.0, 156.0, 143.3, 141.3, 131.7, 131.4, 130.2, 130.0, 129.1, 79.8, 71.0, 70.5, 56.9, 51.2, 42.1, 40.5, 28.5, 20.6, 19.1, 18.5. **HRMS** for $C_{30}H_{46}N_4O_4$ $[M+H]^+$ calcd. 527.3592, found 527.3597.

Preparation of **42** :

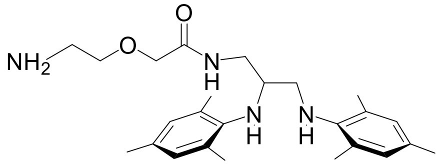

Compound **41** (314.9 mg, 591.4 μmol) was dissolved in 4.6 M HCl solution in MeOH (8.3 mL). The reaction solution was stirred at rt for 2 h. To workup, the reaction solution was neutralized with 1.5 g of NaOH and sat. aq. $NaHCO_3$. The product was extracted to CH_2Cl_2. The organic layer was dried over Na_2SO_4, filtered, and then evaporated to obtain the desired compound. Yield: 172.5 mg, 68%. R_f=0.23 ($CHCl_3$/MeOH, 4:1). **^{1}H NMR** ($CDCl_3$, 400 MHz) δ 7.52 (m, 1H), 6.81 (s, 2H), 6.78 (s, 2H), 3.98 (s, 2H), 3.65-3.47 (m, 4H), 3.42 (dt, *J*=13.6, 5.6 Hz, 1H), 3.07 (dd, *J*=12.4, 6.0 Hz, 1H), 2.88 (dd, *J*=12.4, 5.2 Hz, 1H, 2.83 (dd, *J*=5.6, 4.4 Hz, 2H), 2.24 (s, 6H), 2.21 (s, 3H), 2.19 (s, 3H), 2.17 (s, 6H). **^{13}C NMR** ($CDCl_3$, 100 MHz): δ170.6, 143.5, 141.3, 131.5, 131.3, 130.0, 129.6, 129.0, 74.0, 70.5, 57.0, 51.0, 41.7, 41.5, 20.6, 19.2, 18.5. **HRMS** for $C_{25}H_{38}N_4O_2$ $[M+H]^+$ calcd.427.3068, found. 427.3071.

Preparation of **44** :

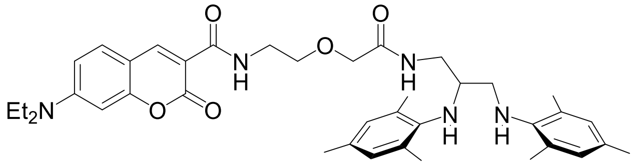

Compound **42** (160.7 mg, 376.7 μmol) and **43** (108.3 mg, 414.4 μmol) were dissolved in DCM (15 mL). EDC (79.4 mg, 414.4 μmol) and HOBt (55.6 mg, 414.4 μmol) were added and the mixture was stirred at rt for 2 h. Then, EDC (79.4 mg, 414.4 μmol) and HOBt (55.6 mg, 414.4 μmol) were added and the mixture was stirred further for 1 hr. To workup, H_2O was added to the mixture and the product was extracted to CH_2Cl_2.. The organic layer was dried over Na_2SO_4 and then evaporated under vacuum after the filteration. Flash column chromatography using a gradient of 0-5% of MeOH in $CHCl_3$ was used to purify the desired compound. Yield: 231.2 mg, 92%. R_f=0.53 ($CHCl_3$/MeOH, 9:1). **^{1}H NMR** ($CDCl_3$, 400 MHz) δ 9.07 (m, 1H), 8.60 (s, 1H), 7.31 (d, *J*=9.2 Hz, 1H), 6.724 (s, 2H), 6.716 (s, 2H), 6.61 (dd, *J*=9.2, 2.4 Hz, 1H), 6.43 (d, *J* =2.4 Hz, 1H), 4.02 (s, 2H), 3.69-3.49 (m, 7H), 3.45 (q, *J*=7.2 Hz, 4H), 3.05 (dd, *J*=12.8, 5.6 Hz, 1H), 2.86 (dd, *J*=12.8, 4.4 Hz, 1H), 2.21 (s, 6H), 2.16 (s, 6H), 2.15 (s, 6H), 1.25 (t, *J*=7.2 Hz, 6H). **^{13}C NMR** ($CDCl_3$, 100 MHz) δ 170.3, 163.5, 163.0, 157.7, 148.3, 143,7, 141.5, 133.5, 131.3, 131.2, 130.9, 130.0, 129.8, 129.5, 129.2, 110.1, 110.0, 108.5, 96.6,70.6, 70.6, 57.0, 50.7, 45.2, 41.7, 39.3, 20.6, 19.1, 18.5, 12.6. **HRMS** for $C_{39}H_{52}N_5O_5$ $[M+H]^+$ calcd.670.3963, found. 670.3977.

Preparation of **45** :

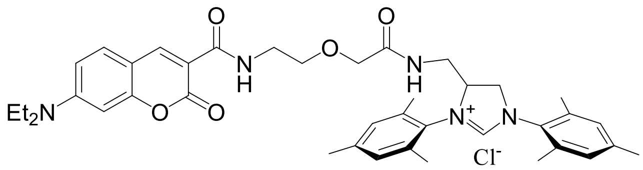

Compound **44** (165.6 mg, 247.2 μmol) was dissolved in $CH(OEt)_3$ (3.0 mL). NH_4Cl (13.2 mg, 247.2 μmol) was added and the mixture was stirred at 120 °C for 7 hrs. After cooled to rt, the mixture was filtered and the filterate was concentrated under vacuum. The product was sedimentated in Et_2O, filtered with Celite, and recovered with $CHCl_3$. The resulting solution was evaporated under vacuum. Flash column chromatography using a gradient of 5-15% of MeOH in $CHCl_3$ was used to purify the desired compound. From the extract in Et_2O, the 76% yield of the starting material **44** was purified with flash column chromatography using a gradient of 0-7.5% of MeOH in $CHCl_3$. Yield: 23.6 mg, 13% (54% brsm), R_f= 0.56 ($CHCl_3$/MeOH, 8:2). **^{1}H NMR** ($CDCl_3$, 400 MHz) 9.55 (s, 1H), 8.99 (m ,1H), 8.63 (s, 1H), 8.01 (t, *J*=6.0 Hz, 1H), 7.42 (d, *J*=9.2 Hz, 1H), 6.96 (s, 2H), 6.92 (s, 2H), 6.67 (dd, *J*=8.8, 2.4 Hz, 1H), 6.44 (d, *J*= 2.4 Hz, 1H), 5.32 (m, 1H), 4.58 (t, *J*=12.0 Hz, 1H), 4.48 (dd, *J* =12.0, 8.0 Hz, 1H), 4.03-3.86 (m, 3H), 3.76-3.59 (m, 5H), 3.53-3.42 (m, 5H), 2.47 (s+brs, 3H+3H), 2.44 (s, 3H), 2.36 (brs, 3H), 2.27 (s, 3H), 2.25 (s, 3H), 1.27 (t, *J*=7.2 Hz, 6H). **^{13}C NMR** ($CDCl_3$, 100 MHz) 171.0, 163.4, 163.1, 160.4, 157.8, 152.9, 148.4, 140.8, 140.6, 135.2, 131.4, 130.7, 130.5, 130.2, 129.0, 124.5, 121.9, 110.3, 110.0, 108.5, 96.5, 70.7, 70.4, 62.5, 55.6, 45.3, 40.1, 39.3, 29.9, 21.2, 19.0, 18.7, 12.6. **HRMS** for $C_{40}H_{50}N_5O_5Cl$ $[M-Cl]^+$ calcd. 680.3806, found. 680.3846.

Preparation of **Au-5** :

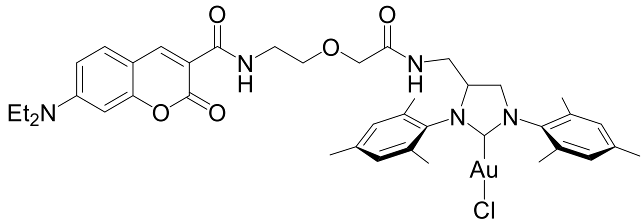

Compound **45** (23.6 mg, 32.7 μmol) was dissolved in DCM (3.0 mL). Ag_2O (3.8 mg, 16.4 μmol) was added and the mixture was stirred at rt for 4 hrs. The mixture was filtered with Celite to remove the precipitated salt. To the filtrate was added chloro(dimethylsulfide)gold (I) (8.7 mg, 32.7 μmol) and stirred at rt for 13 hrs. The mixture was filtered with Celite and the filtrate was evaporated under vacuum to obtain **Au-5** as a brown powder. Yield: 28.9 mg, 97%. **^{1}H NMR** ($CDCl_3$, 400 MHz) 9.03 (m, 1H), 8.64 (s, 1H), 7.40 (d, *J*=9.6 Hz, 1H), 7.04 (dd, *J*=7.2, 4.8 Hz, 1H), 6.93 (s, 1H), 6.90 (s, 3H), 6.69 (dd, *J*=8.8, 2.4 Hz, 1H), 6.47 (d, *J*=2.4 Hz, 1H), 4.67 (m, 1H), 4.13 (t, *J*=9.6 Hz, 1H), 3.98-3.84 (m, 4H), 3.67-3.47 (m, 8H), 3.33 (dt, *J*=13.2, 4.8 Hz, 1H), 2.41, (s, 3H) 2.38 (s, 3H), 2.33 (s, 3H), 2.29 (s, 3H), 2.27 (s, 6H), 1.29 (t, *J*=7.2 Hz, 1H). **^{13}C NMR** ($CDCl_3$, 100 MHz) 195.7, 170.3, 163.4, 163.2, 157.8, 153.0, 148.4, 139.0, 138.9, 136.5, 135.9, 135.4, 135.3, 134.6, 133.6, 131.4, 130.3, 130.2, 129.9, 129.8, 110.4, 109.9, 108.4, 99.4, 70.5, 70.4, 62.6, 55.2, 45.3, 41.3, 39.1, 29.8, 21.2, 19.3, 18.2, 18.1, 18.0, 12.6. **HRMS** for $C_{40}H_{50}N_5O_5AuCl$ $[M-Cl]^+$ calcd. 877.3394, found. 876.3455.

1.5.5 Reaction by-products

Preparation of **46** :

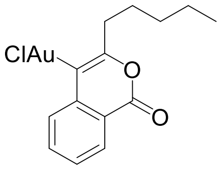

Compound **1q** (50 mg, 0.133 mmol) and **Au-1** (42 mg, 0.106 mmol) were dissolved in THF (2 ml) and PBS buffer pH 7.4 (2 ml). The mixture was stirred for 16 hr at 37 °C. To workup, the reaction was diluted with diethyl ether and then the organic layer was extracted, dried over magnesium sulfate, and evaporated under vacuum. Flash column chromatography using a gradient of 10-25% EtOAc in Hex was used to purify the desired compound. Yield: 19 mg, 40% based on Au. R_f =0.30 (EtOAc/Hex, 1:4). **^{1}H NMR** ($CDCl_3$, 300 MHz) δ 7.22-6.99 (m, 4H), 1.81 (t, *J*= 7.4, 2H), 1.41-1.17 (m, 6H), 0.87 (t, *J*= 6.8, 3H); **^{13}C NMR** ($CDCl_3$, 75 MHz): δ 157.0, 156.1, 135.9, 131.2, 128.6, 128.4, 127.6, 120.0, 97.5, 33.0, 31.3, 25.9, 22.4, 14.0. **LRMS** for $C_{14}H_{15}O_2AuCl$ $[M-Cl]^+$ calcd. 412.2, found. 412.2.

2. Cell-based Assays

2.1 General Cell Culture Protocol

In this study, the following cell lines were obtained from the RIKEN Cell Bank. Cells were grown in 37 ºC incubators supplemented with 5% CO_2 gas. Specific growth media used are indicated as follows:

Name	Type	Medium	FBS	Penicillin-Streptomycin
HeLa S3	human cervix adenocarcinoma cells	DMEM (High Glucose) w/ L-glutamine and phenol red	10%	1%
PC3	human prostate cancer cells	DMEM (High Glucose) w/ L-glutamine and phenol red	10%	1%
A549	human lung adenocarcinoma cells	DMEM (High Glucose) w/ L-glutamine and phenol red	10%	1%
MCF7	human breast cancer cells	RPMI w/ L-glutamine	10% (charcoal stripped)	1%

2.2 Cell Viability Studies

2.2.1 MTS Assay Protocol

Cell viability was determined using an MTS assay, which is a colorimetric method to monitor the reduction of MTS tetrazolium salts to formazan via mitochondrial dehydrogenase of metabolically active cells. The commercial kit used in this study was the CellTiter 96® AQueous One Solution Cell Proliferation Assay (Promega, Wisconsin, USA).

Based on cell titration experiments (data not shown), cells were plated and grown overnight on 96-well Falcon® microplates (1000 cells/well for HeLa; 1000 cells/well for PC3; 1000 cells/well for A549; 2000 cells/well for MCF7). The media was then removed, followed by the incubation of various concentrations of compounds used in this study. Generally, 10 µl of compounds were added to 90 µl of media. Following an incubation time of 4 days, cell viability was detected by first removing the media and replacing it with 20 µl MTS reagent and 80 µl media. Following incubation at 37 ºC for 2 hr, end-point absorbance was acquired at 490 nm, via a SpectraMax® iD3 Multi-Mode Microplate Reader (Molecular Devices, California, USA). The background control for this assay was the incubation of 20 µl MTS reagent and 80 µl media in the absence of cells. Obtained EC_{50} values were calculated via GraphPad Prism (version 7.0d) software using fitting based on the sigmoidal dose response equation.

2.2.2 Controls

Before cell toxicity studies, various controls experiments were performed. The first was to determine the toxicity of DMSO (Figure S14), which was used to dissolve the compounds used in this study. This experiment led to the standardization of 1% DMSO. The next control was to determine the toxicity of **Au-5** (Figure S15). From the acquired growth curves, it was decided that the addition of **Au-5** to a final concentration of 10 µM would be ideal for the tested cell lines (HeLa S3, PC3, A549, and MCF7).

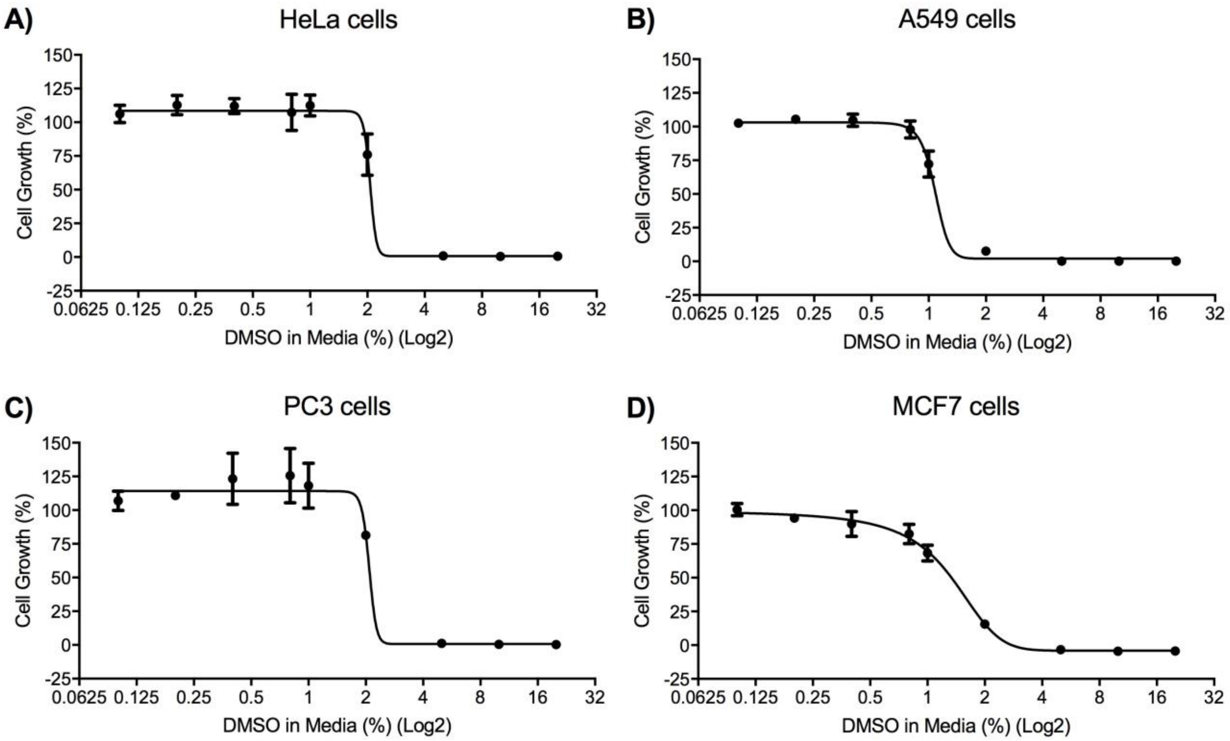

Figure S14. Cell growth curves aimed at exploring the effects of DMSO supplementation to cultures of A) HeLa cells, B) A549 cells, C) PC3 cells, and D) MCF7 cells.

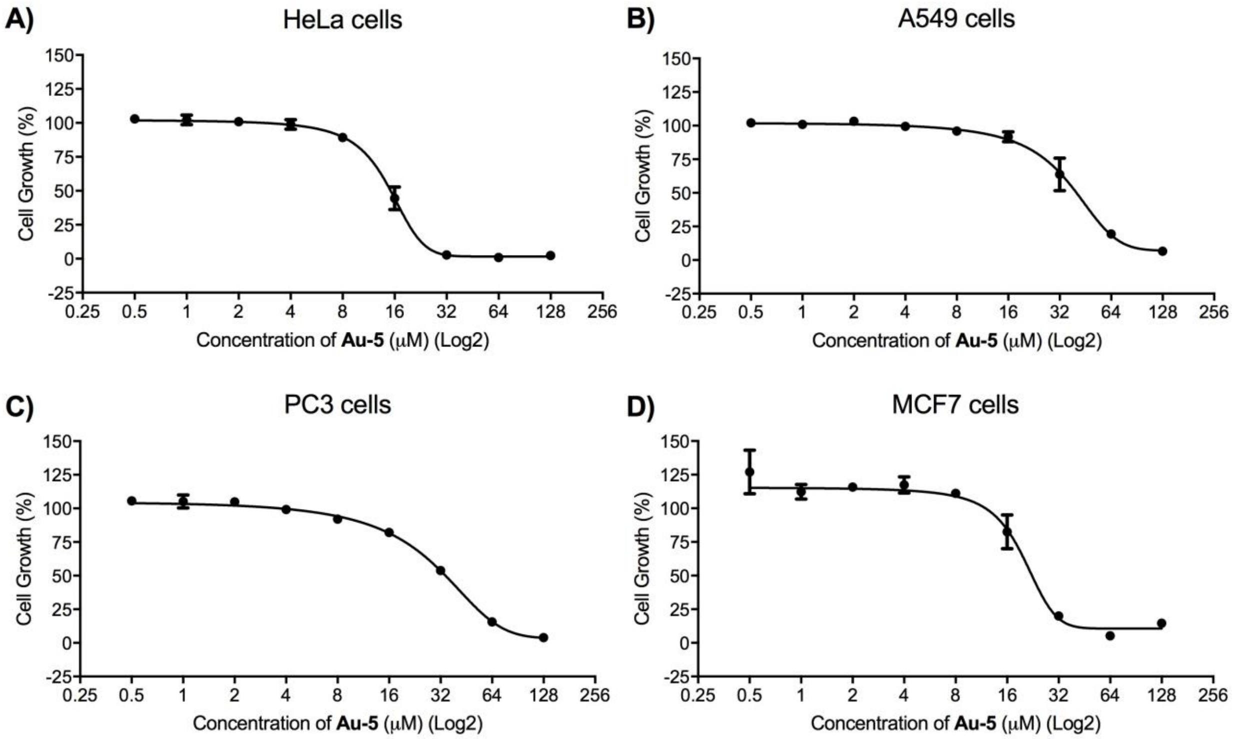

Figure S15. Cell growth curves aimed at exploring the effects of **Au-5** supplementation to cultures of A) HeLa cells, B) A549 cells, C) PC3 cells, and D) MCF7 cells.

2.2.3 *Cell Toxicity Results*

Cell growth curves to monitor toxicity of the tested compounds (**3a**, **3m**, **6a**, **6m**, and **7**) are shown in Figures S16-S19. A summary of the calculated EC_{50} values obtained in tests against MCF7 cells using prodrugs **3a,m** are shown in Table S5.

Table S5. Summary of cytotoxic activity of endoxifen-based prodrugs

Compound	EC_{50} (µM)
	MCF7
**3a**	55
**3a/Au-5**	26
**3m**	57
**3m/Au-5**	41
Endoxifen	25

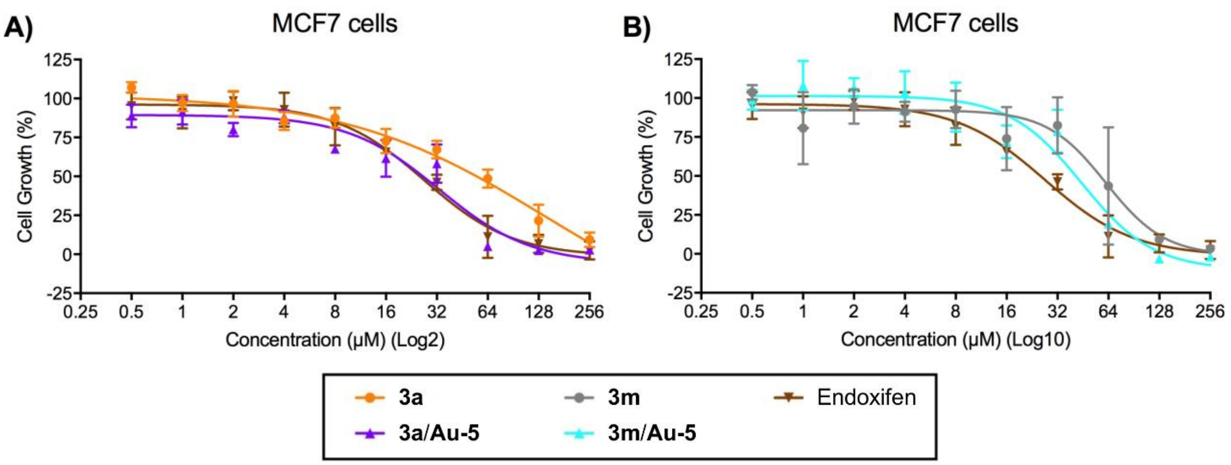

Figure S16. Cell growth curves of MCF7 cancer cells aimed at exploring the effects of supplementation with A) prodrug **3a** (orange) and mixture **3a/Au-5** (purple), as well as B) prodrug **3m** (grey) and mixture **3m/Au-5** (turquoise). Endoxifen (brown) was used as a control.

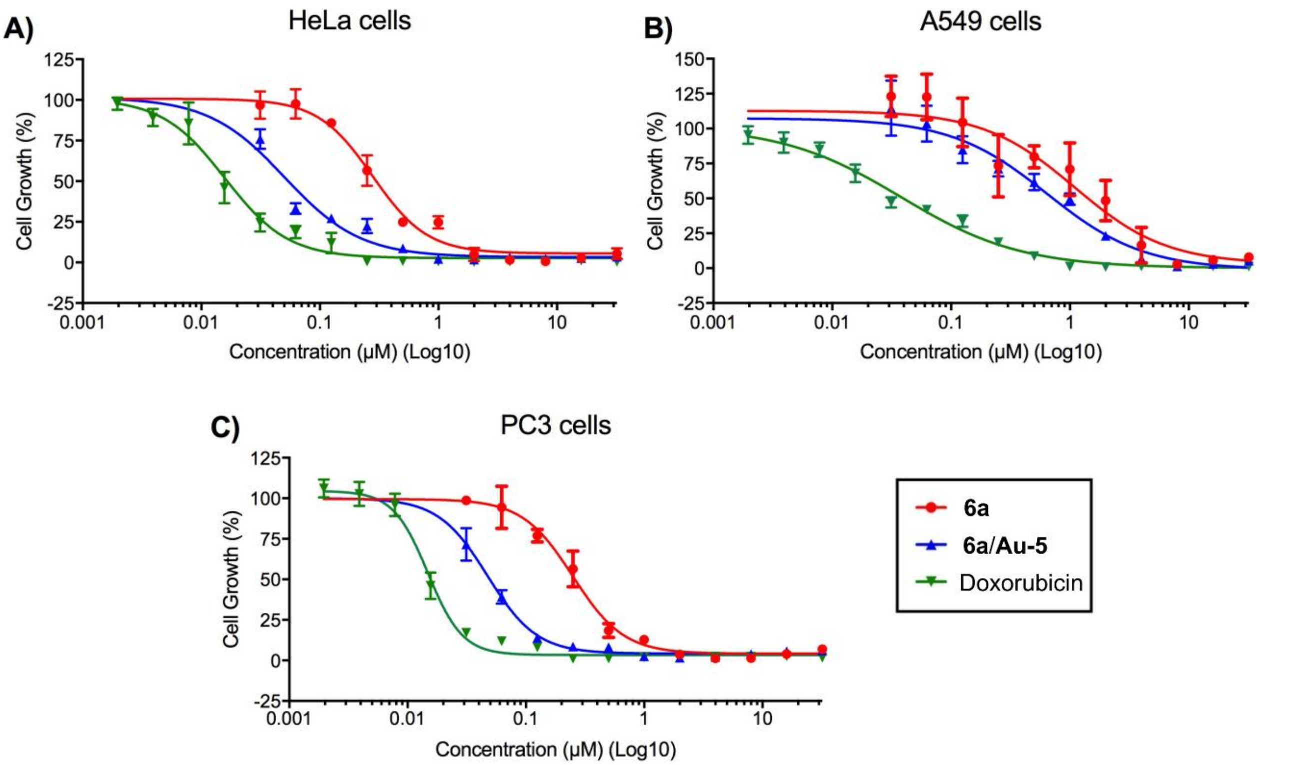

Figure S17. Cell growth curves aimed at exploring the effects of either prodrug **6a** (red), mixture **6a**/**Au-5** (blue), or doxorubicin (green) supplementation to cultures of A) HeLa cells, B) A549 cells, and C) PC3 cells.

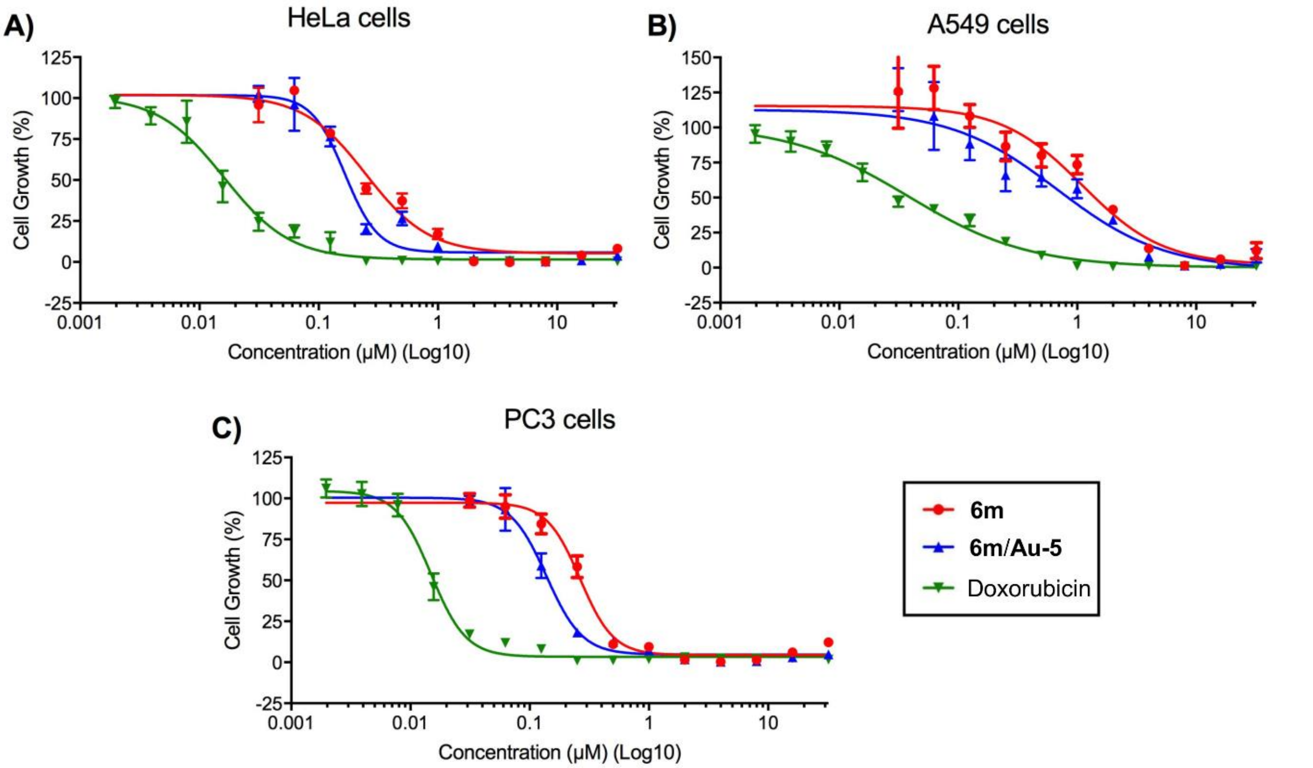

Figure S18. Cell growth curves aimed at exploring the effects of either prodrug **6m** (red), mixture **6m**/**Au-5** (blue), or doxorubicin (green) supplementation to cultures of A) HeLa cells, B) A549 cells, and C) PC3 cells.

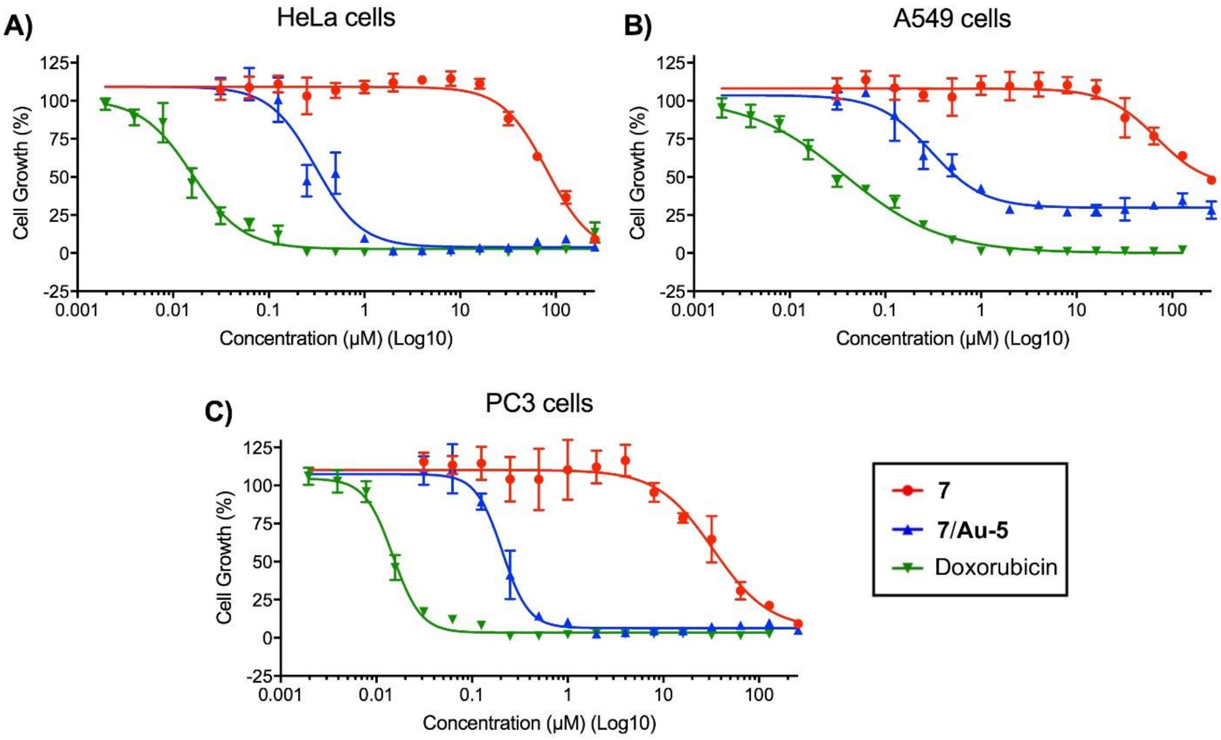

Figure S19. Cell growth curves aimed at exploring the effects of either prodrug **7** (red), mixture **7**/**Au-5** (blue), or doxorubicin (green) supplementation to cultures of A) HeLa cells, B) A549 cells, and C) PC3 cells.

2.3 Cell Permeability Studies

2.3.1 Incubation and Imaging Protocol

To monitor the penetration of compounds under study, fluorescent cell imaging was performed. Given that doxorubicin (and similar derivatives) are known to produce fluorescence (λ_{EX} = 470 nm / λ_{EM} = 560 nm), cell penetration can be comparatively quantified via fluorescent imaging of cells incubated under various conditions.

HeLa cells were first plated onto ibidi µ-Slide 8 well chamber slides at approximately 30,000 cells/well and grown overnight. In each well, 280 µl of fresh DMEM media was then added, followed by 20 µl of compound (150 µM stock solution), which gave a final concentration of 10 µM doxorubicin, **6a**, or **7**. After incubations at 37°C for set time intervals (1, 2, 4, 8 hr), cells were washed with DMEM media (2×) and PBS buffer pH 7.4 (2×). Imaging studies were then carried out using a Keyence BZ-X710 All-in-one Fluorescence Microscope® at 20× magnification. Brightfield images (color) were obtained at a 1/150 sec exposure setting and fluorescent images were obtained at a 1.2 sec exposure setting.

2.3.2 Cell Imaging Results

Cells from obtained images were randomly chosen (n=15) and their fluorescence was quantified via ImageJ. Shown in Figures S20-S31 are the cropped images of the cells used for quantification. Under set time intervals (1, 2, 4, 8 hours), incubations were carried out using either doxorubicin, **6a**, or **7**.

Doxorubicin
Time: 1 hr

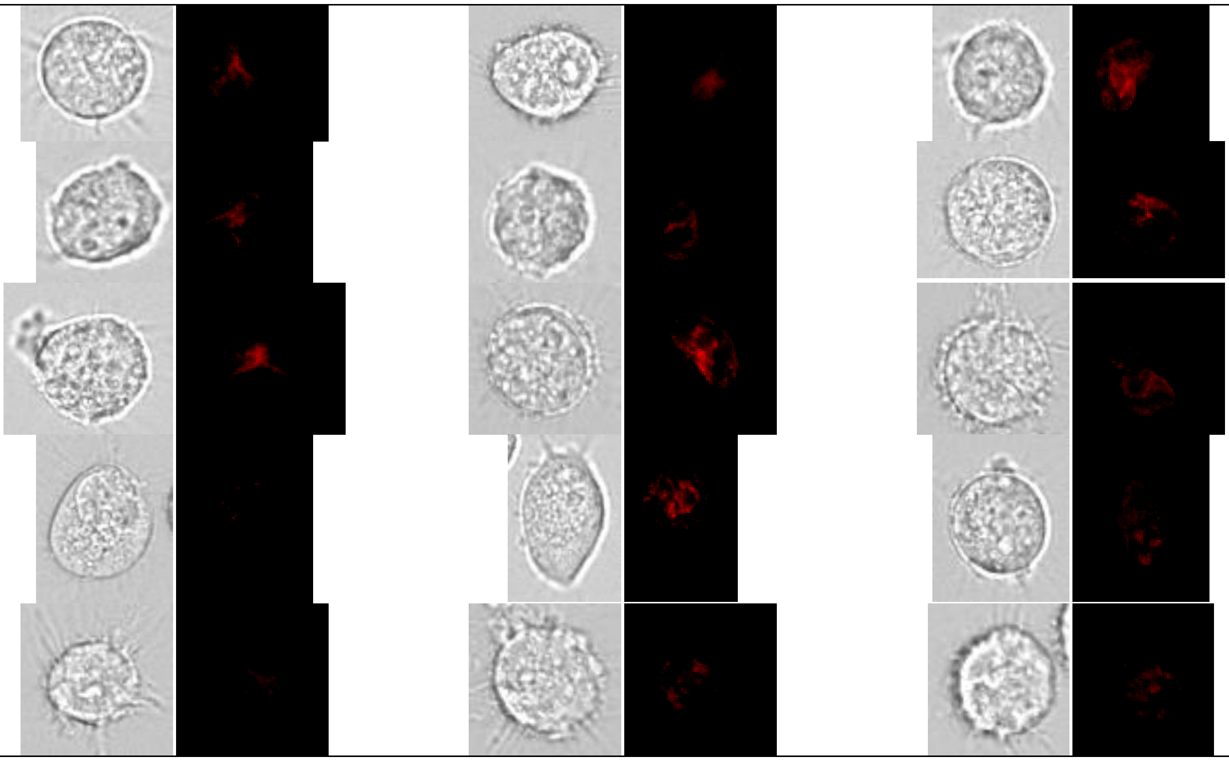

Figure S20. Imaging studies of HeLa cells incubated with Doxorubicin (10 µM) for 1 hr.

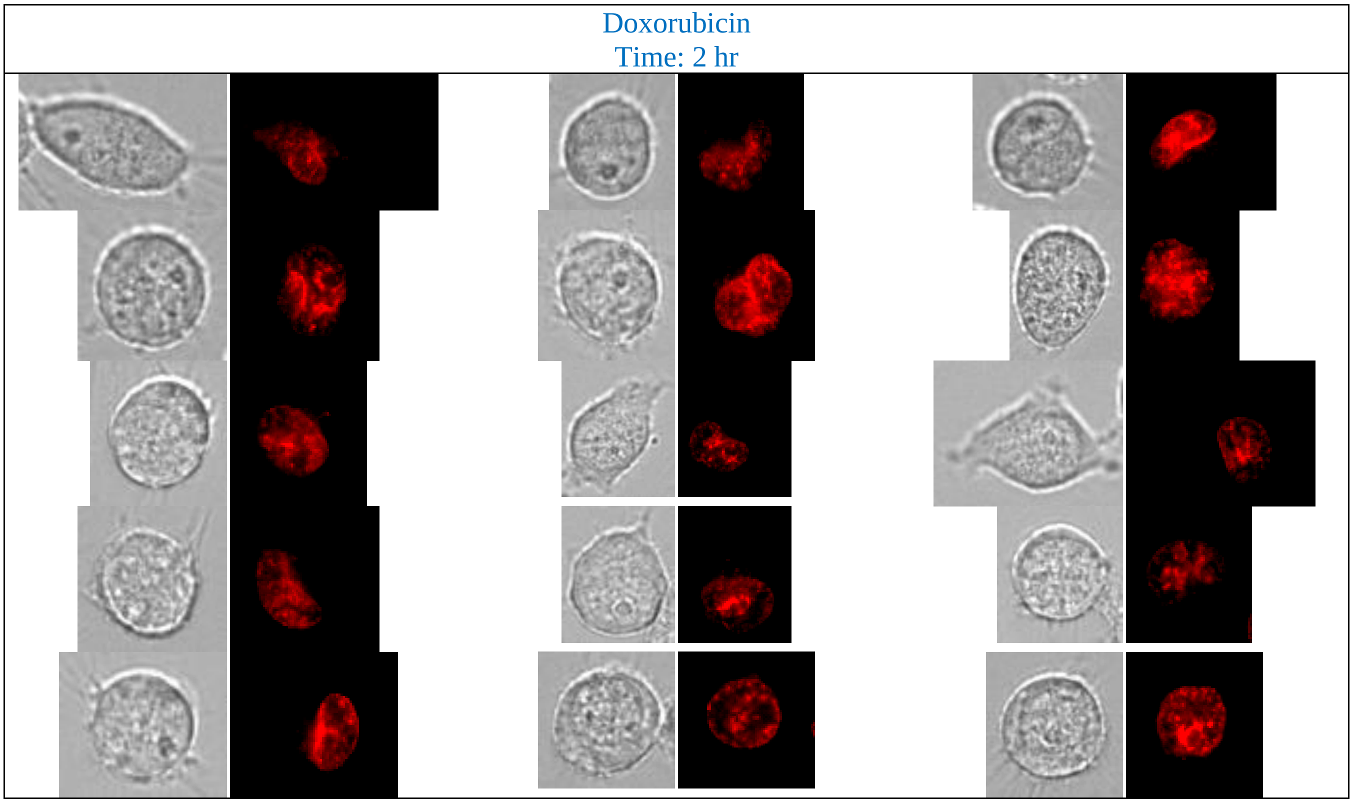

Figure S21. Imaging studies of HeLa cells incubated with Doxorubicin (10 µM) for 2 hr.

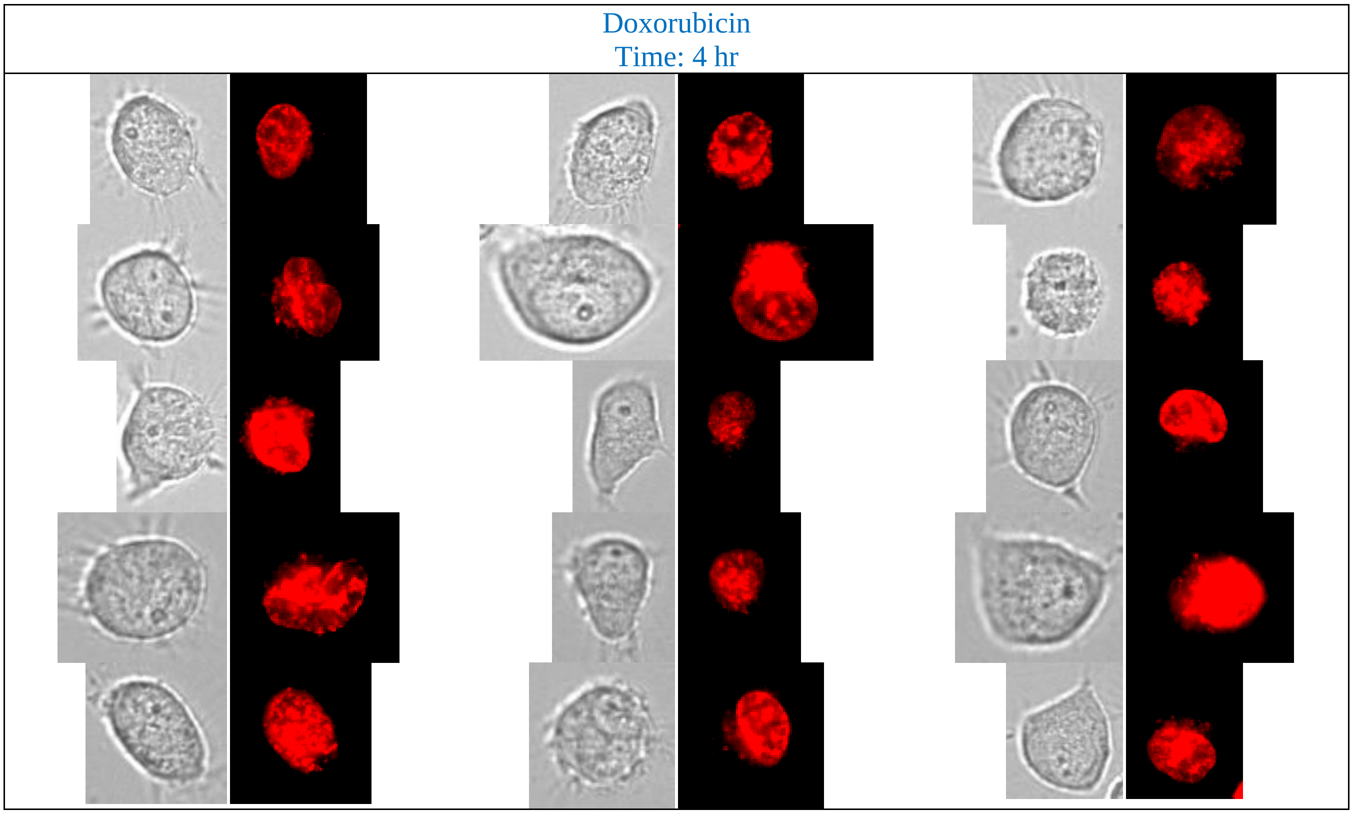

Figure S22. Imaging studies of HeLa cells incubated with Doxorubicin (10 µM) for 4 hr.

Doxorubicin
Time: 8 hr

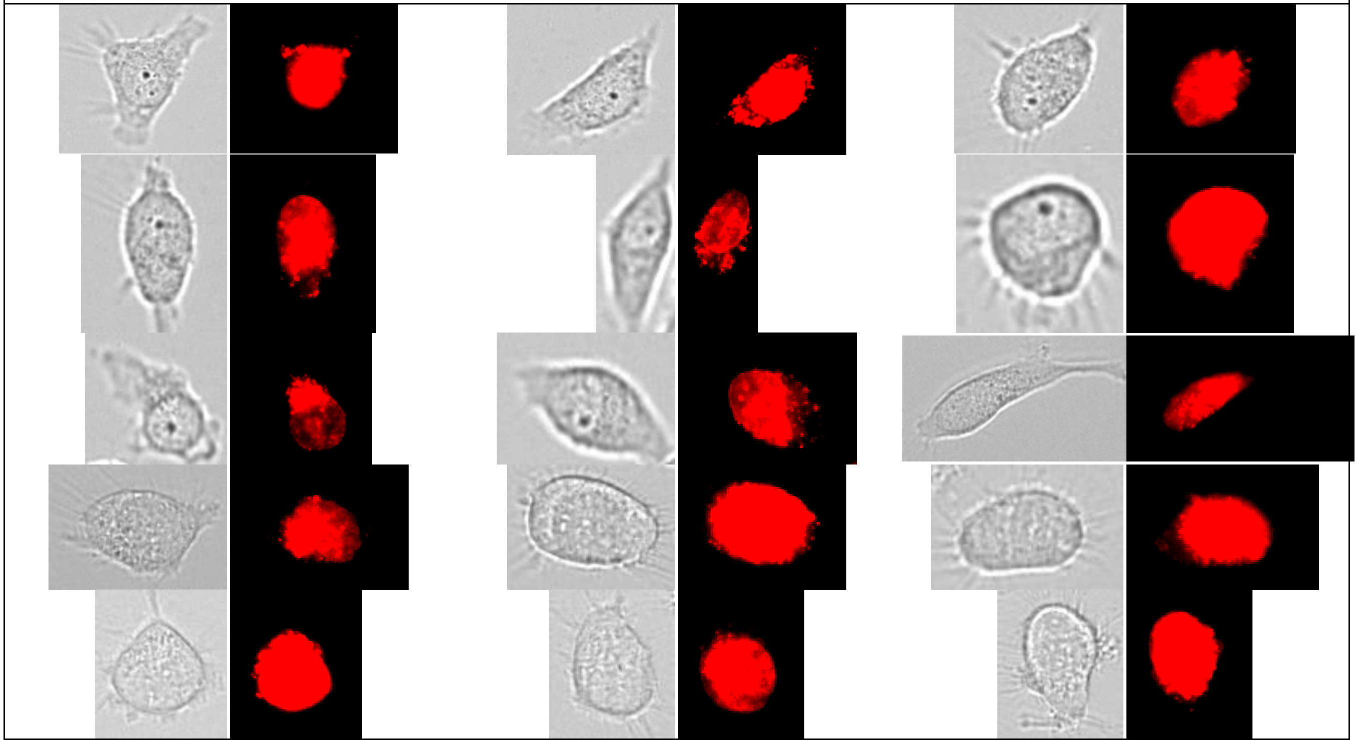

Figure S23. Imaging studies of HeLa cells incubated with Doxorubicin (10 µM) for 8 hr.

Compound **6a**
Time: 1 hr

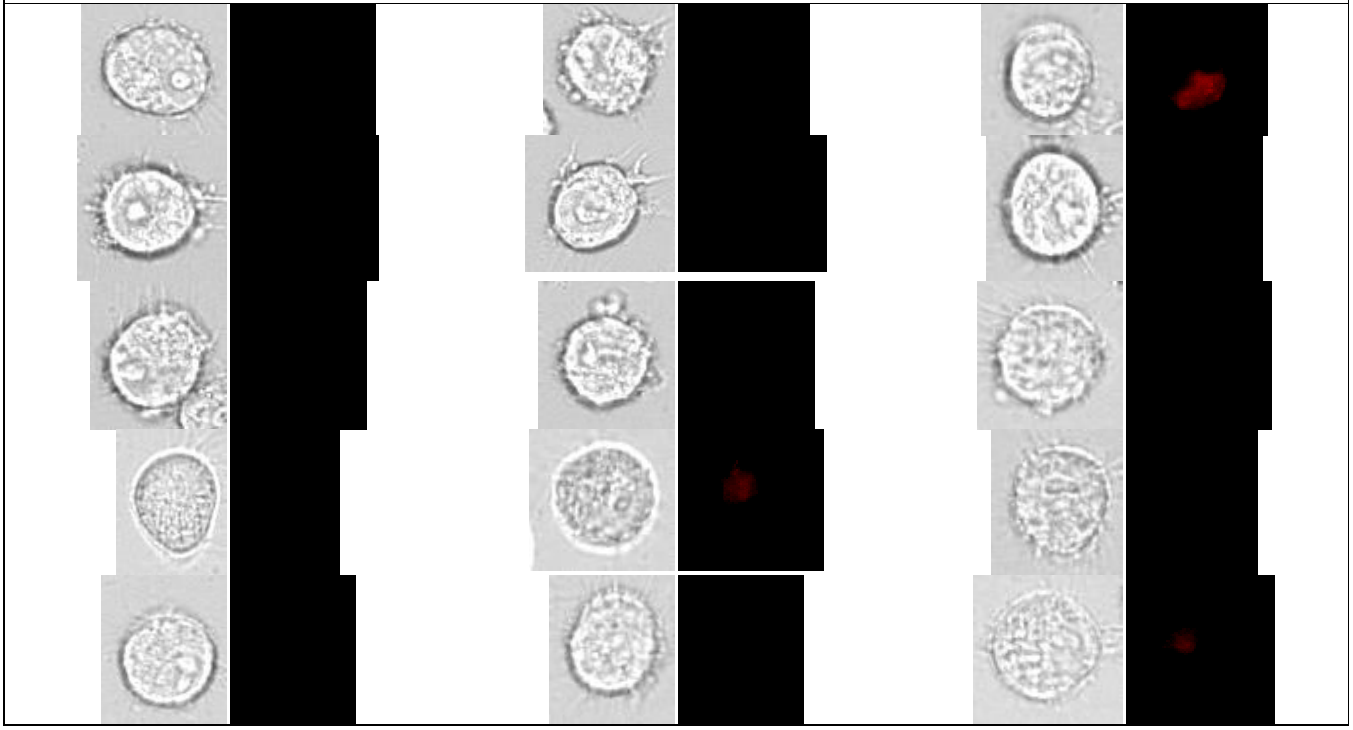

Figure S24. Imaging studies of HeLa cells incubated with compound **6a** (10 µM) for 1 hr.

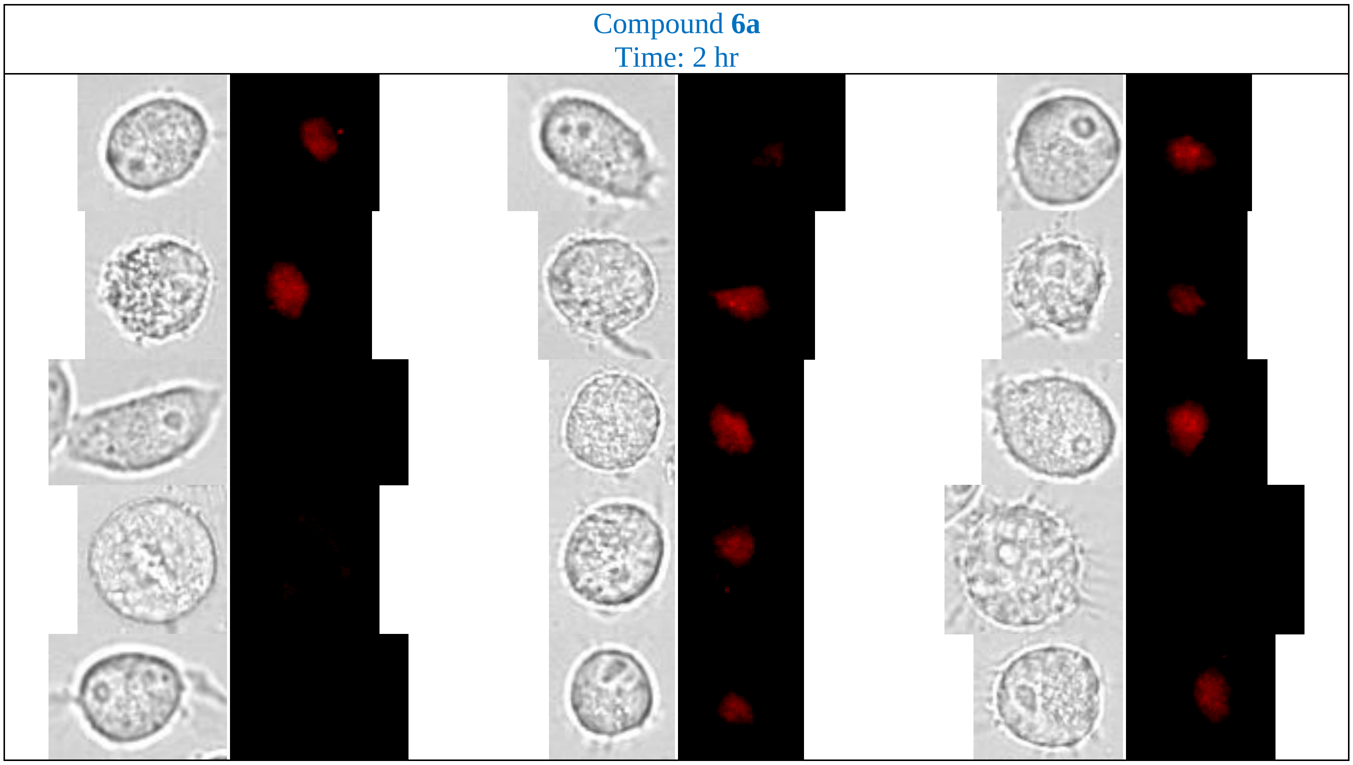

Figure S25. Imaging studies of HeLa cells incubated with compound **6a** (10 µM) for 2 hr.

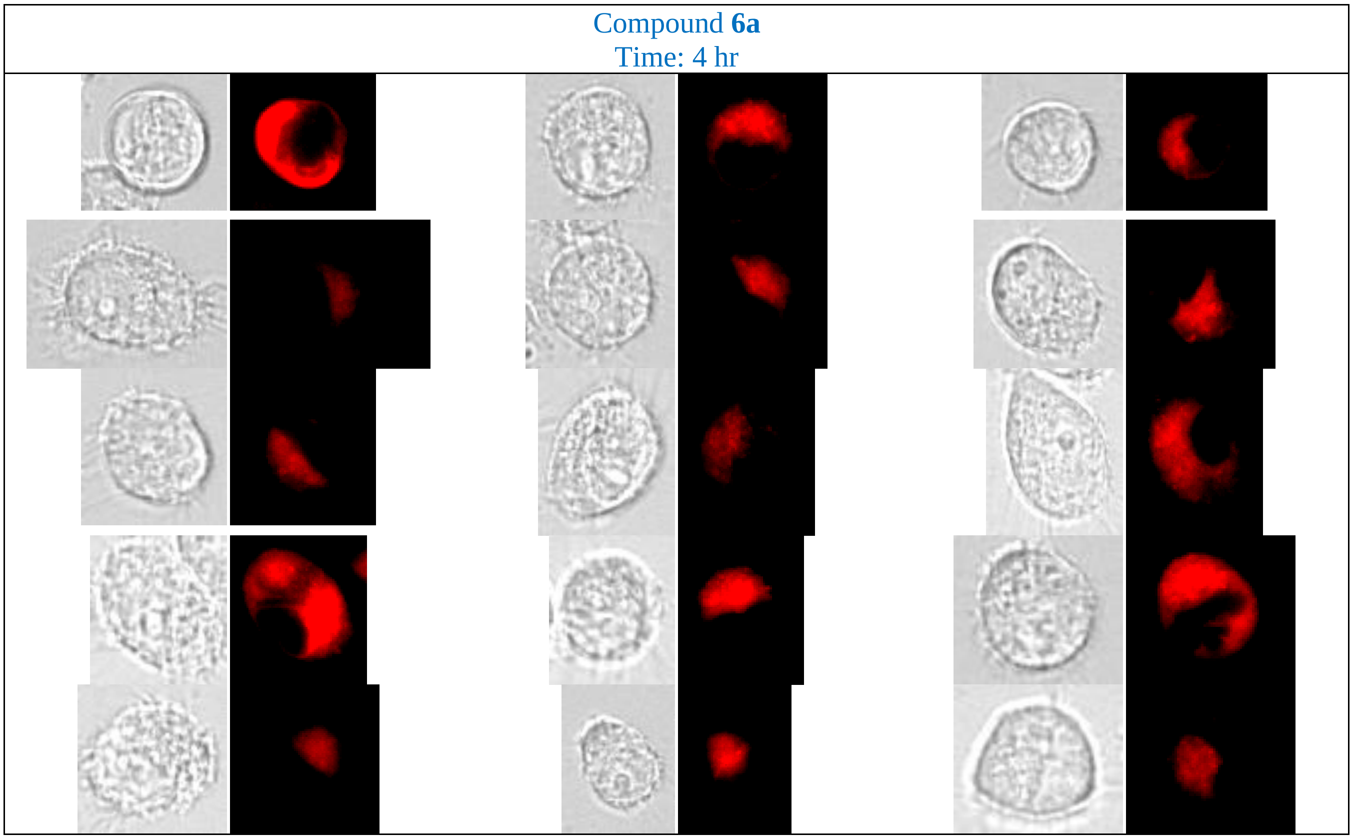

Figure S26. Imaging studies of HeLa cells incubated with compound **6a** (10 µM) for 4 hr.

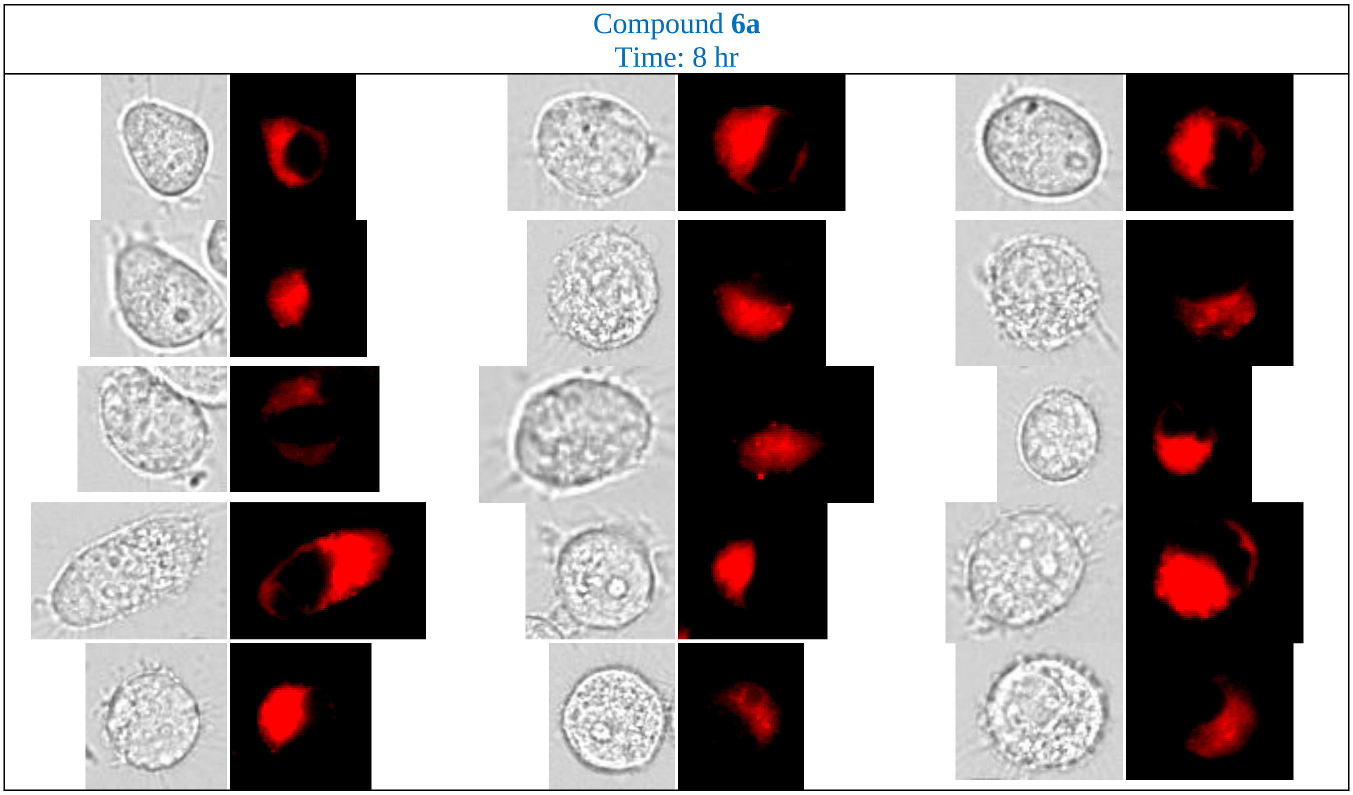

Figure S27. Imaging studies of HeLa cells incubated with compound **6a** (10 µM) for 8 hr.

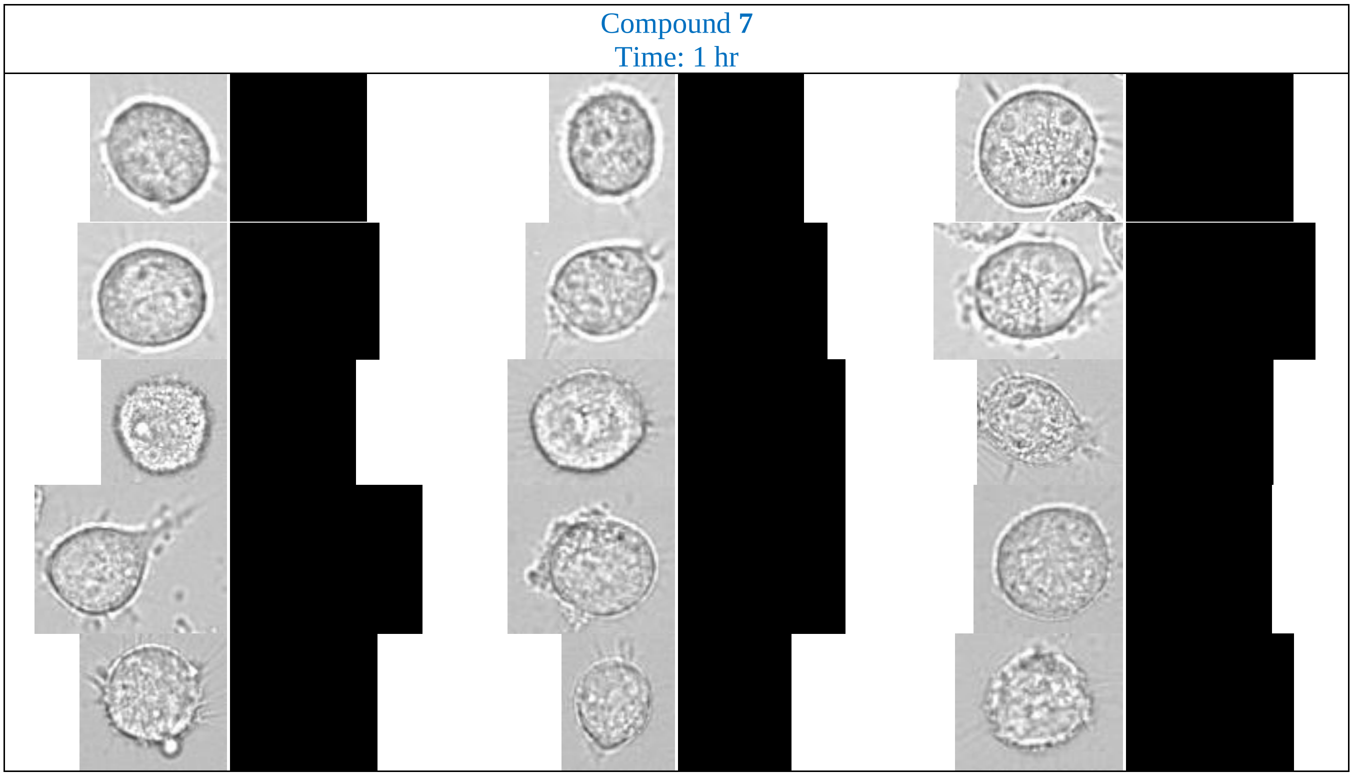

Figure S28. Imaging studies of HeLa cells incubated with compound **7** (10 µM) for 1 hr.

Compound **7**
Time: 2 hr

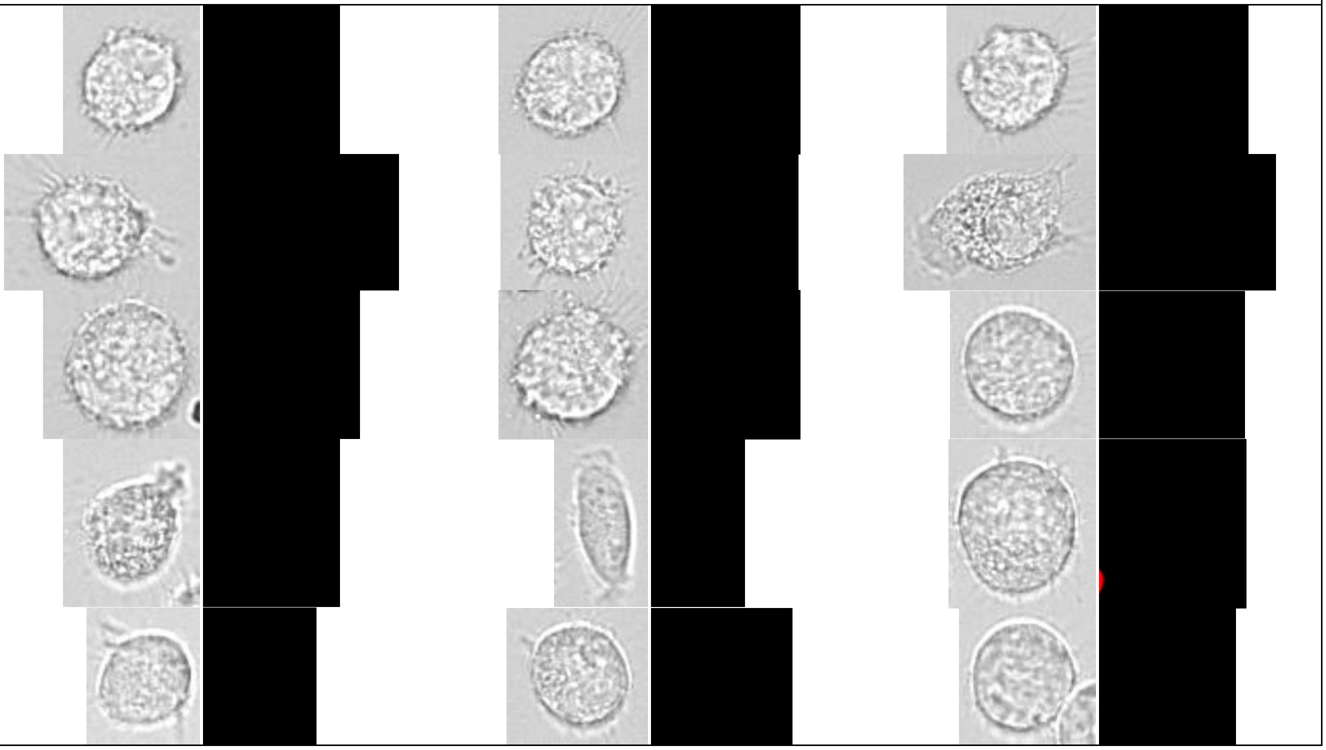

Figure S29. Imaging studies of HeLa cells incubated with compound **7** (10 µM) for 2 hr.

Compound **7**
Time: 4 hr

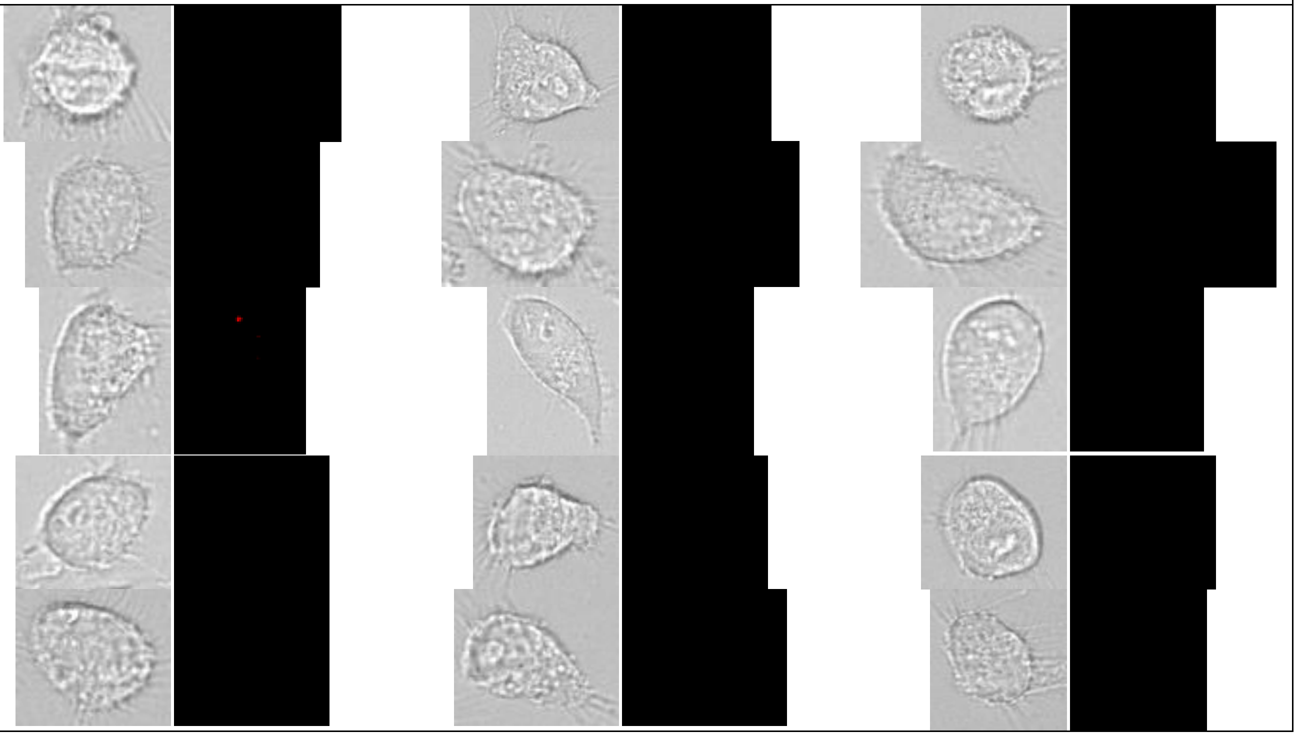

Figure S30. Imaging studies of HeLa cells incubated with compound **7** (10 µM) for 4 hr.

Compound **7**
Time: 8 hr

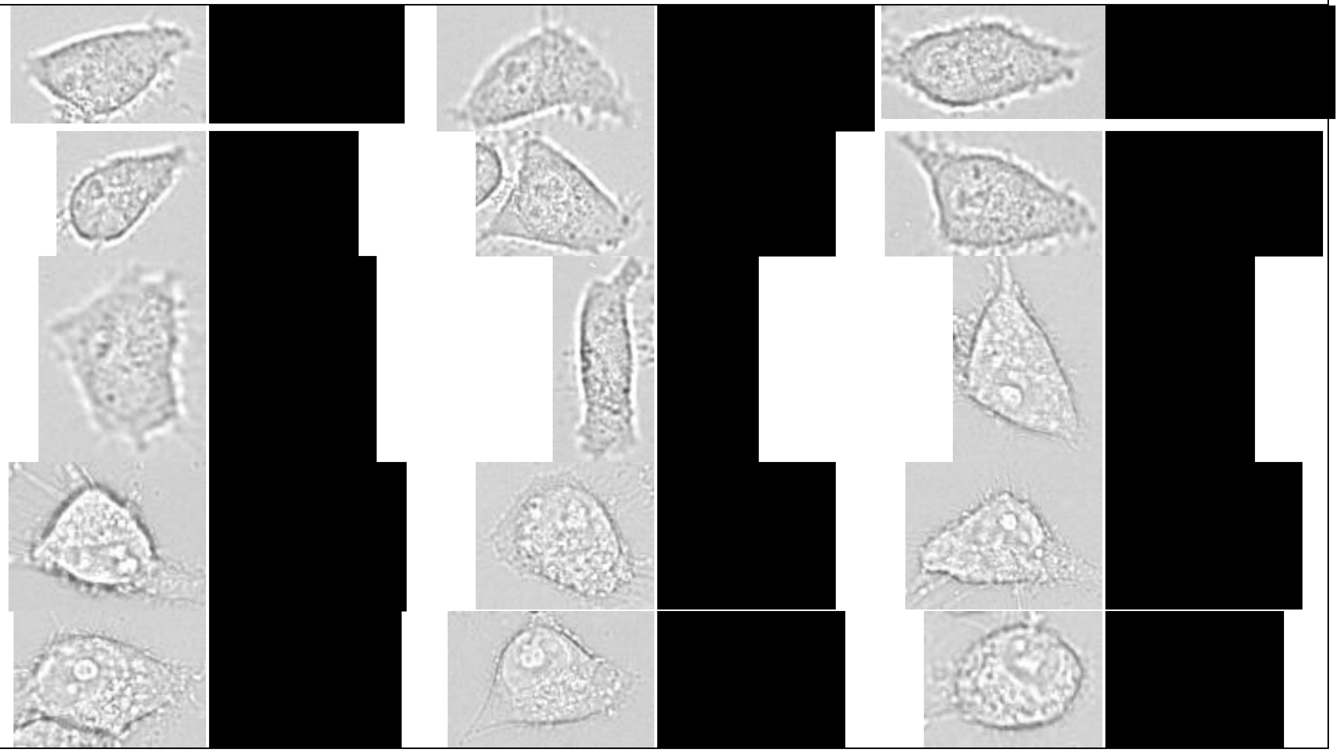

Figure S31. Imaging studies of HeLa cells incubated with compound **7** (10 µM) for 8 hr.

3. References

1. Cheng, W.; Co, E. W.; Kim, M. H.; Klein, R. R.; Le Donna, T.; Lew, A.; Nuss, J. M.; Xu, W. **2005**, WO2005039506A2.
2. Sawada, K.; Zenkoh, T.; Terasawa, T.; Imamura, Y.; Fukudome, H.; Kuroda, S.; Maeda, J.; Watanabe, J.; Inami, H.; Takeshita, N. **2007**, WO2007026920A2.
3. Peterson, K. L.; Dang, J. V.; Weitz, E. A.; Lewandowski, C.; Pierre, V. C. *Inorg. Chem.* **2014**, *53*, 6013.
4. Huy, P.; Neudorfl, J. M.; Schmalz, H. G. *Org. Lett.* **2011**, *13*, 216.
5. Thiede, S.; Winterscheid, P. M.; Hartmann, J.; Schnakenburg, G.; Essig, S.; Menche, D. *Synthesis* **2016**, *48*, 697.
6. Döbele, M.; Vanderheiden, S.; Jung, N.; Brase, S. *Angew. Chem. Int. Ed.* **2010**, *49*, 5986.
7. Letavic, M.; Rudolph, D. A.; Savall, B. M.; Shireman, B. T.; Swanson, D. **2012**, WO2012145581A1.
8. Kuduk, S. D.; Di Marco, C. N.; Chang, R. K.; Wood, M. R.; Schirripa, K. M.; Kim, J. J.; Wai, J. M. C.; DiPardo, R. M.; Murphy, K. L.; Ransom, R. W.; Harrell, C. M.; Reiss, D. R.; Holahan, M. A.; Cook, J.; Hess, J. F.; Sain, N.; Urban, M. O.; Tang, C.; Prueksaritanont, T.; Pettibone, D. J.; Bock, M. G. *J. Med. Chem.* **2007**, *50*, 272.
9. Srogl, J.; Hyvl, J.; Revesz, A.; Schroder, D. *ChemComm* **2009**, 3463.
10. Floersheim, P.; Froestl, W.; Guery, S.; Kaupmann, K.; Koller, M. **2006**, WO2006136442A1.
11. Bowers, E. M.; Yan, G.; Mukherjee, C.; Orry, A.; Wang, L.; Holbert, M. A.; Crump, N. T.; Hazzalin, C. A.; Liszczak, G.; Yuan, H.; Larocca, C.; Saldanha, S. A.; Abagyan, R.; Sun, Y.; Meyers, D. J.; Marmorstein, R.; Mahadevan, L. C.; Alani, R. M.; Cole, P. A. *Chem. Biol.* **2010**, *17*, 471.
12. Wilkinson, S. M.; Gunosewoyo, H.; Barron, M. L.; Boucher, A.; McDonnell, M.; Turner, P.; Morrison, D. E.; Bennett, M. R.; McGregor, I. S.; Rendina, L. M.; Kassiou, M. *ACS Chem. Neurosci.* **2014**, *5*, 335.
13. Spivey, A. C.; McKendrick, J.; Srikaran, R.; Helm, B. A. *J. Org. Chem.* **2003**, *68*, 1843.
14. Allart, B.; Lehtolainen, P.; Yla-Herttuala, S.; Martin, J. F.; Selwood, D. L. *Bioconjugate Chem.* **2003**, *14*, 187.
15. Cotterill, I. C.; Rich, J. O.; Scholten, M. D.; Mozhaeva, L.; Michels, P. C. *Biotechnol. Bioeng.* **2008**, *101*, 435.
16. Pérez-López, A. M.; Rubio-Ruiz, B.; Sebastián, V.; Hamilton, L.; Adam, C.; Bray, T. L.; Irusta, S.; Brennan, P. M.; Lloyd-Jones, G. C.; Sieger, D.; Santamaría, J.; Unciti-Broceta, A. *Angew. Chem. Int. Ed.* **2017**, *56*, 12548.
17. Abe, M.; Kubo, A.; Yamamoto, S.; Hatoh, Y.; Murai, M.; Hattori, Y.; Makabe, H.; Nishioka, T.; Miyoshi, H. *Biochemistry* **2008**, *47*, 6260.
18. Nierengarten, J. F.; Zhang, S.; Gégout, A.; Urbani, M.; Armaroli, N.; Marconi, G.; Rio, Y. *J. Org. Chem.* **2005**, *70*, 7550.
19. Lee, T.; Kang, H. R.; Kim, S.; Kim, S. *Tetrahedron* **2006**, *62*, 4081.
20. Percec, V.; Rudick, J. G.; Peterca, M.; Wagner, M.; Obata, M.; Mitchell, C. M.; Cho, W. D.; Balagurusamy, V. S. K.; Heiney, P. A. *J. Am. Chem. Soc.* **2005**, *127*, 15257.
21. Pandey, B. K.; Smith, M. S.; Torgerson, C.; Lawrence, P. B.; Matthews, S. S.; Watkins, E.; Groves, M. L.; Prigozhin, M. B.; Price, J. L. *Bioconjugate Chem.* **2013**, *24*, 796.
22. Jordan, J. P.; Grubbs, R. H. *Angew. Chem. Int. Ed.* **2007**, *46*, 5152.
